# Survey of residential indoor particulate matter measurements 1990–2019

**DOI:** 10.1111/ina.13057

**Published:** 2022-07

**Authors:** Vito Ilacqua, Nicole Scharko, Jordan Zambrana, Daniel Malashock

**Affiliations:** 1 Indoor Environments Division, United States Environmental Protection Agency, Washington, District of Columbia, USA; 2 American Association for the Advancement of Science (AAAS) - Science, Technology, and Policy Fellow, Washington, District of Columbia, USA

**Keywords:** home indoor particles, indoor aerosols, indoor air quality, indoor PM, literature review, residential indoor PM

## Abstract

We surveyed literature on measurements of indoor particulate matter in all size fractions, in residential environments free of solid fuel combustion (other than wood for recreation or space heating). Data from worldwide studies from 1990 to 2019 were assembled into the most comprehensive collection to date. Out of 2752 publications retrieved, 538 articles from 433 research projects met inclusion criteria and reported unique data, from which more than 2000 unique sets of indoor PM measurements were collected. Distributions of mean concentrations were compiled, weighted by study size. Long-term trends, the impact of non-smoking, air cleaners, and the influence of outdoor PM were also evaluated. Similar patterns of indoor PM distributions for North America and Europe could reflect similarities in the indoor environments of these regions. Greater observed variability for all regions of Asia may reflect greater heterogeneity in indoor conditions, but also low numbers of studies for some regions. Indoor PM concentrations of all size fractions were mostly stable over the survey period, with the exception of observed declines in PM_2.5_ in European and North American studies, and in PM_10_ in North America. While outdoor concentrations were correlated with indoor concentrations across studies, indoor concentrations had higher variability, illustrating a limitation of using outdoor measurements to approximate indoor PM exposures.

## 1 | INTRODUCTION

The public health burden of air-borne particulate matter (PM) is well established and supported by lines of evidence drawn from environmental epidemiology, toxicology, and controlled exposure studies.^1^ This abundant and consistent scientific evidence has prompted national and international standards to reduce the public health burden of PM in ambient air (used as synonymous with outdoor air). This includes regulatory standards in the United States under the Clean Air Act and its amendments, and similar regulations internationally. These regulations have generally mandated official monitoring of PM and other air pollutants to ensure statutory limits are achieved. Consequently, an extensive record of reliable, comparable, and population-representative measurements has been generated, which has contributed to increased understanding of the population-level exposure to, and burden associated with ambient PM. The majority of a population exposure to PM, however, typically happens indoors^2–4^ and in residential settings, where people spend the vast majority of their time,^5,6^ and where levels of air pollutants may differ from those in ambient air.^2,7,8^ No comprehensive record of indoor PM levels exists, however, that is comparable in terms of temporal coverage, methodological consistency, and population representativeness to that available for PM in ambient air. This comparatively limited record can be explained by several factors including legal, economic, and ethical barriers, as well as practical challenges relating to residential access.^9–11^ Underlying all these factors, the greatest methodological challenge is the heterogeneity of indoor environments and indoor air quality, both in terms of temporal variability of indoor pollutant concentrations, influenced by individual human behaviors, building materials and design, and local climate, and the fragmentation of indoor air into a multitude of microenvironments within and between buildings.

Despite the absence of a systematic and extensive indoor PM record, scientific interest in indoor air quality has sustained the measurement of PM indoors for just as long as ambient air. Numerous ad hoc research studies have measured indoor PM concentrations and have contributed to the understanding of the levels of indoor PM as well as the building-related, behavioral, geographic, and temporal factors that affect their variability. These research studies have necessarily varied in terms of goals, scope, resources, target populations, sampling and measurement methodology, study duration, observation conditions, and other important characteristics. With few exceptions, for example,^12–14^ these studies did not attempt to be truly population representative. Nevertheless, they contribute objective observations and understanding of an otherwise uncharted landscape of possible indoor air quality conditions. The information collected through measurements and modeling in existing studies allows us to understand how indoor PM characteristics and concentrations vary based on: PM in ambient air^2,14–17^; building tightness^8,18,19^; heating and cooling systems and operation^20–22^; size, geometry, and available surface area of a space^18,23,24^; the nature, frequency, and intensity of human behaviors and activities such as smoking, cooking, burning candles, or vacuuming^25–27^; the operation of windows, mechanical ventilation, and air cleaning devices^28–33^; the generation of secondary organic particles through indoor chemical reactions^34–37^; and more. Yet, the generalizability of existing studies is limited by their focus on specific geographic areas, episodic duration of measurement, and other constraints. The high cost and participant burden of large observational studies has limited more comprehensive population-level assessment. Consequently, most individual studies do not offer the panoramic view that is necessary to answer larger questions concerning PM in residential environments, such as

- What are the levels of PM found inside homes?
- How do these levels vary geographically?
- How have they changed over the years?
- How much does outdoor PM contribute to indoor PM?

To contribute to addressing these questions, we surveyed available literature on indoor PM measurements in residential environments worldwide and assembled a comprehensive collection of measurement data. These data can be used to improve exposure estimates for air pollution epidemiology and risk assessment, allow comparative analyses, inform the development of indoor air quality products, and provide context for policies targeting the role of the built environment in public health.

We focused this review on studies reporting indoor PM measurements, worldwide but limited to residential environments comparable to those in the United States and other high-income countries. Indoor environments across the world share several characteristics including the limited rate of air exchange between indoor and ambient air, a low level of solar radiation, effective barriers against precipitation, high surface to volume ratio, and complex systems to control temperature, supply water and energy, and remove water-and air-borne waste. These similarities among residences around the world, however, are no longer consistent in buildings below a certain economic status, and specifically below the point on the energy ladder where solid fuels or biomass are used. The public health burden of indoor air pollution where solid fuels are used overshadows that of other air pollution exposures,^38^ offering little insight applicable to managing indoor air pollution in high-income countries. Rather than exclude regions of the world where biomass fuels are prevalent, which could miss data from many suitable buildings from regions with great economic disparity, we opted to exclude measurements where solid fuels use was reported. As an exception to this, we still included data from homes reporting wood burning for recreation or space heating, which are common particularly in colder climates, regardless of economic status.^39^

Review articles on the topic of air-borne particles in residential indoors environments do exist. To the best of our knowledge, however, no single existing literature review covers the wide range of published studies that are included in this survey. Some of the published reviews are narrative in nature and provide an inventory of air-borne particle concentrations from the individual studies and refrain from pooling data across studies in a quantitative manner.^40–42^ Table S1a summarizes existing literature reviews that do not provide pooled summary statistics. Other reviews carried out analyses of the data across studies and reported corresponding summary statistics. Those reviews are summarized in Table S1b, which also includes the researchers’ quantitative approach for obtaining pooled statistics. Most of the summary statistics were obtained by weighing the number of measurements within each study.^43,44^

## 2 | METHODS

### 2.1 | Literature search strategy

Peer-reviewed publications were collected from *PubMed* and *ISI Web of Science* databases. Search strategies were optimized by trial and error to privilege completeness, even at the cost of a higher rate of irrelevant results. Publications in any language were searched, if at least an English-language abstract was available in the databases. The two primary search criteria for publications included keywords identified anywhere in the publication (title, abstract, and text) referring to PM (including synonyms and subtypes) and to indoor environment (and synonyms). A requirement for mentions of measurement (and synonyms) was included to decrease results only reporting modeling, policy perspectives, or commentary on the topic. Results were then limited to residential environments (including community residences, like dormitories and retirement homes) through a broad range of descriptors. Finally, articles primarily concerned with household use of solid fuel combustions (limited to mentions in abstract or title) were excluded. The search criteria are listed in Table 1, without consideration for the specific syntax required of these databases. The ability of the search to retrieve a predetermined set of relevant papers was also tracked throughout the search development process to ensure the search sensitivity was in agreement with study goals.

**Figure 1.**
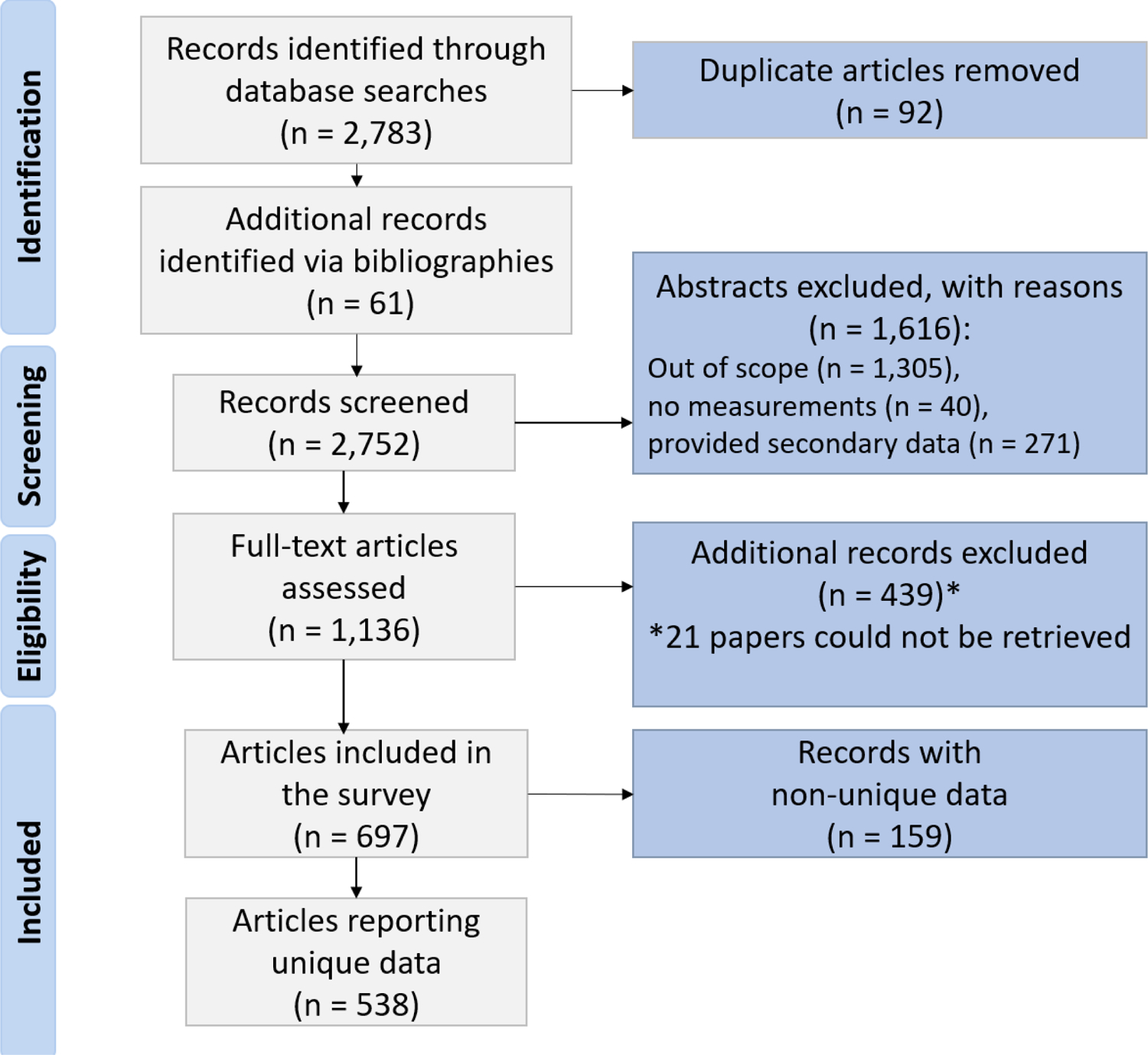
Results of literature search and papers review

Collected abstracts were screened by human readers to exclude irrelevant results. Publications whose relevance to the study could not be determined from their abstract alone were included, along with those articles deemed relevant, for full-text review. As a quality control measure, abstracts not selected for full-paper review were evaluated by a second reviewer, and in cases of discordant opinion regarding study eligibility, were included for full-text review. The results of this process of literature search and publication review are shown in Figure 1.

### 2.2 | Data extraction

Publications selected for full review were evaluated by a human reader against inclusion criteria. Data from publications deemed relevant were extracted using a standard operating procedure and entered into a database using standard forms designed to minimize data entry errors. Reasons for exclusion were noted. Additional possibly relevant papers not captured by the original searches were obtained from the literature cited. Papers resulting from a single research project (e.g., RIOPA, EXPOLIS, and French Building Survey) were characterized in the database. Relationship to an existing research project was discerned by the reviewers based on mention of a project name in text or dates, author names, sampling locations, and grants acknowledged. In a few cases, where the same researchers published results on sampling cohorts over longer time spans and with multiple sources of funding, distinctions between projects were unclear and results from different papers were attributed to separate projects. Relevant papers could be traced to 433 different research study projects. Primary data extracted included statistics on PM concentrations for sets of measurement samples reported. Some papers reported results for multiple sets of samples, distinguished by type (e.g., TSP and PM_2.5_), geography, location (i.e., indoor, outdoor), season, time of day, or other criteria used to establish contrasts (e.g., cooking vs. not, proximity to road, and weekday vs. weekend).

Journal articles were examined for use of established sampling methodology, but the size of this survey (number of included publications) prevented more rigorous evaluation of individual study methodology, including data quality evaluation and classification that may be used as a weighting factor. Authors’ and peer-reviewers’ assessment of the adequacy of the methodology to measure PM concentrations was not questioned during the data-extraction phase. However, outlier concentrations were flagged and checked for special circumstances during sampling and possible methodological flaws. It is also worth noting that the data extraction process in this survey is limited to the measurement results as reported and ignores the interpretations of results offered by authors to address individual study goals, where much of the variability in quality between individual publications resides.

Indoor PM concentrations measured by fixed or portable samplers or monitors, in any number of indoor residential environments, for any length of time, and with any analytical method were extracted. In addition, indoor concentrations resulting from personal exposure studies were extracted, if the authors reported the calculated concentrations for the residential indoor environments and the average time spent in that microenvironment. Outdoor concentrations were extracted from both on-site paired outdoor air sampling (labeled as “Outdoor (on site)” in the results) and paired off-site ambient air monitoring (“Ambient”), often a regulatory monitor.

Concentrations of any particle size fraction or other physical characteristics (number, surface area) were extracted. Ultrafine particles (UFP) were variously defined, but most commonly as those with an aerodynamic diameter less than 0.3 μm, so we used that cutoff size in this study. Particle number concentrations were most often reported without minimum and maximum cutoff sizes, and we therefore grouped them together, but methodological differences across studies are likely to affect number concentrations (of unspecified size) more than mass concentrations. Number concentrations for specific sizes (other than UFP) were too heterogeneous and sparse to be analyzed.

Measures of central tendency of PM concentrations (mean, median, and geometric mean) were extracted as available, as well as second moments (e.g., standard deviations) and extreme values (minimum, maximum). For most analyses, we grouped study locations into a few macro-regions, based on climate, economic development, ambient air pollution history, and spatial contiguity, acknowledging that all such groupings are arbitrary to some degree.

### 2.3 | Statistical analyses

The analysis of data collected from a variety of studies with different methodologies, time scales, and data distributions requires establishing a framework with explicit assumptions to avoid introducing the risk of improper inference. We defined the statistical universe (or population) of interest as the concentrations in all residential environments, at all points in time. This statistical space was sampled by individual measurements of PM concentrations (almost always) in a single spot within selected indoor environments, under the assumption that these environments are sufficiently homogeneous over the time scale of the measurement. This assumption may introduce a bias, but the potentially much bigger source of bias is the general lack of randomness in the overall sampling, leaving open the possibility that certain regions of the statistical universe are either ignored (e.g., locations less accessible by researchers; holiday periods) or oversampled (e.g., homes of researchers themselves; evenings after-work hours). This is an intrinsic limitation that should be kept in mind in interpreting all results. All statistical analyses were performed in R 3.6.1^45^ (through R Studio 1.2).

#### 2.3.1 | Calculated means and excluded measurements

Publications reported central tendency estimates for Indoor PM measurements in several different ways, depending on their data and purpose. The arithmetic mean was reported for more than 80% of the indoor measurements, but only the median for three quarters of the remaining measurements, and the geometric mean for the rest. For consistency of presentation, and to meet the requirements of some analyses, we calculated mean values, where possible, for the measurements that did not report them. Exact formulas exist to calculate the arithmetic mean from the geometric mean and geometric standard deviation.^46^ In other cases, the mean was estimated from the median, sample size, and extreme values using the methods by McGrath et al.^47^ with the R package *estmeansd*.^48^ The performance of this latter method was evaluated by comparing estimated means to reported means in cases where both were available. The results were satisfactory (Table S2): The median relative error was +2.0% (so a slight bias toward overestimation), the median absolute relative error was 6.4%, and its (outlier) maximum was 376% (for a particle number). About 8% of means used in further analyses were calculated. Almost 13% of the measurements (indoor or outdoor) did not provide enough information to estimate the mean and were excluded from the work presented here.

#### 2.3.2 | Weighting

The total duration of measurements in the included studies varied by over 6 orders of magnitude in scale, from measurements lasting a few minutes in a single home, to large field campaigns monitoring concentrations over weeks or months in hundreds of homes. The ability of a study to capture the variability of indoor PM (between homes and over time) necessarily reflects this range, though even small studies do contribute to our understanding. Throughout the statistical analyses performed, from between-study descriptive statistics to regressions, we therefore used weights proportional to the total amount of time sampling was performed, unless otherwise noted. These weights can be most easily understood as the product of the number of homes in a sample and the average duration of sampling in each home over the study, expressed in units of buildings × hours. Figure S1 shows the distribution of this weighting factor. To avoid possible confusion, the expression *weighted regression* in this work uses the weights above and does not refer to the commonly used approach of weighting by the inverse of the standard deviation, which in our case can reflect actual variability more than measurement error.

#### 2.3.3 | Regressions

Long-term temporal trends and the role of outdoor concentrations were explored through multiple regression, controlling for other factors, such as different locations, absence of environmental tobacco smoke (ETS), or use of air cleaners when possible. Multiple regressions are general and flexible tools, but ordinary least-squares linear regression was generally not appropriate for our data since the assumption of normality of the residuals was usually violated. Indoor concentrations (the dependent variable) were approximately log-normally distributed, as is common with PM concentrations,^46,49^ as were the residuals. Different approaches can be used,^50^ and often the assumptions of normality and homoscedasticity can be ignored by relying on the central limit theorem, but in many cases our sample sizes were fairly small. Another common approach, the log transformation of the data, has been shown to provide biased estimates for log-normally distributed data.^51^ This also has the drawback of less intuitive multiplicative interpretation of results, rather than absolute change. We therefore addressed these issues with a non-linear regression with lognormal error distribution^52^ and identity link, with the R package *logNormReg*.^53^ All regressions were weighted as described above. The implications for the results of using this approach rather than a linear model or a log-transformation can be seen as an example in Figure 8, where the same data are fitted using different approaches. Although all methods perform similarly near the mean, both slope and intercept are affected by the choice.

There is no universally accepted way to assess the fit of a model, or the amount of variation explained in a non-linear model. With a non-linear regression, the coefficient of determination *R*^2^ can no longer generally be used,^54^ because the residuals sum of squares (RSS) and the regression model sum of squares (MSS) do not add up to the total sum of squares (TSS). However, when TSS ≈ MSS + RSS, a pseudo-coefficient of determination that preserves its definition as $R_{\text{d}}^{2}=\text{MSS}/\text{TSS}$ could still be informative, and more readily interpreted than other goodness of fit measures. Since this was the case in almost all our regressions, we reported a $R_{\text{d}}^{2}$, along with its distortion (RSS + MSS – TSS)/TSS. A positive distortion value indicates $R_{\text{d}}^{2}$ exceeds the true fraction of variance explained and a negative value indicates it falls short of it.

Regressions using the larger geographic groupings (e.g., regions) assume a homogeneity of the residuals variance among groups, which may not be the case. On the contrary, pooling together more observations can help estimate additional parameters. Our approach, therefore, was first to perform regressions using smaller geographic units (e.g., country), when numerous observations were available. Then, for regional estimates, we pooled together observations from the largest groups we could build that failed to reject the hypothesis of homogeneous residuals variances with the Fligner–Killeen non-parametric test. When more limited numbers of observations were available, we simply performed pooled regressions first, and then separate regressions on sub-groups when possible.

Regressions of indoor concentrations to outdoor (or ambient monitor) concentrations can have a physical interpretation. The time-varying PM concentration indoors can be approximated^55^ by

(1) $$\frac{\text{d}C_{\text{in}}}{\text{d}t}=aPC_{\text{out}}-aC_{\text{in}}-kC_{\text{in}}+\frac{S}{V}$$

(2) $$C_{\text{in}}=\frac{S}{V(a+k)}+F_{\text{inf}}C_{\text{out}}+\frac{1}{a+k}\frac{dC_{\text{in}}}{dt}$$

(3) $$C_{\text{in}}=\beta_{0}+\beta_{1}C_{\text{out}}+\varepsilon$$

where *C*_in_ and *C*_out_ are the indoor and outdoor concentrations, respectively, *a* is the air exchange rate, *P* is the fraction of particles penetrating the building envelope, *k* captures the sum of all processes removing particles indoors (deposition or filtration), and *S* is the particle emission rate from all indoor sources in an indoor space of volume *V*. The fraction *F*_inf_ = *aP*/(*a* + *k*) is often referred to as infiltration factor, representing the ratio of competing processes adding and removing particles from the outdoors. Rearranging (1) into (2) yields an equation that can be written as a regression model (3),^56^ where β_0_ and β_1_ are the coefficients to be estimated by the regression. To the extent that measurements are intended to yield representative mean concentrations, the time derivative term is small and random, and can be considered part of the error (*ε*) in the regression models.

## 3 | RESULTS AND DISCUSSION

### 3.1 | Literature search results

A total of 2844 publications were retrieved from literature searches, and the selection steps and results are shown in Figure 1. To improve readability and facilitate source tracking for the unusually large number of references used as data sources, the publications have been organized geographically and by PM size fraction in Table S5, to match results as presented. Results for each region or country in the analyses below make use of all data available in the references for that region or country.

**Figure 2.**
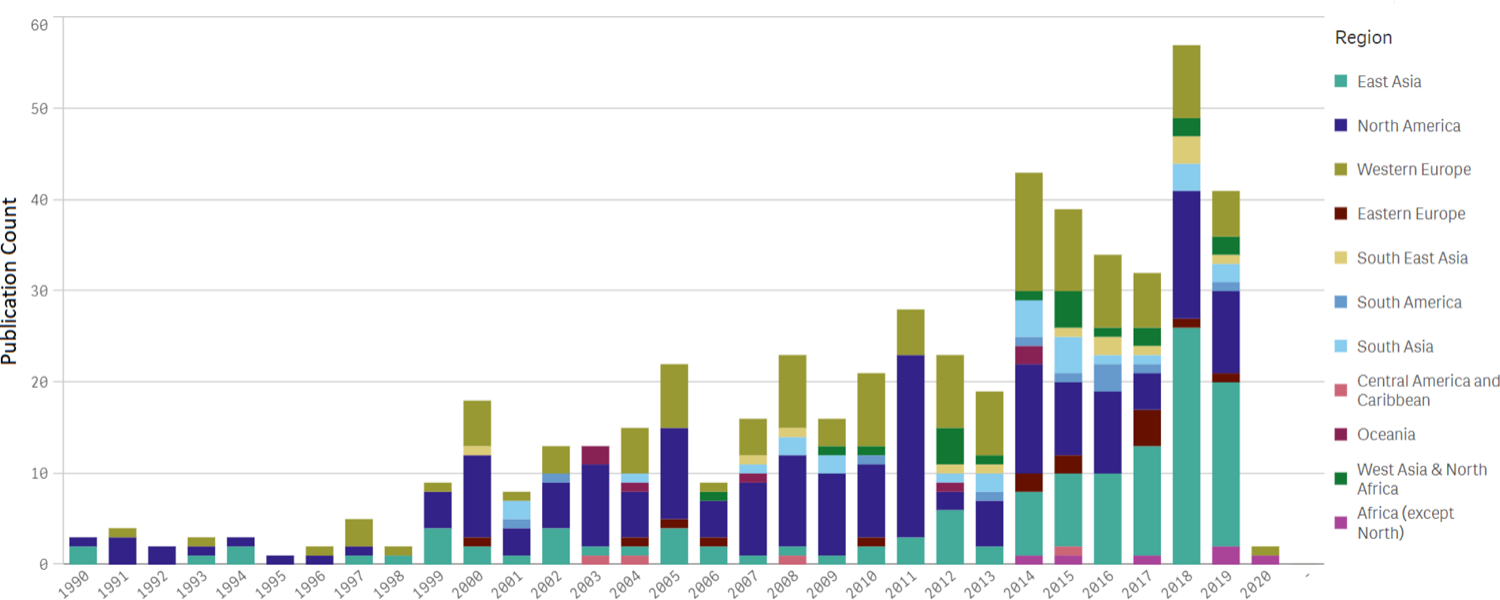
Region-specific publications with data included in the survey. Some journal articles with a 2020 date of publication were available in 2019.

On average, 1.6 published papers were found about each research project. The number of published papers per decade reporting data on indoor PM increased by a factor of 9 over the timespan of this survey (Figure 2), from 45 in the first decade (1990–1999) to 413 in the latest (2010–2019). This increase is faster than the general growth rate in scientific journal article publications (by a factor of 3 for PubMed index),^57,58^ suggesting a growing interest in indoor air quality, especially in East Asia.

**Figure 3.**
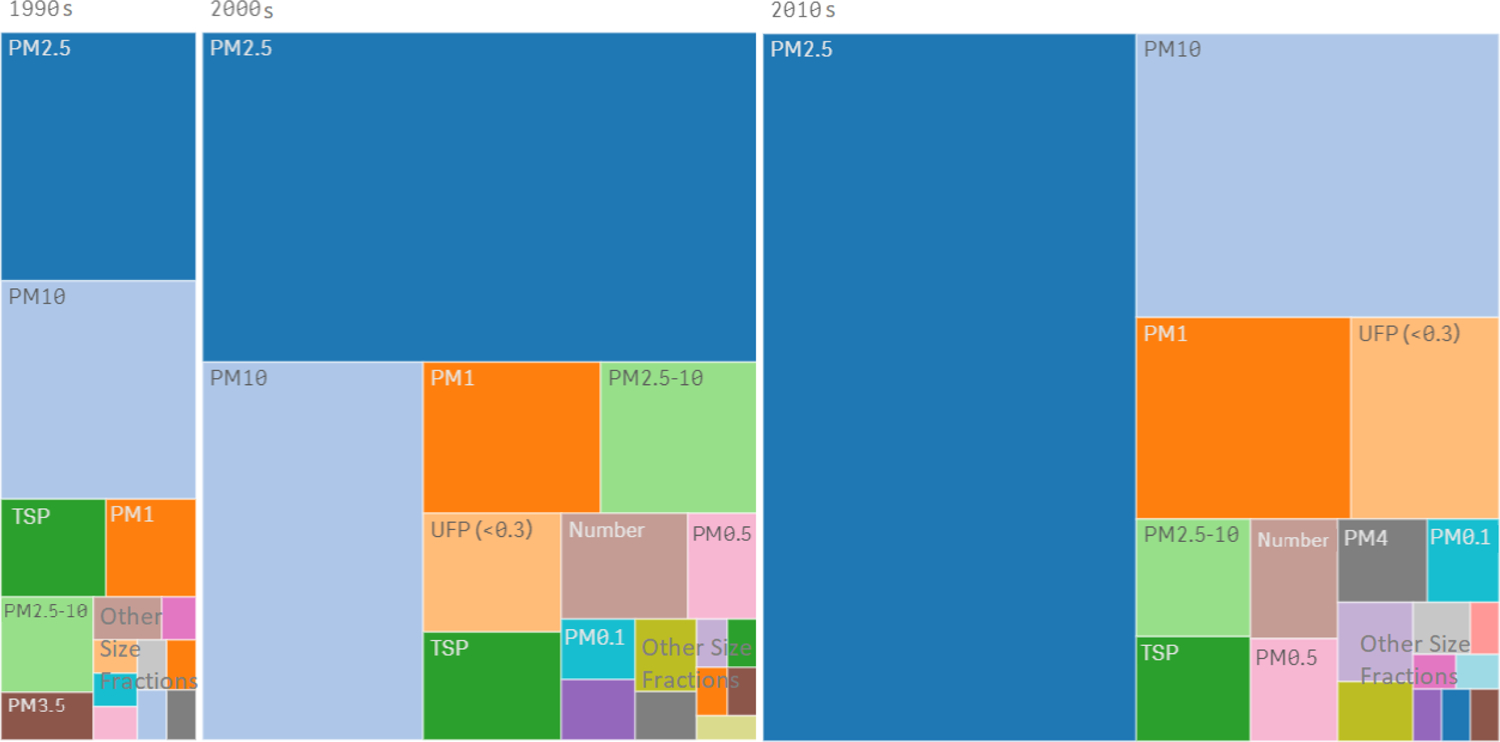
Different PM size fractions (and other metrics) investigated over the decades by the literature surveyed. Areas are proportional to the number of studies. Studies involving multiple species are included multiple times. Other size fractions include PM_2_, PM_2–10_, PM_5_, and more.

The degree of interest in different PM sizes is reflected in the number of research projects and publications investigating them (Figure 3), with PM_2.5_ claiming the greatest attention of researchers and twice as much as PM_10_, the next most researched fraction. Interest in other sizes or properties changed over time. In a few cases, investigators opted to measure unusual size fractions (e.g., PM_0.5_, PM_3.5_, and PM_>4_), or reported actual cutoff sizes they verified for their samplers. Where possible, these sporadic results were combined with similar size fractions (e.g., PM_0.25–2.5_ mass with PM_2.5_ mass), but otherwise proved to be too infrequent to lend themselves to further analysis here.

**Figure 4.**
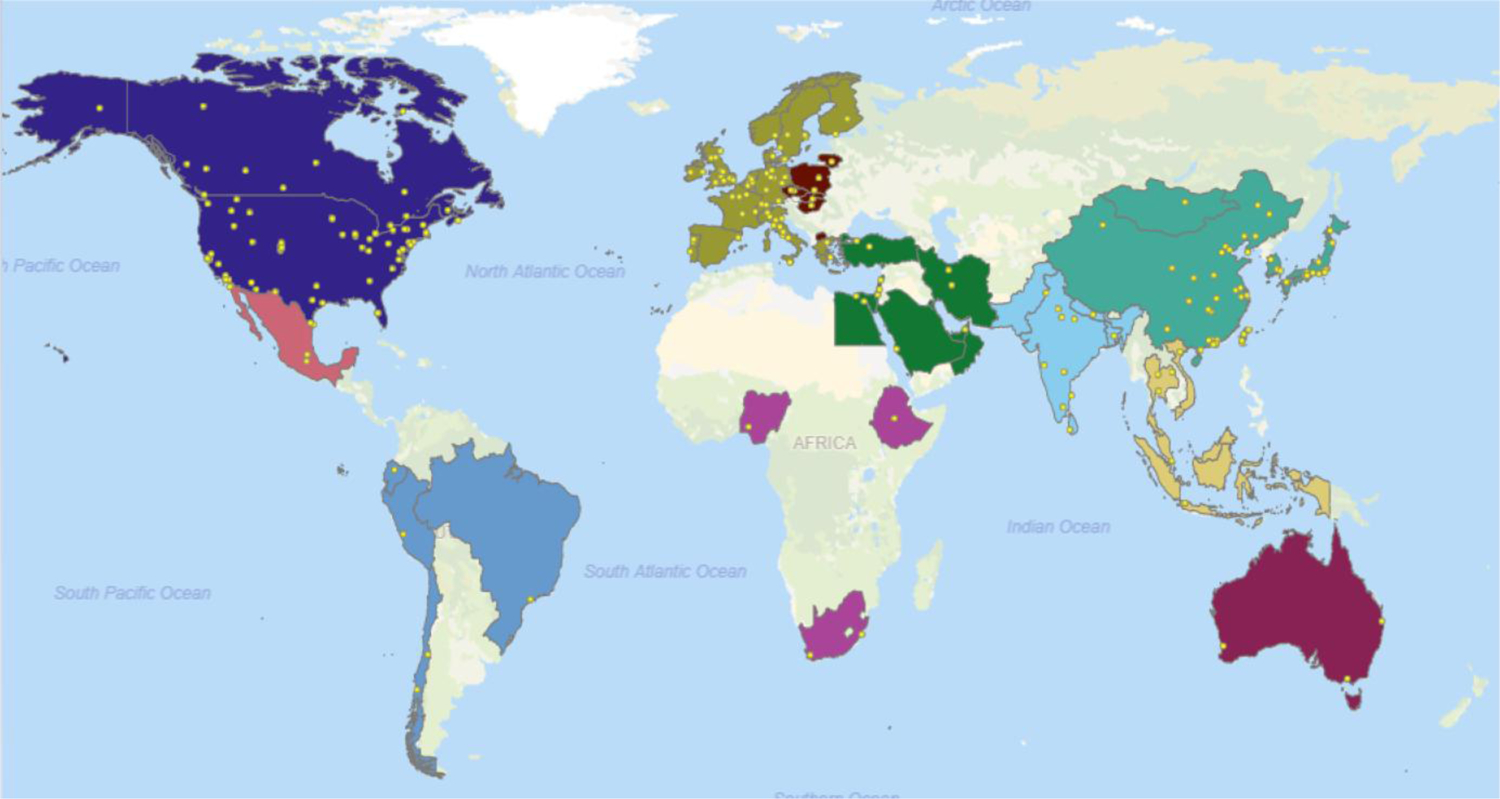
Location of sampling sites for studies included in the survey (1990–2019). Country colors identify the world region groupings used in the analyses. A few sampling locations could not be accurately mapped. Some nationwide studies are marked in the country capital.

Most of the studies focused on North America, East Asia, and Western Europe throughout the decades (Figure 4), while vast regions of the world are not represented at all in this survey, despite large populations. This may be either because indoor air quality investigations were not performed there, or because their results were not published in the peer-reviewed literature or indexed with at least an abstract in English. The United States was the most intensely studied country, with 27% of the studies and 29% of the publications; followed by China, with 17% of studies and 16% of publications; and Canada, with about 5% of each. Yet, already at this world-level perspective (Figure 4), it is apparent that even the most highly sampled regions of the world have actually been sampled quite sparsely.

### 3.2 | Sampling approaches

Collectively, the research projects in this survey sampled more than 21 200 homes, adding up to 4.91 million hours of sampling time across those homes. Individual studies varied in breadth of sampling from a single home (in about 14% of the studies) to 755,^59,60^ with a median number of 16 homes. The median duration of a study sampling campaign was 13 weeks. Samples were often collected repeatedly, ranging from repeated measures within a single day to 3 years^61^; the median time between first and last home visit was 5 days. The sampling time for reported measurement statistics also varied substantially, from 5 s (basically real-time monitoring) to 3 months of continuous monitoring,^62^ but the median was 24 h. Even the largest studies apportioned their resources to either sampling numerous homes or collecting samples of long duration, but rarely both. Logistical and funding limitations likely prevented the collection of long-term samples in a large number of homes.

A variety of building types and residential arrangements were sampled by the different studies, with multi-unit buildings (of unspecified height) most reported (14% of the studies), followed by single-family homes (11%), although, in more than 60% of studies, the type of home remained unspecified, or results were only reported for mixed types. Similarly, within a home, 36% of studies did not specify where air samples were collected. Among studies that reported sampling location(s), living rooms (or equivalent room where most time was spent), bedrooms, and kitchens accounted for 78%, 23%, and 17% of the sampling locations, respectively. Less than 10% of studies reported sampling multiple rooms.

### 3.3 | Indoor PM concentrations

#### 3.3.1 | Descriptive statistics

**Figure 5.**
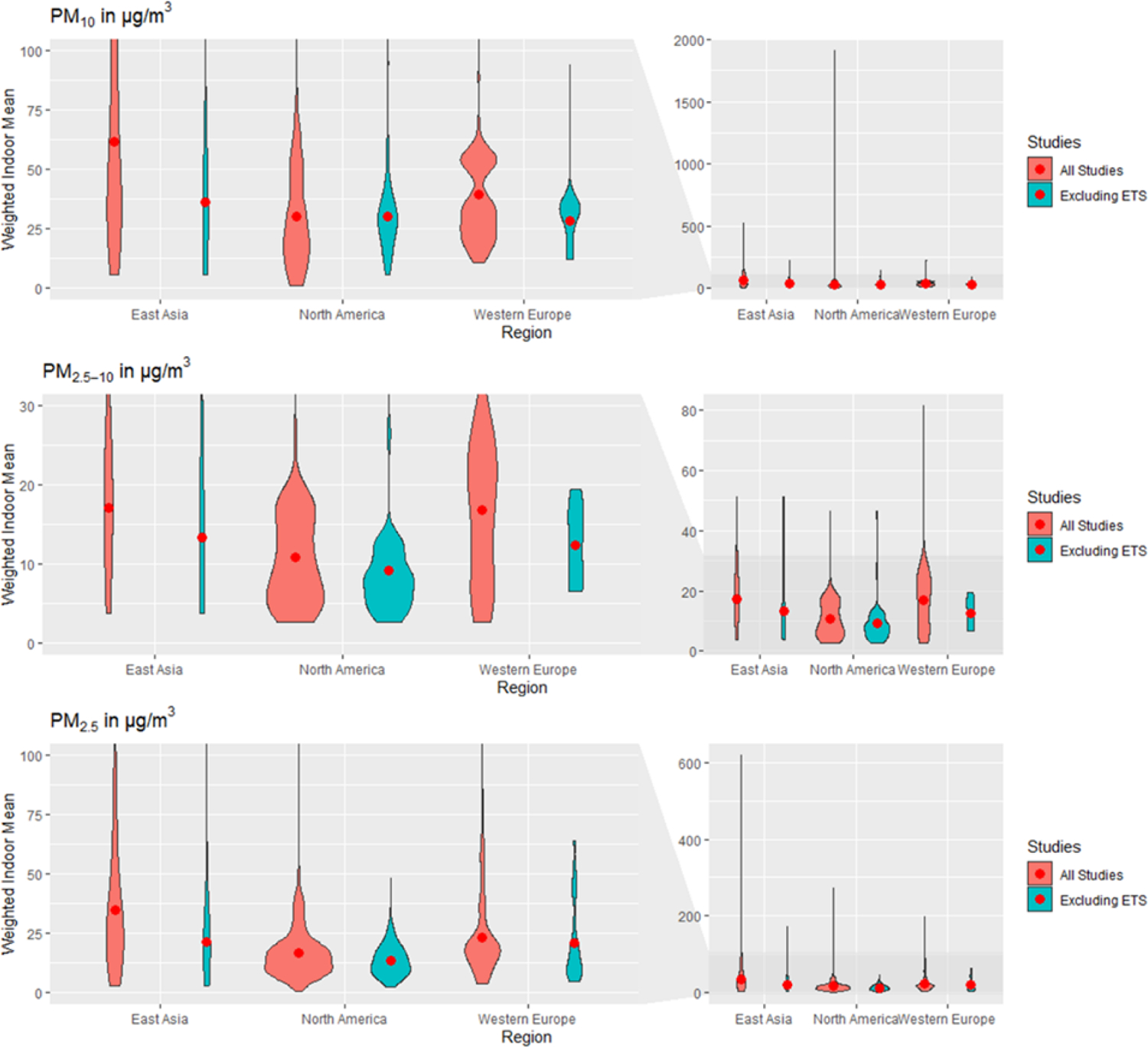
Weighted frequency distributions of means for PM_10_, PM_2.5–10_ and PM_2.5_ for East Asia, North America and Western Europe. Width is proportional to the number of means. The red points are displaying the weighted indoor mean of means. Studies labeled ‘Excluded ETS’ are restricted to mean measurements in homes without environmental tobacco smoke. Left panels show magnified region of right panels.

**Figure 6.**
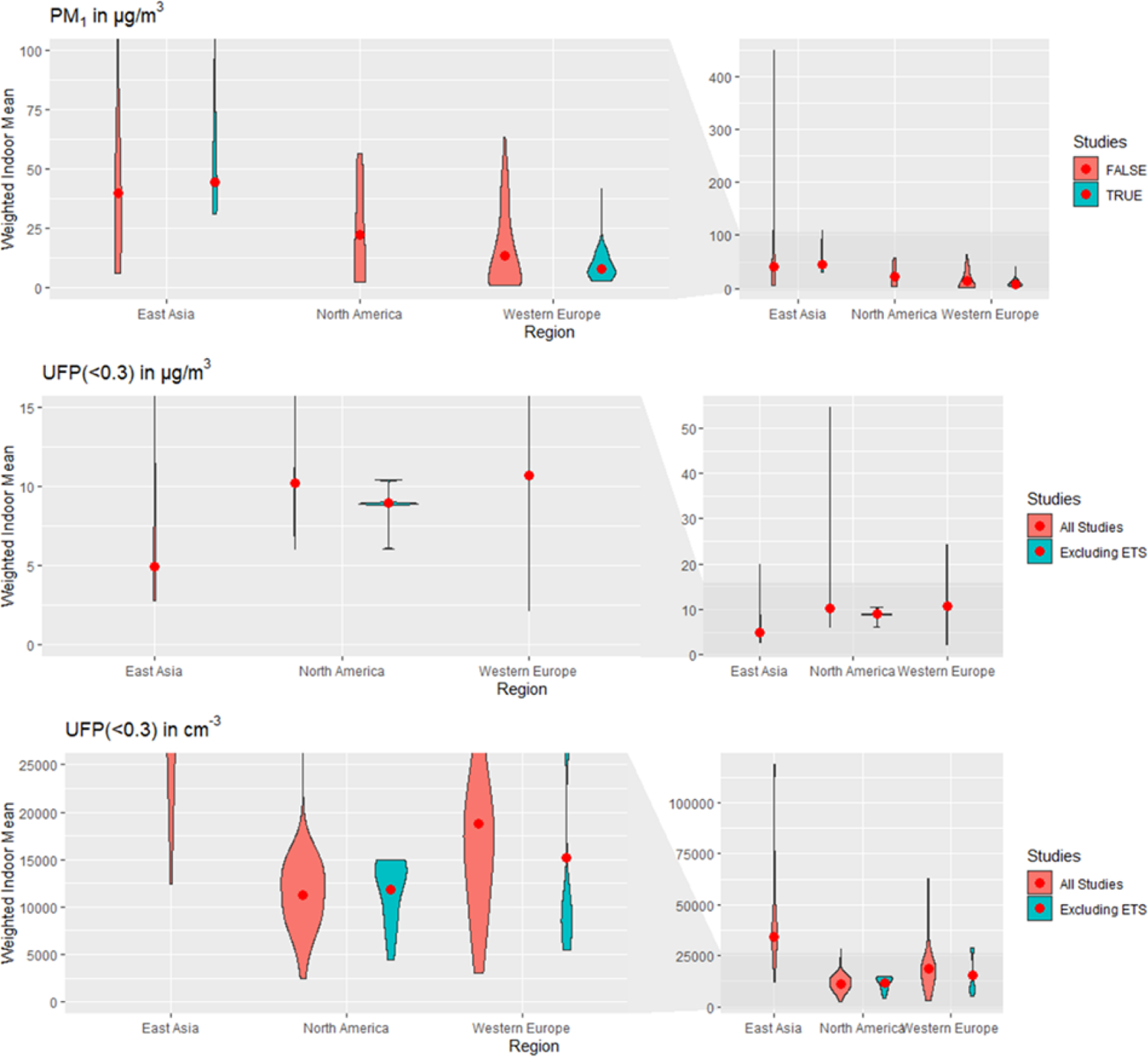
Weighted frequency distributions of means for PM_1_ mass (μg/m^3^),, UFP UFP(smaller than 0.3 μm) in both mass (μg/m^3^) and number (cm^–3^) for East Asia, North America and Western Europe. The red points are displaying the weighted indoor mean of means. Studies labeled ‘Excluded ETS’ are restricted to mean measurements in homes without environmental tobacco smoke. Left panels show magnified region of right panels.

Frequency distributions of weighted mean PM concentrations for the three regions with most data (Figures 5 and 6; Figures S2 to S5 show all other regions) vary by up to three orders of magnitude and are right-skewed. These general patterns similarly apply to unweighted means, which are even more widely distributed and skewed toward higher values (distributions not shown). Modal sections of the distributions across regions cluster around similar values for North America, Western Europe, and, based on more limited data, also Eastern Europe, at 5–10 μg/m^3^ for PM_2.5–10_ (coarse particles); 5–30 μg/m^3^ for PM_2.5_; 5000–20 000 cm^−3^ for UFP (number); and, less sharply, 0–25 μg/m^3^ for PM_1_. Distributions of PM_10_ are bimodal in North America and Western Europe, clustering around 15–25 and 50–60 μg/m^3^. This signifies that most homes are relatively similar across these regions in terms of indoor PM, and regions mostly differ on the frequency of extreme values. Size distributions for East Asia are generally more spread out than for other regions, with a less clearly identifiable modal section, and generally higher values (except for PM_2.5−10_). This pattern can reflect a great heterogeneity between sampling locations, drastic changes in concentrations over the time span considered, or both. To a degree, but with much less available data, a similarly spread-out distribution pattern can be seen for the larger size fractions (PM_10_, PM_2.5_, and PM_2.5–10_) in South Asia, and less clearly for West Asia and North Africa. The highest weighted mean concentrations are also occurring in these two regions. Much less data are available about other regions to identify consistent patterns, demonstrating again the scarcity of measurements for some regions in this survey.

To meet the needs of exposure and risk assessments in particular, quantiles of the weighted mean distributions were tabulated along with the number of studies synthesized by each group, and the total number of homes and observation times they collectively gathered (Tables 2–7). Although some of these sampling numbers may seemingly add up to impressive statistical power (up to thousands of homes and hundreds of thousands of hours of observation), two key limitations should be considered when using these concentration data. First, these observations were not generally drawn from truly random samples of indoor residences or times (as discussed above). Second, they can represent multiple decades of observations of indoor concentrations, which may have been changing over time. Tables 8 through 11 provide the first and last year of observations, which are described in greater detail in Section 3.3.2. To fully characterize the range of PM exposure and risk, such as worst-case scenario analysis, between-study statistics on maximum concentrations were provided as well (Tables 2–7). Generally, the maximum of the distribution of weighted means (i.e., the highest of mean values reported across studies) is comparable to the median of maximum values (selected among the maximum values provided by individual studies). Studies reporting means that far exceed the median of maxima could be reflecting special circumstances during sampling, such as wildfires, dust storms, or short-term measurements during cooking. Statistics on study minima are generally reduced to the trivial values of 0 (or the limits of detection) and are not presented.

It is tempting to compare weighted mean values of indoor PM concentrations to regulatory limits set for PM in ambient air, which are more often available than for indoor air. However, such comparisons are generally inappropriate and can be misleading. This is primarily because different national or international standards may be set weighing economic or practical considerations, in addition to evidence of health effects. Even when entirely health-based, the evidence on which they are built does not necessarily account for the reduction in exposure to ambient PM provided by buildings, nor for the variability of exposure to indoor-generated particles. Consequently, indoor exposures at levels below outdoor limits should not automatically be regarded as safe. One international set of health-based guidelines specifically developed to be applicable to indoor environments are the World Health Organization’s AQG levels (2021 update),^63^ recommending PM_10_ levels below 15 μg/m^3^ (annual) and 45 μg/m^3^ (daily); and PM_2.5_ levels below 5 μg/m^3^ (annual) and 15 μg/m^3^ (daily). In this analysis, all regions (Table 2) had weighted PM_10_ means well above 15 μg/m^3^, and several also above 45 μg/m^3^, ranging from 30 μg/m^3^ for North America to more than 200 μg/m^3^ for South Asia. Similarly, all regions (Table 4) registered weighted PM_2.5_ means well in excess of 5 μg/m^3^, and all regions except Oceania also above the daily 15 μg/m^3^ level, from 16.8 μg/m^3^ for North America to 162 μg/m^3^ for South Asia. As already remarked, the studies in this survey are not population representative, but if mean (and median) values generally exceed these guidelines, it would be reasonable to expect that, over the past decades, an important fraction of the population has been exposed indoors to levels of PM that are of concern for health, even in the absence of solid fuels or ETS.

#### 3.3.2 | Long-term trends

**Figure 7.**
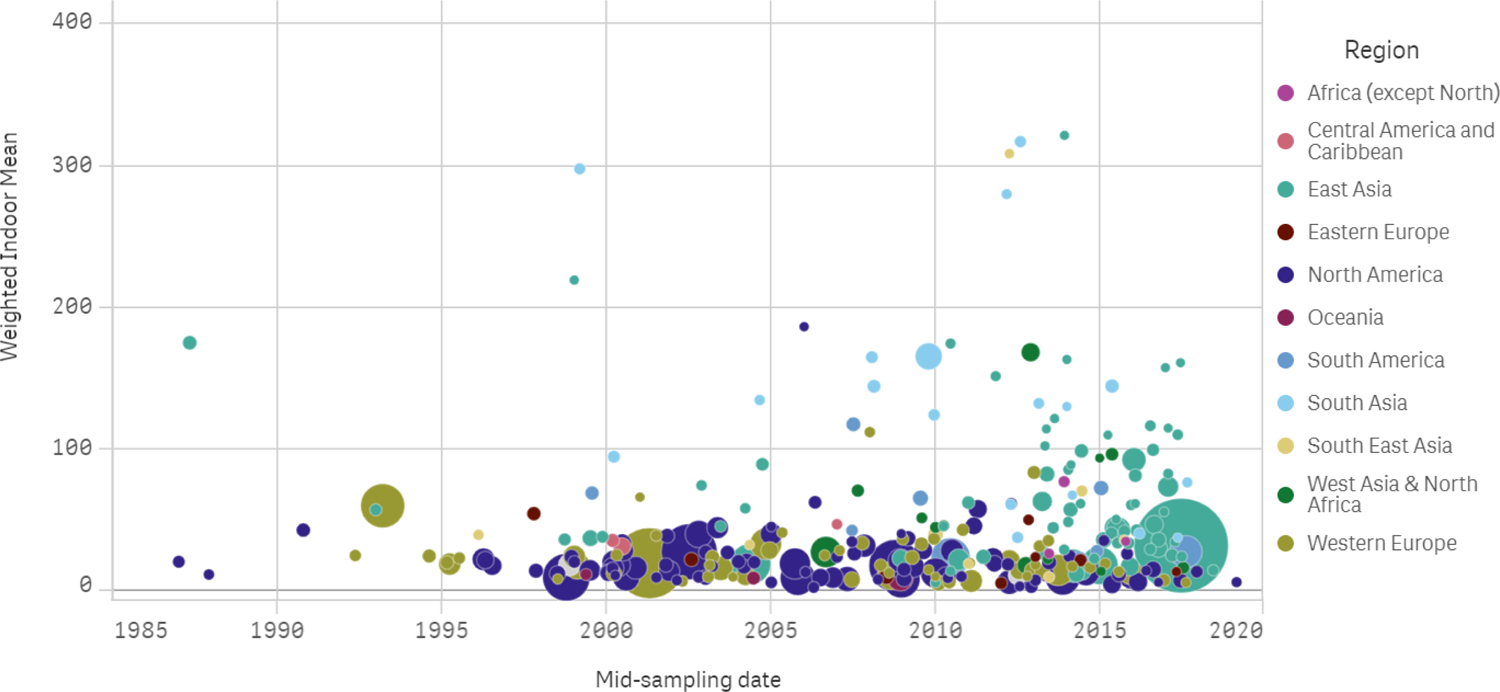
Weighted study means of PM_2.5_ (μg/m^3^) by mid-point of sampling campaign. Each marker shows the summary results of one research project, with diameter proportional to the study size (number of homes × sampling time), and color-coded by world region.

Indoor PM concentrations have been measured over the years through ad hoc studies (Figure 7), so that real-time series for specific locations are not available and time trends estimates inevitably confound some spatial variability within a region, country, or city. Yet, where results are consistent at different scales, they may still reveal real-time trends. To understand if and how indoor PM concentrations have changed over the decades of the survey, we can consider the regression results in Tables 8–11. Where few studies were available, regression parameters are of limited inferential value, because of their greater uncertainty, indicated as standard error of the estimate, and significance against the hypothesis that their value is zero. In such cases, the most informative elements reported in the table are the earliest and latest estimates, interpolations of the available data at both ends of the time span with reported measurements, suggesting a relative increase or decrease. Even these suggestive indications, for locations with fewer studies, need to be interpreted with caution, because they are more sensitive to the variability of individual study designs, including seasonality and type of sites. No generally applicable conclusion can be drawn about the time course of indoor concentrations, but a few regional and local trends stand out. Concentrations of indoor PM_2.5_ in North America, and most clearly in the United States, have been decreasing at a rate of about −0.3 to −0.5 μg/m^3^ per year. Concentrations of PM_10_ have been trending down as well, at a rate of −1.0 ± 0.4 μg/m^3^ per year. This pattern (for PM_2.5_) is also clearly seen more locally, in several US cities with sufficient available data. For the smaller size fractions, UFP number concentrations have shown a small increase from the earliest measurements in 2005, though it is significant only for Canada.

For East Asian locations, evidence of trends is mixed and more limited. While PM_2.5_ concentrations show statistically significant decreasing patterns for China, Taiwan, Beijing, and marginally significant for Taipei, no trends are significant for other individual East Asian cities, or for Japan, which can reflect a real lack of trend, lack of data, or both. Concentrations for Hong Kong, a city with relatively abundant data, are decidedly flat over time. South Korean measurements show a significant increase in PM_2.5_ and a marginally significant increase in PM_10_ (from a larger set of studies). The decreases for China and Beijing, however, strongly reflect the high concentrations in the early (1987) measurements of a single smaller study.^64^ No trends are significant for other size fractions in the region, except for a PM_10_ decrease in Taiwan, at a rate of −3.5 ± 1.5 μg/m^3^ per year.

Countries and cities in Western and Eastern Europe also display generally decreasing trends of indoor PM_2.5_ concentrations, but significantly so only for Finland, France, Greece, Italy, and the city of Athens. For the UK, the relatively numerous studies suggest no significant trend over time. The lack of significant trends elsewhere must be considered in light of the administrative fragmentation, leading to few studies per jurisdiction. The pooled regression for Western Europe (excluding Spain and Finland, whose residuals variances were too different from the rest) shows a highly significant decreasing trend of −0.94 ± 0.14 μg/m^3^ per year (*p*-value of coefficient: 3E–10). Trends for indoor PM_10_ and PM_1_ were mixed and not significant for any location, including for the pooled regressions. A marginally significant increase may be noted for UFP, like for North America.

Information on indoor concentrations in other regions was generally too sparse to produce informative results on their long-term changes, with few exceptions. Statistically significant decreases in PM_2.5_ concentrations can be observed for Chile (−5.6 ± 1.5 μg/m^3^ per year) and Bangladesh (−11 ± 1 μg/m^3^ per year), and a significant increase for Mexico (2.1 ± 0.9 μg/m^3^ per year), though the latter two estimates rely on only 2 and 3 studies, respectively (data not shown in the table). Comparatively, more numerous data were available for Singapore, India, and Pakistan, where no significant trends are observed. South Asia is the only region showing a significant decrease in PM_1_ concentrations, though this may simply reflect a tendency toward the mean from the very high levels reported in the earliest measurements.

Overall, results are consistent with generally stable indoor concentrations for the three decades of this survey, with specific regional exceptions for PM_2.5_ and PM_10_. Understanding all the factors contributing to these observed differences is beyond the scope of this survey, but these localized decreases may reflect well-documented declines in prevalence of smoking^65^ and ambient air concentrations of regulated pollutants.^66,67^ Even in a survey of this size, the limited availability of indoor PM measurements repeated in the same areas limits further exploration of these temporal trends.

### 3.4 | Factors affecting indoor concentrations

The importance of a long-term trend in explaining the observed variability in indoor PM concentrations varies for different locations but is generally low for locations with richer data sets (e.g., the United States, China, the UK, and Canada), as can be seen from the $R_{\text{d}}^{2}$ values in Tables 8–11, ranging as low as 0.00 to 0.16. The apparent influence of time trend on observed variability increases when few observations are available at a given location, but this is really only an indication of limited number of indoor PM measurements. Even when time trends are significant, however, the evolution of indoor PM concentrations must be traced to direct causes that also change over time. Here we explored outdoor PM concentrations and indoor ETS in some detail, although other factors are known to be important. Climate and seasonality, as well as type of site (e.g., urban, rural, and roadside), are expected to play a role and will be explored in future work. Ventilation rates and type (infiltration only, natural from doors and windows, mechanical, or a combination) also have a fundamental effect on indoor concentrations, based on physical considerations, but are not analyzed here, as only about 10% of the papers provided any information on air exchange rates, and even fewer (1.5%) on the type of ventilation associated with indoor PM measurements. In general, results reported were averaged over a range of ventilation rates and types. This is an important limitation of the literature on residential indoor PM. Human activities other than smoking are expected to be important as well, but outside the current scope of analysis.

#### 3.4.1 | Outdoor air concentrations

**Figure 8.**
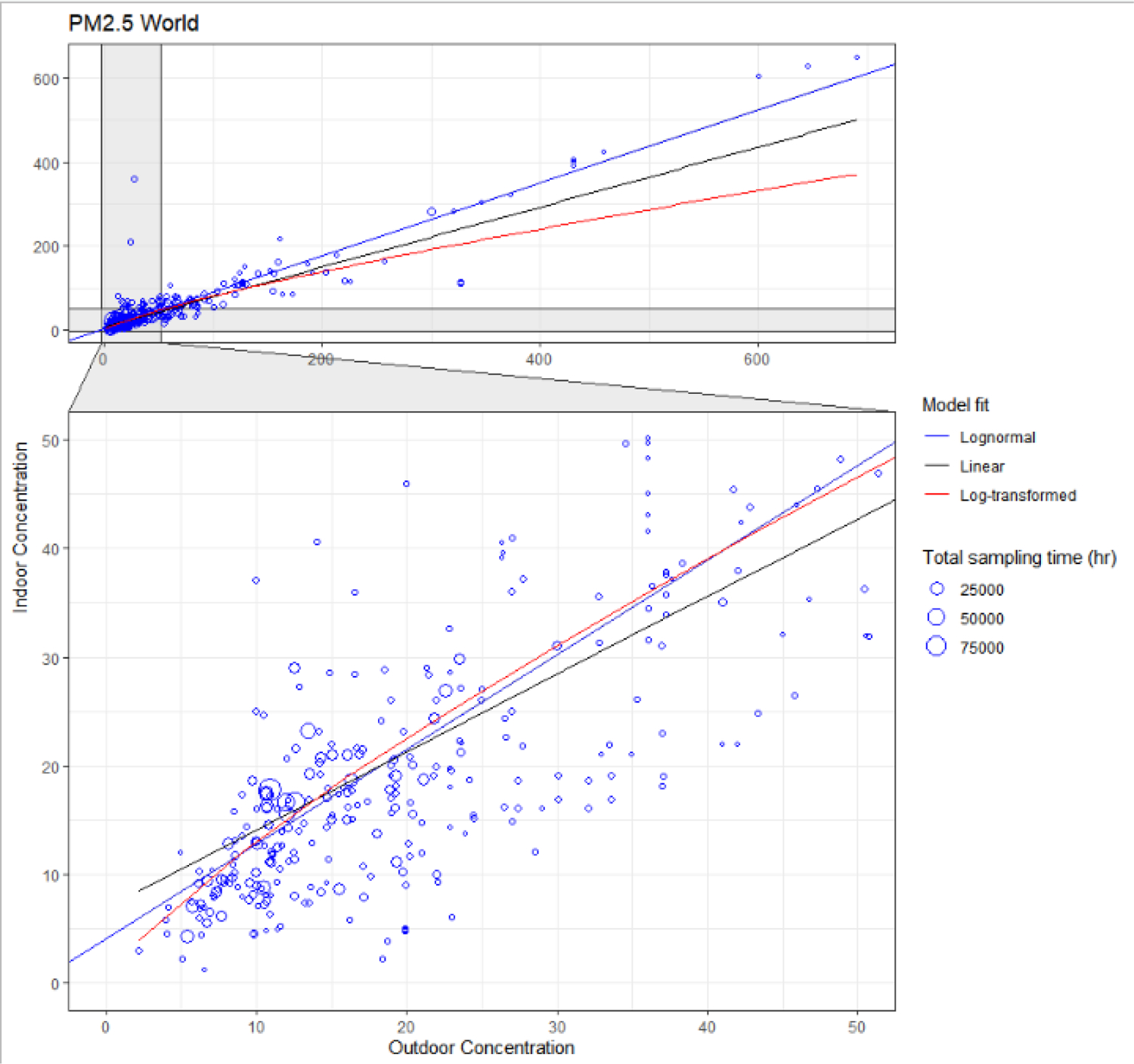
Scatterplot of mean indoor and outdoor (on site) concentrations pairs for PM_2.5_ (in μg/m^3^). Circle size indicates the total sampling time of the measurements (in hours) across all sampled buildings for a pair of mean concentrations in a study, which were used as weights in the regressions. The Model Fit lines show 3 different fitting approaches: lognormal regression, simple linear regression on unmodified data, and linear regression on log-transformed data. Bottom graph shows magnified region of top graph.

About half (198/428) of the studies in the survey reported paired indoor-outdoor (on site) measurements, and another 45 reported paired concentrations measured at off-site ambient monitors, usually regulatory monitors (referred to as “Ambient”). These pairs of indoor-outdoor concentrations were remarkably well correlated across studies, as can be seen in Figure 8 for PM_2.5_, demonstrating the important role of outdoor air in residential indoor air quality. The results of lognormal regressions on these paired concentrations, based on Equation (3), are reported in Tables 12 through 17.

Most regression models (and all those with larger data sets) show a significant effect of outdoor PM on indoor concentrations (β_1_ regression coefficient) at all size fractions. The magnitude and uncertainty of these coefficients, approximating the infiltration factor, vary for different size fractions and locations. The global model for PM_2.5,_ which has the most data, has outdoor air contributing 86% ±5% of its PM_2.5_ concentration to indoor air in homes, while the U.S. model has 79% ±13%, and East Asia 48% ±4%, for example. This factor varies, but not in a consistent pattern, when accounting for uncertainty, for the other size fractions, with 63% ±4% for PM_10_, 74% ±15% for PM_2.5 −10_, 55% ±4% for PM_1_, and 79% ±14% for UFP in the global models. The intercept terms, approximating indoor source contributions, are less often significant due to greater uncertainty, with similar values of PM_2.5_ (2 to 11 μg/m^3^) in the global models, North America and Western Europe, and higher values in East Asia. There was more similarity in intercept terms for PM_10_ (except for lower values in North America) and PM_2.5–10_. Due to the lower number of measurements, only global regressions were performed for PM_1_ and UFP.

Some coefficients β_1_ for outdoor air are greater than 1.0, which, if interpreted as infiltration factors, do not have a physical meaning in a mechanistic model. In this statistical model, such values indicate that outdoor air pollution has a positive correlation with some indoor sources. To remain within the variables of the model, this is often the case for ETS: spatially, as both ambient air pollution and smoking are higher in some locations and both low in others, and temporally where ambient air pollution declined over the survey period similarly to smoking prevalence.^65,66,68^ But other correlations may exist, reflecting excess burden of ambient air pollution on poorer communities that can also least afford the energy and material costs of filtration, ventilation, and other measures to reduce exposure to indoor sources.^69–72^ This effect (multicollinearity) also makes the regressions underestimate the intercept, interpreted as indoor-generated PM. The issue is especially notable for several models for North America (Tables 12–14), where both the simultaneous decline of outdoor PM and smoking prevalence,^65,66,68^ and disparities in exposure by socioeconomic status^69^ are well documented, but it is likely affecting other estimates. Estimates using only measurements in ETS-free homes partially correct for this effect and generally show lower coefficients, for the smaller size fractions, but not for PM_10_ and PM_2.5–10_. This effect also explains why our estimates of infiltration factors for PM_2.5_ are in some cases higher than those from measurements and modeling in the literature. For example, Fazli & Stephens^73^ calculated values of 0.4–0.5, and compiled values of 0.45–0.75 from the literature; MESA Air^19^ had values for different communities from 0.47 ± 0.15 to 0.82 ± 0.14, and EXPOLIS^8^ a range of 0.59 ± 0.17 to 0.70 ± 0.12 for different European cities. Estimates of infiltration factors for UFP (0.79–0.86) in the global models are also higher than may be expected from physical considerations (i.e., smaller than for PM_2.5_) and reported values of 0.1 to 0.4.^73,74^ Regression results from East Asia (0.31–0.48), where declines in ambient air pollution and smoking prevalence^68,75^ have been smaller^76,77^ and less correlated, are more in line with literature estimates.

The coefficients of the regression models, despite the limitations above, can be used to estimate the fraction of indoor PM that is generated from indoor sources vs. the fraction infiltrated from outdoors. A detailed exploration of these relative contributions is beyond the scope of this summary, but an example can help to better understand the implications of the results in Tables 12 to 17. The breakdown of sources for PM_2.5_ in the United States is particularly interesting as it can be compared to other estimates. From Table 12, the regression coefficients can be used to approximate *F*_inf_ = 0.788 ± 0.128 and indoor source contribution: 6.99 ± 1.62 μg/m^3^. At the mean level of outdoor concentration for the data set, 13.14 μg/m^3^, the contribution of outdoor PM_2.5_ is 10.35 ± 1.68 μg/m^3^, so the proportion of indoor-generated PM_2.5_ is 6.99/(6.99 + 10.35) = 40.3 ± 10.8%. Azimi & Stephens^78^ used data from both RIOPA and MESA Air studies to calculate the fractions of *total* PM_2.5_ exposures taking place in different microenvironments. Exposure in residences was about 70% of total exposure, 42% ±24% (of total) from outdoor-generated particles and 28% ±26% from those generated indoors, so that the fraction of indoor-generated to total residential particle exposure is 40% ±42% (using error propagation) for this microenvironment. It is important to note the wide uncertainties around both estimates, despite the coincidental exact match of results and, given the above limitations from multicollinearity, we must consider ours as a lower-bound estimate of the indoor-generated fraction.

Regression models using outdoor concentrations from off-site ambient monitors (Table 13) show lower coefficients for *F*_inf_ of PM_2.5_ in the global models, compared to those using outdoor concentrations measured just outside homes (Table 12), and account for a lower fraction of explained variance, as may be expected from the spatial misalignment introduced by these measurements. Results for regional and country models are more uncertain and do not show consistent patterns compared to corresponding models using on-site outdoor concentrations.

**Figure 9.**
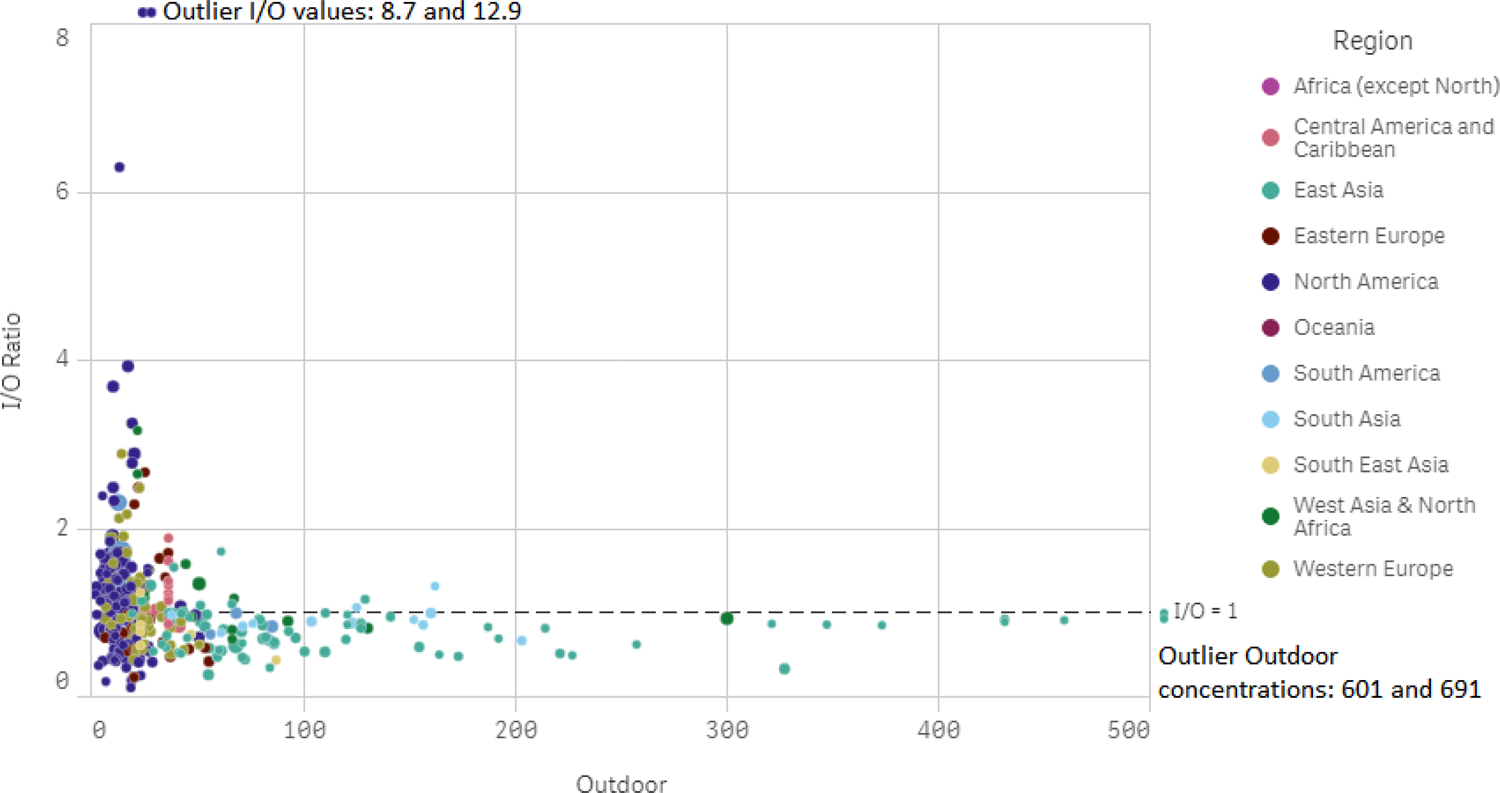
Indoor/outdoor ratios of PM_2.5_ with respect to outdoor (on site) concentrations (μg/m^3^). The values of four points outside the grid are shown next to them.

In the weighted regression models, infiltration of outdoor air and ETS alone accounted for a fraction ranging from 25% of the observed PM_2.5_ variability for Finland, to 96% for Taiwan (limiting the range to $R_{\text{d}}^{2}$ with distortions of <5% in Table 12). Infiltration alone explained almost 55% of the variability (with minimal overestimation) in the global model of samples taken in the absence of ETS. As outdoor PM concentrations decrease, infiltration of PM from outdoors accounts for a lesser and lesser share of indoor PM. This pattern can also be seen in Figure 9, where above about 50 μg/m^3^ of outdoor PM_2.5_, indoor/outdoor ratios above 1 are rare, but become frequent at lower outdoor concentrations. For PM_10_ (Table 14), the fraction of variability accounted for by infiltration and ETS is comparable to or higher than for the corresponding PM_2.5_ models, and even higher for ETS-free homes, suggesting a similarly important role for outdoor sources for this size fraction. However, this pattern does not hold for PM_2.5–10,_ with infiltration and smoking explaining 47% of the variability, and infiltration alone 40%. The regional PM_2.5–10_ models for East Asia and North America differ starkly, with the former accounting for almost all the variance and the latter for only 9%. Models of smaller size fractions, PM_1_ mass and UFP number, account for 53% to 76% of the variability, although these measures are substantially negatively distorted and true values are likely higher.

**Figure 10.**
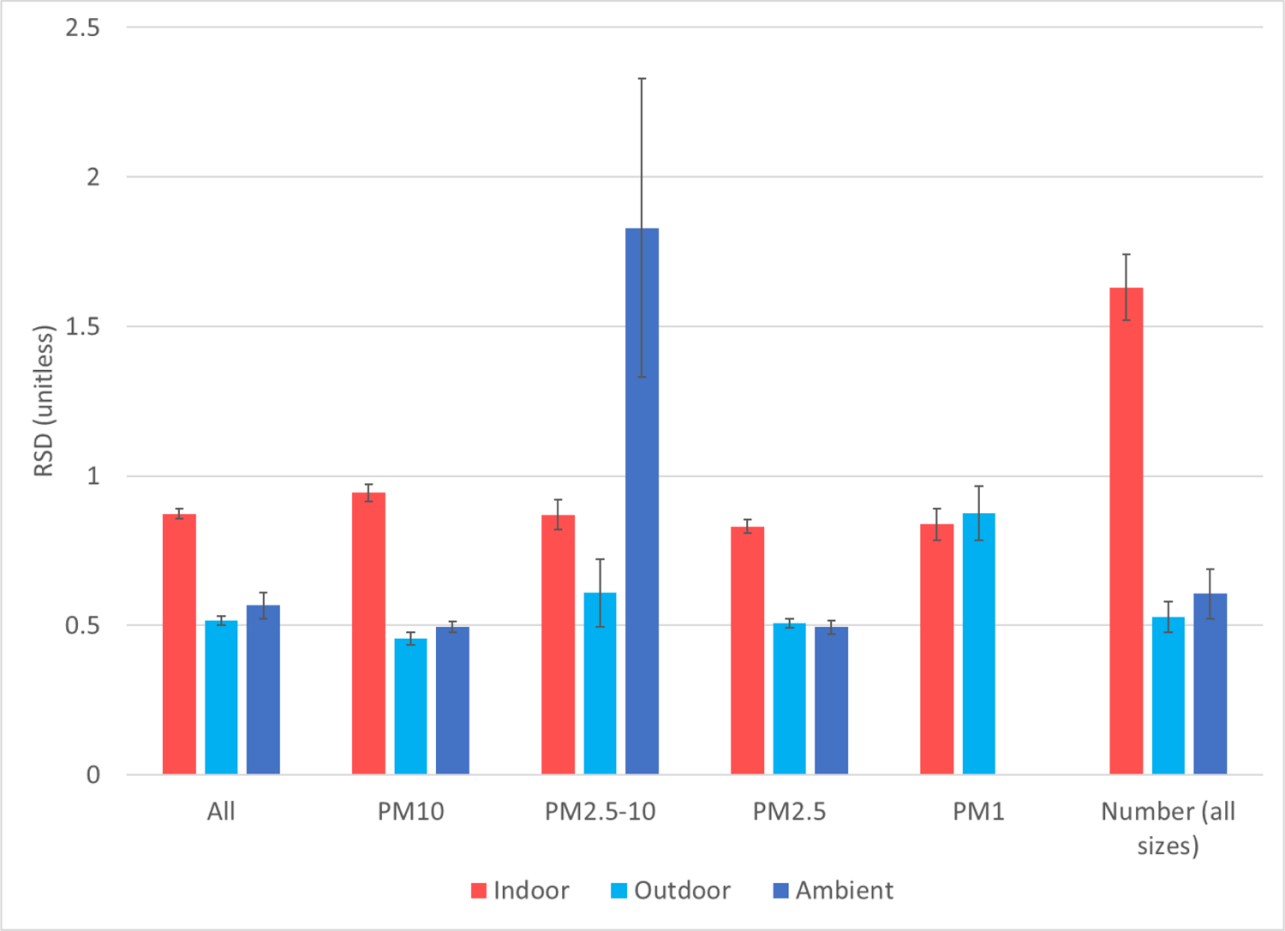
Variability of measurements Indoors, Outdoors (at the same location), and at off-site Ambient monitor, expressed as weighted mean of the within-study relative standard deviations (SD/Mean). Error bars are standard errors. Only means and SD reported in the studies are included, without calculated ones.

Although outdoor concentrations explain a good fraction of the variability of indoor concentrations between studies, this may not necessarily be the case within individual studies. Within-study variability explained by ambient air (*R*^2^) spans a wide range in larger multi-site studies, such as 0.06 to 0.44 (0.18 overall) for the RIOPA study,^2^ 0.40–0.83 (0.58 overall, calculated) for EXPOLIS,^8^ and 0.13 to 0.72 for RUPIOH^74^ (calculated). In general, the variability of indoor concentrations (over time or between homes) is larger than that observed outdoors, on site or at ambient air monitors. Within-study variabilities of mass concentrations, expressed as relative standard deviations, were approximately 50%–60% greater indoor than outdoors (Figure 10) for all particle sizes combined and for most size fractions, except for PM_1_, which had similar variability indoor and outdoor. There is no obvious physical reason why variabilities break the pattern for this size fraction, but its average indoor/outdoor concentration ratio was lower than 1, suggesting as a possible explanation that few situations with PM_1_ indoor sources were captured by the relevant studies. The variability of PM_2.5–10_ at off-site ambient monitors was larger than indoors or outdoors on site, and with greater uncertainty, but should not be overinterpreted as it is based only on six values from four studies. Indoor variability was even greater for number concentrations, more than twice that reported for outdoors samples. This larger within-study variability indoors reflects the greater complexity of indoor environments, which even when experiencing the same outdoor concentrations differ in air exchange and deposition rates, as well as the variety and intensity of indoor sources.

#### 3.4.2 | Environmental Tobacco Smoking (ETS)

About 42% of the studies reviewed provided at least some results from measurements in indoor environments free of ETS. These may have been the results of sampling designs that specifically excluded homes with ETS from participation, or in other cases a subset of the measurements limited to ETS-free homes. The remaining studies either did not mention ETS or did not provide separate data. As a result, it is not possible to estimate the full effect of ETS on indoor PM concentrations, but only to distinguish between ETS-free homes and homes with a mix of both smoking and non-smoking conditions, whose proportions vary from case to case. Despite this imperfect discriminator, estimates of PM from ETS-free residences can help us understand indoor air quality in the absence of an important PM source that is within an individual’s ability to control. Tables 2–6 show that the concentrations of PM of almost all sizes under ETS-free conditions were generally very similar to or lower than concentrations in the larger sets of studies. In a few cases, most noticeably for Eastern Europe and South Asia, concentration in ETS-free conditions were higher, but this can be explained by the small number of studies without ETS, mismatched with the larger collection in terms of locations and timing.

**Figure 11.**
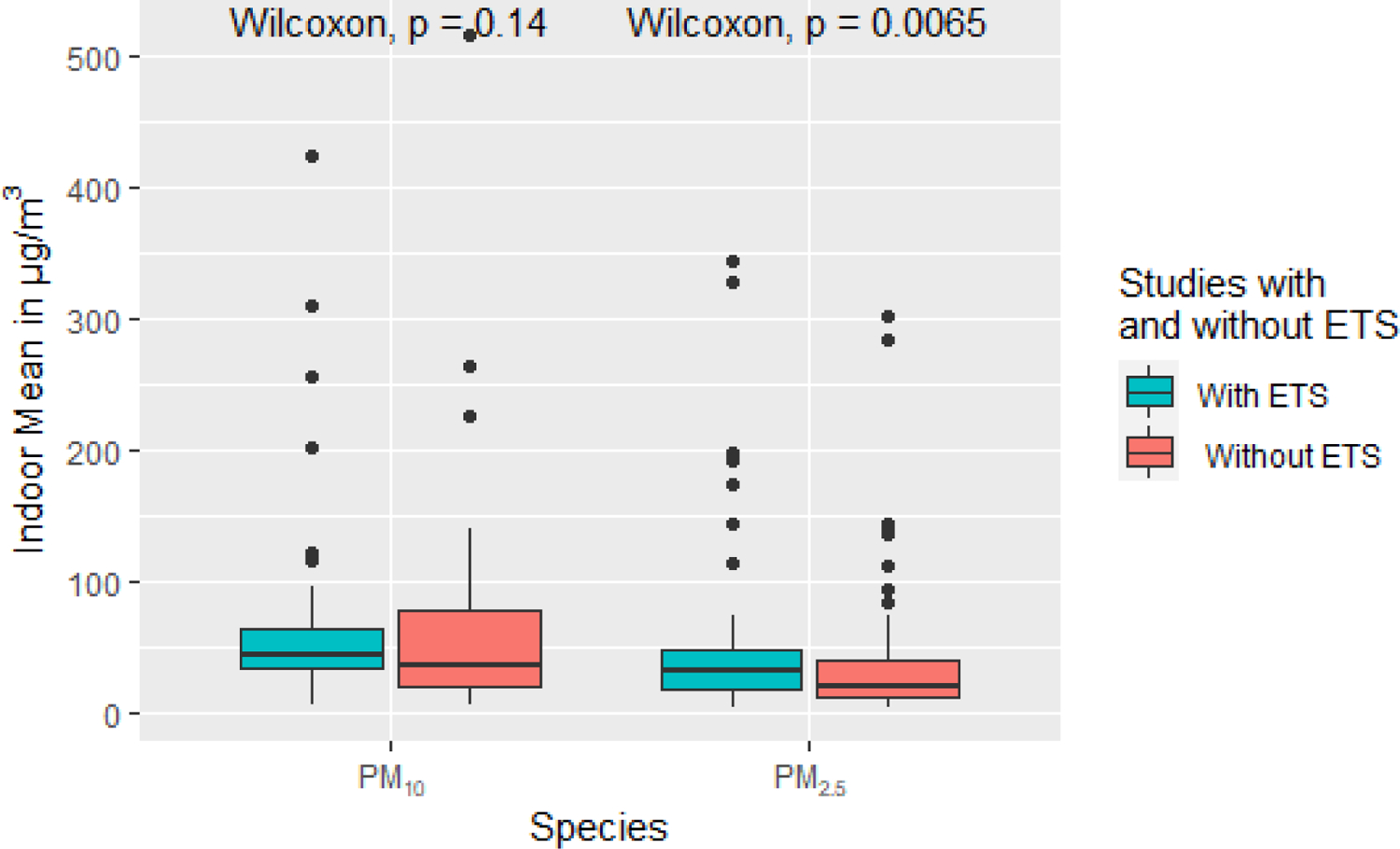
Box plot of indoor mean values for PM_10_ and PM_2.5_ for studies with and without ETS. Statistics labeled ‘Without ETS’ are restricted to measurements in homes without environmental tobacco smoke, while statistics labeled ‘With ETS’ contains measurements in homes with and without environmental tobacco smoke. Significance of Wilcoxon-Mann-Whitney test of differences between groups is shown. Additional descriptive statistics for these PM species as well as other size fractions can be found in the in the Supplementary Information (Table S3)

Similarly, pooled regression models assessing time trends (Tables 8 and 9) show significant differences of −3 μg/m^3^ (North America and Western Europe) to −13 μg/m^3^ (East Asia) for PM_2.5_, but no significant differences for PM_10_. Indoor to outdoor regression models (Tables 12–17) also show consistent reductions around −3 μg/m^3^ for North America and Western Europe and −8 μg/m^3^ for East Asia (although no reduction for China alone). PM_10_ indoor–outdoor regressions again do not show significant reductions from ETS-free conditions, except for East Asia (−13 μg/m^3^). Coarse PM and PM_1_ indoor–outdoor regressions have significant increases and decreases, respectively, for the global models, while the reduction for UFP is not significant. Finally, we examined just the subset of studies that reported results from both ETS-free and mixed ETS conditions (Figure 11; Table S3). Concentrations are significantly different for PM_2.5_ but not for PM_10_, based on the Wilcoxon–Mann–Whitney non-parametric test of difference between samples.

Overall, these results are consistent with the well-known impact of ETS on finer particles indoors, within the limitations of the crude separation of exposure conditions available.

#### 3.4.3 | Air cleaners

**Figure 12.**
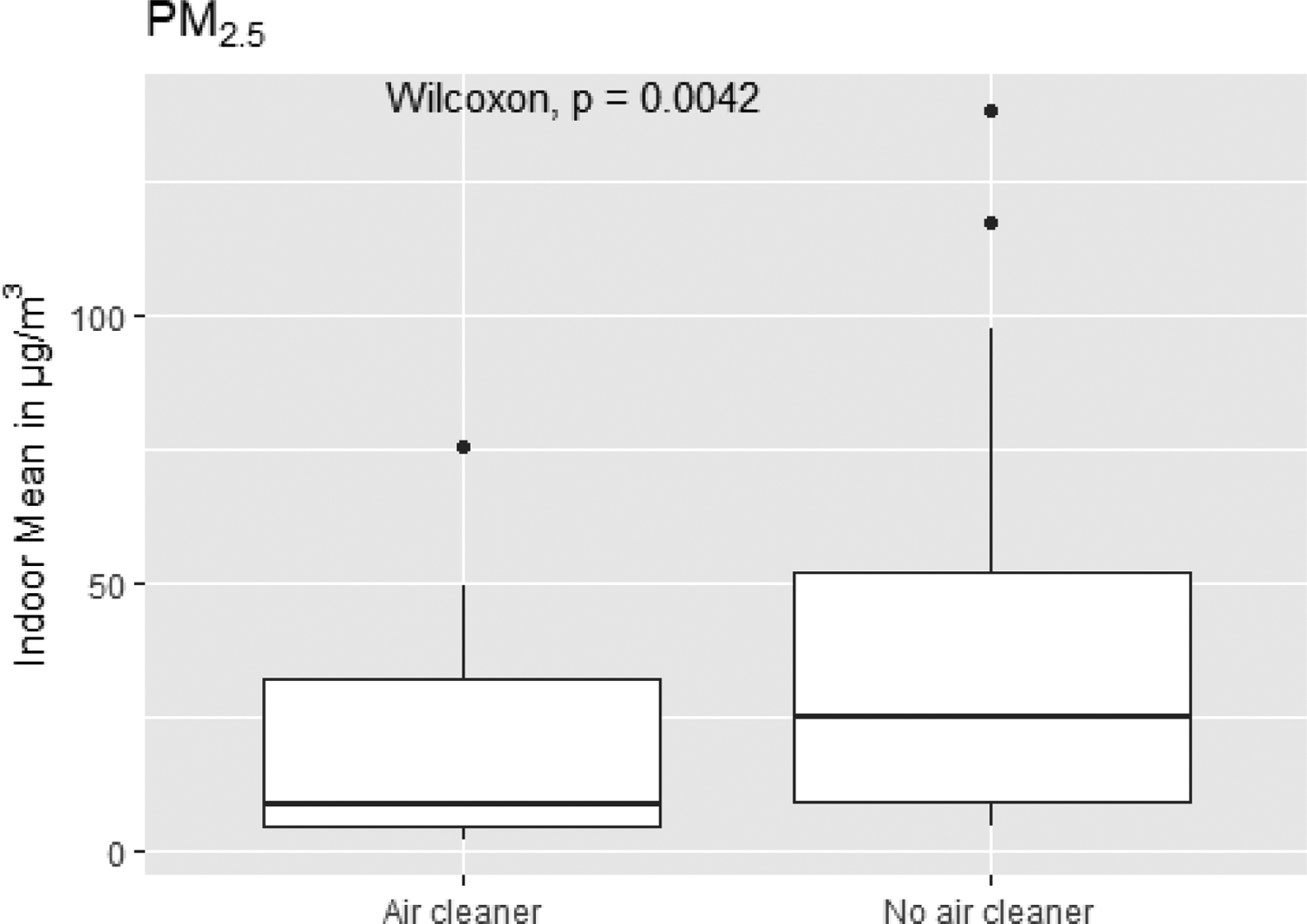
Box plot of indoor mean values for PM_2.5_ for studies reporting results both with and without air cleaners. Statistics labeled ‘No air cleaner’ are restricted to measurements in homes without air cleaners, yet the corresponding study contains measurements with air cleaners. Statistics labeled ‘Air cleaner’ contains measurements in homes with air cleaners. Significance of Wilcoxon-Mann-Whitney test of differences between groups is shown. Additional descriptive statistics for this PM species as well as other size fractions can be found in the in the supplementary Information (Table S4)

Only 31 studies (7%) reported on the use of air cleaners in homes during measurements, typically in intervention studies that provided the devices to study participants and prescribed their hours of operation. With one exception, all air cleaners worked through mechanical filtration. Because of the small number of results, only a few pooled regression models could estimate their effect (Tables 8 and 9). For PM_2.5_, reductions ranged from −4.5 ± 1.2 μg/m^3^ for North America, to −6.2 ± 2.2 μg/m^3^ for East Asia, and −6.8 ± 2.0 μg/m^3^ for Western Europe. All effects were significant. These values represent relative reductions from (weighted) mean indoor concentrations of −26.8% ±7.1%, −17.7% ±6.3%, and −29.4% ±8.7%, respectively, for North America, East Asia, and Western Europe. For PM_10_, the pooled model for East Asia estimated a significant reduction of −56.5 ± 24.5 μg/m^3^ or −91.6% ±39.7% of the weighted mean concentration. Limiting the comparison only to studies that reported measurement with and without air cleaners in use (Table S4), the difference of −11.6 μg/m^3^ is significant only for PM_2.5_ (Figure 12), based on the Wilcoxon–Mann–Whitney non-parametric test of difference between samples. Overall, these results provide evidence of meaningful reductions in indoor PM_2.5_ concentrations (and less clearly for PM_10_) during home use of air cleaners.

## 4 | CONCLUSIONS

This survey compiled more than 2000 sets of indoor PM measurements from hundreds of studies worldwide, representing sampling in more than 21 200 homes, for almost 5 million hours across those homes. Three world regions—North America, Western Europe, and East Asia—have been sampled more extensively and the common characteristics of their indoor environments, stemming from their mostly temperate climates, vastly urban and highly economically developed societies, inevitably shape our knowledge of indoor PM concentrations. Even within these more heavily studied countries, many geographic areas remain poorly characterized, especially those in less urban settings, which may potentially differ in terms of indoor sources and building characteristics.

Taken together, however, these measurements of indoor PM concentrations contribute to an improvement in our knowledge of residential PM exposures compared to estimates based primarily on ambient air pollution measurements or models, and behavioral survey data. Our results provide ranges of mean PM concentrations measured in indoor environments (e.g., 7.7–29.5 μg/m^3^ PM_2.5_ for 10th and 90th percentiles in North America; 20–112 μg/m^3^ PM_10_ for East Asia), as well as their variability. The majority of North American and European indoor environments studied were generally similar with respect to PM concentrations, clustering around the same relatively narrow ranges. Greater variability was observed for all regions of Asia, demonstrating a need for additional measurements, with careful consideration of sampling size in future studies conducted in these regions. In addition to supplying information for exposure and risk assessments, these systematic data could also be of value to product development for a growing list of consumer products related to indoor air quality.

The limited availability of health-based standards specific to indoor PM concentrations makes it difficult to evaluate the public health implications of the concentrations collected in this survey. While ambient standards and guidelines are not always appropriate benchmarks for indoor exposure risk, exposures to levels of concerns for public health are commonly occurring in many indoor environments. Concerns for even greater potential risks are raised when considering that the distributions of means for different studies we report represent a range of exposure concentrations extending, in part, beyond the values tracked. The maxima of the published values provide an indication of just how high indoor PM concentrations can become in real exposure scenarios, exceeding milligrams per cubic meter for PM_2.5_ and PM_10_ even in the absence of solid fuel combustion sources.

The indoor concentrations of PM of different size fractions were mostly stable over the survey period, with the notable exceptions of PM_2.5_ in Europe and most consistently in North America, which declined, at rates of 0.9 and 0.3 μg/m^3^/year, respectively. North American concentrations of PM_10_ also had significant declines, of about 1 μg/m^3^/year. The reasons for these declines may perhaps, in part, be attributed to declining tobacco use indoors, and decreasing ambient air concentrations of regulated PM size fractions.

Outdoor air concentrations of PM were consistently a major driver of indoor concentrations in all regions and for all size fractions. The infiltration factors calculated from regressions of paired concentrations were often higher than those published from mechanistic and modeling studies. This may possibly be explained by the correlation between ETS and ambient air pollution over time and by inequalities in the burden of air pollution exposures based on socioeconomic status. Homes free of ETS generally had significantly lower concentrations of PM_2.5,_ as expected. Another factor with large beneficial impacts on indoor PM_2.5_ was the use of filtration-based air cleaners.

Although our study offers the most expansive review of the available literature and data on indoor PM measurements and studies to date, some results are conspicuous for their absence. First, knowledge on indoor PM exposures for some of the world’s largest population centers rests on a handful of studies, population-representative studies are rare everywhere, while little or no data exist for some regions undergoing major economic and social transformations. Second, our understanding of the smaller PM size fractions indoors remains limited, as is the evidence about effective interventions within individual control. Third and perhaps most importantly, even where data do exist, any public health response must confront the lack of systematic and comprehensive records on indoor PM and its sources, as well as a challenging interpretation of measured levels in the near-absence of health-based standards intended for indoor exposures.

## Supplementary Material

Author manuscript Supplementary info

## Figures and Tables

**Table 1.** Search criteria.

Item	Criterion
Limits	1990 ≤ Publication Date ≤ 2019; Any language
1	Particulate(s) OR particulate matter OR ultrafine particle(s) OR PM10 OR PM2.5 OR fine particle(s)
2	Indoor* OR indoor air OR indoor environment OR IAQ OR IEQ
3	measure* OR concentration* OR characteriz*
4	residence* OR residential OR home* OR house* OR apartment* OR housing OR lodging* OR dwelling* OR condo*
5	biomass OR cookstove* (searched only in Abstract OR Title)
Combined search	(1 AND 2 AND 3 AND 4) NOT 5

A * indicates a wild character, capturing every ending of the word.

**Table 2.** Weighted statistics for means of PM_10_ and PM_1–10_ (μg/m^3^) measurements (including calculated means) and statistics for maximum of PM_10_ (μg/m^3^) measurements.

Region	Studies	Homes	Total Obs. Time (hr)	Statistics for Mean Measurements									Statistics for Maxima	
				N	Mean	SD	Wt. Mean	Min	Wt. 10%	Wt 50%	Wt 90%	Max	Median	Range
**All**	160	8228	894567	301	86.7	173	43.7	1.03	13.5	31.4	66.3	1910	115	2–3910
No ETS	62	1886	2.65E+05	98	59.4	72.4	43.1	5.6	17.9	34	56.2	518	102.8	20.2–985.8
Africa (except North)	3	791	18329	3	119	60.4	74.4	55	55	55	175	175	68.2	68.2–68.2
Central America and Caribbean	1	30	1752	3	44.3	13	51.5	31	31	57	57	57	97	60–154
No ETS	1	30	1.75E+03	3	44.3	13	51.5	31	31	57	57	57	97	60–154
East Asia	43	1676	123063	79	91.3	92.7	61.7	5.5	20.4	41	112	525	143.6	27.6–974.6
No ETS	17	264	7.39E+04	22	75.7	61.2	36.1	5.6	17.9	29.5	41	226	188	27.6–641
Eastern Europe	7	265	12819	14	39.5	21.4	32.9	15	20.8	28.1	66	79	75.15	31–202
No ETS	4	87	4.55E+03	7	50.6	23	49.2	22	22	38.8	79	79	52.42	38.4–90.3
North America	39	2258	290189	68	106	324	30.4	1.03	5	26.5	56.2	1910	105.6	2–3910
No ETS	18	573	6.30E+04	31	34.8	26.1	30.3	5.63	12.3	27.7	45	140	100.75	20.2–985.8
Oceania	3	142	15264	7	16.3	2.21	19.6	14	15.6	20.4	20.4	20.4	24.5	19–65
No ETS	2	118	1.51E+04	3	17	2.99	19.6	14.9	15.6	20.4	20.4	20.4		
South America	4	225	47688	4	87.6	65.1	42.4	35.2	38.3	38.3	38.3	173	194.85	19–208.2
No ETS	2	146	4.58E+04	2	71	46.3	41.3	38.3	38.3	38.3	38.3	104	194.85	181.5–208.2
South Asia	14	56	5.57E+03	25	219	100	235	61	143	231	311	503	329	101–1381.8
No ETS	2	5	7.08E+02	5	216	47.4	249	166	184	265	265	268.2	375	323–734
South East Asia	5	613	9.23E+03	7	40.6	25.6	37.5	14	14	31.4	64.1	71.6	105.3	105.3–105.3
West Asia & North Africa	9	901	1.56E+05	13	114	127	107	37.9	51.7	51.7	156	518	240	99–492
No ETS	3	210	1.26E+04	5	157	203	188	38.1	38.1	104	518	518	192.55	145–240.1
Western Europe	35	1271	2.15E+05	78	37.4	30.7	39.3	10.7	18.3	34	54	227	92.3	23–3598.4
No ETS	13	453	4.78E+04	20	26.7	18.4	28.4	12.4	12.4	34	34	94	42.25	40.6–374

Statistics labeled ‘No ETS’ are restricted to measurements in homes without Environmental Tobacco Smoke (ETS). Studies may report means for multiple locations and times (n is the total number of means). Not all studies provided a mean or a maximum. Data sources, organized geographically and by PM species, in Table S5.

**Table 3.** Weighted statistics for means of PM_2.5–10_ (μg/m^3^) measurements (including calculated means) and statistics for maximum of PM_2.5–10_ (μg/m3) measurements.

Region	Studies	Homes	Total Obs. Time (hr)	Statistics for Mean Measurements									Statistics for Maxima	
				n	Mean	SD	Wt. Mean	Min	Wt. 10%	Wt 50%	Wt 90%	Max	Median	Range
**All**	53	2773	4.54E+05	97	24.2	37.7	14.5	2.6	4.65	13.6	21.9	221	37.63	4–769.56
No ETS	28	1042	1.89E+05	51	24.4	37.8	13.2	2.62	4.64	12.8	19.4	202	45.65	14–335.4
East Asia	7	242	7.95E+04	10	20.5	15.4	17.1	3.8	5	20	20	51.2	36.25	33.3–39.2
No ETS	4	154	5.54E+04	7	21.2	18.8	13.4	3.8	5	13.6	34.1	51.2	33.3	33.3–33.3
Eastern Europe	3	54	5.78E+03	5	7.32	2.42	8.25	4.4	4.4	7.8	11	11	11.6	11.6–11.6
No ETS	1	1	2380	3	7.07	0.751	7.1	6.3	6.3	7.1	7.8	7.8		
North America	22	1307	1.73E+05	35	9.57	8.29	10.8	2.62	4.41	8.6	17.4	46.5	45.65	14–335.4
No ETS	12	631	7.32E+04	21	9.36	9.99	9.24	2.62	3.5	8.6	12.8	46.5	46.3	14–335.4
South America	3	207	4.75E+04	3	19.5	14.3	15.9	7.83	15.2	15.2	15.2	35.4	102.1	86.7–117.5
No ETS	2	148	4.61E+04	2	25.3	14.3	16.1	15.2	15.2	15.2	15.2	35.4	102.1	86.7–117.5
South Asia	5	18	2.48E+03	9	120	59.8	123	23.9	80.9	119	221	221	197	171–769.56
No ETS	2	5	7.08E+02	5	126	42.8	122	97.7	105	120	120	202	197	197–197
South East Asia	1	6	4.83E+02	1	27	NA	27	27	27	27	27	27		
West Asia & North Africa	3	646	1.20E+05	3	27.8	8.52	29.2	22.1	22.1	23.7	37.6	37.6		
No ETS	2	10	3.67E+03	3	27.5	8.73	29.3	22.1	22.1	22.1	37.6	37.6		
Western Europe	9	293	2.53E+04	31	16.9	18.4	16.9	2.6	5.5	17	26.7	81.4	19	4–88
No ETS	5	92.5	7.72E+03	10	11.4	5.37	12.4	6.58	7	13.1	17	19.5	9	31.2–31.2

Statistics labeled ‘No ETS’ are restricted to measurements in homes without Environmental Tobacco Smoke. Studies may report means for multiple locations and times (n is the total number of means). Not all studies provided a mean or a maximum. Data sources, organized geographically and by PM species, in Table S5.

**Table 4.** Weighted statistics for means of PM_2.5_ and PM_0.25–2.5_ (μg/m^3^) measurements (including calculated means) and statistics for maximum of PM_2.5_ (μg/m^3^) measurements.

Region	Studies	Homes	Total Obs. Time (hr)	Statistics for Mean Measurements									Statistics for Maxima	
				n	Mean	SD	Wt. Mean	Min	Wt. 10%	Wt 50%	Wt 90%	Max	Median	Range
**All**	326	16269	3155060	596	46.4	66.9	24.7	0.5	8.4	17.8	43	733	90.95	1–4641
No ETS	149	5810	971334	230	30.8	41.8	19.2	2.21	7.7	15	28.6	302	72	11.7–4378
Africa (except North)	3	59	762	4	42.8	22.9	63.0	25.8	25.8	76.5	76.5	76.5	126.05	34.1–218
Central America and Caribbean	4	76	9196	15	41.6	12.1	32.7	26	26	31	35.1	68.5	67	51.5–141.8
No ETS	1	34	6816	4	29.5	4.36	30.8	26	26	31	35	35	91.5	67–115
East Asia	81	2930	1074313	154	75.6	81	35.0	2.7	12.8	24.8	73	618	153	21–888.8
No ETS	33	821	199370	46	57.1	42.5	21.5	2.8	11.8	18.4	25.5	174	98.7	21–623
Eastern Europe	10	304	17632	18	23.5	15.7	21.4	4.9	9.3	15.7	53	55	74.35	8.3–203.2
No ETS	5	134	6809	8	28.7	16.8	32.6	11.4	15.7	25	55	55	60.62	31.1–96
North America	109	6518	1059622	204	20.2	24.7	16.8	0.5	7.71	16.2	29.5	272	78	1–2100
No ETS	61	2712	472509	98	14.5	8.78	13.6	2.21	7	12.8	20.9	48.2	65.23	11.7–508.2
Oceania	3	132	15998	6	9.46	1.58	8.44	7.99	8.4	8.4	8.67	12	15.3	15.3–18
No ETS	2	118	15312	3	8.35	0.34	8.42	7.99	8.4	8.4	8.67	8.67		
South America	10	606	119520	12	68.9	42.4	32.4	20	23.2	27.1	64.8	135	177.55	41–373.9
No ETS	3	150	46536	3	44.6	22.8	25.5	23.2	23.2	23.2	23.2	68.5	177.05	149.8–204.3
South Asia	19	452	44689	35	151	127	162	37.1	117	165	165	733	258.5	86–4378
No ETS	4	34	1837	8	134	103	194	40	40	285	302	302	196	112–4378
South East Asia	8	532	1493	14	48.2	76.6	41.5	5.8	19.8	39.2	70	308	1140	1040–1240
No ETS	1	5	325	2	8.65	4.03	9.22	5.8	5.8	11.5	11.5	11.5		
West Asia & North Africa	13	938	156376	15	64.4	67.6	58.6	13.2	18.6	26.9	108	283	74.15	52.7–4641
No ETS	5	224	13152	7	76.1	96.2	99.9	7.76	16	68.6	283	283	62.5	52.7–72.3
Western Europe	68	3722	655456	119	24	24.8	23.1	4	7.96	19	49	197	63.7	6–2120
No ETS	35	1578	208667	51	17.9	12.5	21.1	4.61	7	15.6	51	64	39.5	19–403

Statistics labeled ‘No ETS’ are restricted to measurements in homes without Environmental Tobacco Smoke. Studies may report means for multiple locations and times (n is the total number of means). Not all studies provided a mean or a maximum. Data sources, organized geographically and by PM species, in Table S5.

**Table 5.** Weighted statistics for means of PM_1_ and PM_0.1–1_(μg/m^3^) measurements (including calculated means) and statistics for maximum of PM_1_, and PM_0.1–1_(μg/m^3^) measurements.

Region	Studies	Homes	Total Obs. Time (hr)	Statistics for Mean Measurements									Statistics for Maxima	
				n	Mean	SD	Wt. Mean	Min	Wt. 10%	Wt 50%	Wt 90%	Max	Median	Range
**All**	43	1280	6.63E+04	77	52.3	74.5	23.5	0.9	1	10.4	51	449	73.5	8–862
No ETS	12	171	1.08E+04	20	31.6	30.6	19	3	6.9	10.4	48.2	110	38.165	18.7–181
Africa (except North)	1	15	4.18E+01	2	11.5	3.99	11.3	8.65	8.65	8.65	14.3	14.3		
East Asia	10	600	2.36E+03	16	101	135	40.1	6.1	25.9	33.4	48.2	449	141	129–153
No ETS	2	21	1.10E+03	3	63.1	41.5	44.6	31.2	31.2	48.2	48.2	110		
Eastern Europe	3	52	5.31E+03	5	11.8	6.64	10.8	5.7	7.3	7.3	22.7	22.7	34.33	18.7–51.9
No ETS	1	1	2.38E+03	3	15.3	6.38	15.6	11.6	11.6	11.7	22.7	22.7	34.33	18.7–51.9
North America	5	115	1.94E+04	7	21.9	21.9	22.2	2.55	5.2	8.3	47	56.5	110	66–862
No ETS	1	59	1.44E+03	1	23.6	NA	23.6	23.6	23.6	23.6	23.6	23.6		
South Asia	6	15	2.93E+03	16	90	42.9	106	31	99	103	135	157	76	34–260
No ETS	2	5	7.08E+02	5	56.8	27.4	87	34.9	35	101	101	101	76	66–181
West Asia & North Africa	2	5	4.90E+03	4	43.7	39.1	30.5	10.1	10.1	10.4	84.4	84.4	42	42–42
No ETS	1	1	3.46E+03	2	10.2	0.212	10.2	10.1	10.1	10.1	10.4	10.4	42	42–42
Western Europe	15	419	3.00E+04	26	20.7	17.3	13.4	0.9	0.9	8.1	37.9	63.4	75.7	8–601
No ETS	6	143	3.14E+03	7	13.3	13.1	7.78	3	3	6.9	14.7	41.6	16	22.4–22.4

Statistics labeled ‘No ETS’ are restricted to measurements in homes without Environmental Tobacco Smoke. Studies may report means for multiple locations and times (n is the total number of means). Not all studies provided a mean or a maximum. Data sources, organized geographically and by PM species, in Table S5.

**Table 6.** Weighted statistics for means of particle number - cutoffs unspecified (cm^–3^) measurements (including calculated means) and statistics for maximum of particle number - cutoffs unspecified (cm^–3^) measurements.

Region	Studies	Homes	Total Obs. Time (hr)	Statistics for Mean Measurements									Statistics for Maxima	
				n	Mean	SD	Wt. Mean	Min	Wt. 10%	Wt 50%	Wt 90%	Max	Median	Range
**All**	17	365	2.40E+04	26	15983	35708	14298	4.58	75.2	12083	20352	185211	103582.5	10.7–389076
No ETS	9	146	2.11E+04	14	5882	6218	12923	4.58	29.1	12083	20352	20352	17451	10.7–246000
East Asia	3	10	4.20E+02	4	50433	90182	62377	5.19	16400	16400	185211	185211	243.399	12.67–303247
No ETS	1	1	1.20E+01	2	60.3	77.9	60.3	5.19	5.19	60.3	115.34	115.34	128.03	12.67–243.40
North America	8	174	1.62E+04	10	5158	7202	13780	4.58	75.2	12083	20352	20352	203068.5	10.7–389076
No ETS	4	101	1.86E+04	7	7300	7743	13191	4.58	29.1	12083	20352	20352	10.7	10.7–10.7
Oceania	1	14	6.72E+02	1	10900	NA	10900	10900	10900	10900	10900	10900	22100	22100–22100
South East Asia	1	6	9.68E+02	1	18366.1	NA	18366	18366	18366	18366	18366	18366	103165	103165–103165
Western Europe	4	161	5.79E+03	10	13298	10483	15247	2250	3000	12715	26653	31816	104000	7510–246000
No ETS	4	44	2.55E+03	5	6225	3887	7019	2250	2250	7948	11815	11815	89200	58400–246000

Statistics labeled ‘No ETS’ are restricted to measurements in homes without Environmental Tobacco Smoke. Studies may report means for multiple locations and times (n is the total number of means). Not all studies provided a mean or a maximum. Data sources, organized geographically and by PM species, in Table S5.

**Table 7.** Weighted statistics for means of Ultra Fine Particles (UFP) (including calculated means) and statistics for maximum of UFP measurements..

PM species and unit	Studies	Homes	Total Obs. Time (hr)	Statistics for Mean Measurements									Statistics for Maxima	
				n	Mean	SD	Wt. Mean	Min	Wt. 10%	Wt 50%	Wt 90%	Max	Median	Range
UFP, PM_0.1_, PM_0.25_ and PM_0.5_ (μg/m^3^)	12	228	3.08E+04	31	18.2	17	14.1	2.2	2.8	8.99	37.6	68.2	136.5	69.86–143.9
UFP, PM_0.1_ and PM_0.25_ (μg/m^3^)	10	208	2.94E+04	19	15.1	15.5	10.4	2.2	2.2	8.93	19.8	54.5	103.18	69.86–136.5
UFP, PM_0.1_ and PM_0.25_ (cm^–3^)	31	1 074	6.92E+04	54	17154	18648	15608	2.44 E+03	7990	14000	26000	1.19 E+05	69640	4000–3.80 E+06

Studies may report means for multiple locations and times (n is the total number of means). Not all studies provided a mean or a maximum. Data sources, organized geographically and by PM species, in Table S5.

**Table 8.** Coefficients mean, standard error, and significance for log-normal weighted regression of mean concentrations of indoor PM_2.5_ (and PM_0.25–2.5_) over time: Indoor ~ Intercept + β_1_ * Weight *(Sampling midpoint – 1980).

Location	Intercept	Year since 1980 (β_1_)	Earliest estimate	Latest estimate	n of means	n of studies	R^2^_d_ dist.	Pooled models
**Canada**	25.6 ± 8.7 7.5E-3**	−0.55 ± 0.28 6.3E-2.	16.7 (1996)	5.2 (2016)	24	15	0.16 −0.5%	FK p-value = 0.1083 R^2^_d_ = 0.23, dist. = 5.0% Intercept: 21.6 ± 2.8; p = 7E-13*** Year: −0.35 ± 0.09; p = 3.0E-4*** No ETS: - 3.4 ± 0.9; p = 3.9E-4*** Air cleaners: - 4.5 ± 1.2; p= 4.0E-4*** USA: +4.3 ± 1.1; p= 6.6E-5***
**USA**	27.1 ± 3.1 3.2E-15***	−0.43 ± 0.11 1.0E-4***	24.1 (1987)	10.2 (2019)	180	87	0.08 0.9%	
*Baltimore*	124 ± 39 9.6E-3***	−3.7 ± 1.3 1.9E-2**	55.1 (1998)	12.9 (2010)	13	6	0.62 14%	
*Boston*	32.8 ± 6.0 8.4E-5***	−0.71 ± 0.19 2.4E-3**	21.3 (1996)	8.9 (2013)	17	11	0.54 2.8%	
*Detroit*	53.4 ± 13.3 1.0E-2*	−1.2 ± 0.43 3.9E-2*	28.9 (2000)	10.8 (2015)	8	5	0.53 0.9%	
*Los Angeles*	54.0 ± 12.8 1.8E-3**	−1.7 ± 0.50 5.5E-3**	20.8 (1998)	6.6 (2007)	13	4	0.64 3.4%	
*New York*	33.4 ± 7.0 3.1E-4***	−0.53 ± 0.24 4.3E-2*	23.3 (1999)	14.8 (2015)	17	8	0.23 −3.0%	
**China**	112 ± 32 6.6E-4***	−1.9 ± 0.88 3.1E-2*	98.1 (1987)	37.8 (2018)	118	55	0.06 0.8%	Pooled model also includes data from Japan n of means = 142; n of studies = 67 FK p-value =0.1016 R^2^_d_ = 0.53, dist. = −0.8% Intercept: 119 ± 31; p = 2.1E-4*** Year: −2.09 ± 0.86; p = 1.7E-2* No ETS: - 12.8 ± 4.0; p 1.9E-3** Air cleaners: - 6.2 ± 2.2; p 7.0E-3** Japan : −70.7 ± 35.7; p 4.9E-2* Taiwan: −72.1 ± 36.9; p 5.2E-2. Japan × Year: 1.4 ± 1.0 p = 0.18 Taiwan × Year: 1.6 ± 1.1 p = 0.13
**Taiwan**	64.2 ± 19.4 3.8E-3**	−1.4 ± 0.24 2.7E-2*	45.9 (1993)	14.6 (2015)	21	9	0.27 −1.2%	
**South Korea**	−149 ± 39.3 5.3E-3**	+5.2 ± 1.2 2.0E-3**	8.2 (2010)	40.0 (2016)	11	7	0.51 4.3%	
*Beijing*	212 ± 47 2.6E-4***	−4.8 ± 1.3 1.9E-3**	178 (1987)	33.2 (2017)	22	15	0.64 1.1%	
*Guangzhou*	−276 ± 244 0.30	+14.2 ± 10 0.21	57.5 (2003)	257 (2017)	9	4	0.40 1.9%	
*Hong Kong*	34.2 ± 17.3 6.4E-2.	−0.07 ± 0.53 0.89	32.7 (1998)	31.2 (2018)	21	9	0.00 3E-5	
*Seoul*	−122 ± 72 0.15	+4.5 ± 2.10 0.89	13.8 (2010)	38.9 (2016)	8	6	0.33 −0.2%	
*Shanghai*	187 ± 187 0.35	−4.4 ± 5.1 0.41	36.7 (2014)	21.9 (2017)	10	7	0.10 0.4%	
*Taipei*	60.4 ± 22.0 1.9E-2*	−1.3 ± 0.66 7.7E-2.	43.6 (1993)	15.7 (2014)	14	7	0.27 −1.5%	
*Tianjin*	1755 ± 1087 0.21	−45.4 ± 28.8 0.21	209 (2014)	43.0 (2017)	6	4	0.51 0.7%	
*Xi’an*	109 ± 96 0.37	−1.1 ± 2.6 0.73	74.7 (2011)	68.5 (2017)	5	4	0.05 3E-4	
**France**	76.7 ± 26.9 2.2E-2*	−2.0 ± 0.84 4.1E-2*	61.9 (1992)	13.0 (2014)	11	5	0.39 −1.0%	Pooled model also includes data from Belgium, Denmark, Germany, Ireland, Switzerland n of means = 109; n of studies = 63 FK p-value = 0.05062 R^2^_d_ = 0.47, dist. = 5.8% Intercept: 43.8 ± 3.8; p< 2E-16*** Year: −0.94 ± 0.14; p = 3.4E-10*** No ETS: - 3.0 ± 2.0; p 0.12 Air cleaners: - 6.8 ± 2.0; p 7.0E-4***
**Greece**	41.2.6 ± 6.7 1.7E-4***	−0.78 ± 0.20 4.5E-7***	26.7 (1998)	12.5 (2016)	12	7	0.65 −1.8%	
**Italy**	87.6 ± 5.9 1.2E-8***	−2.1 ± 0.21 4.5E-7***	61.9 (1992)	13.0 (2014)	14	7	0.87 −0.7%	
**Netherlands**	37.3 ± 7.9 4.2E-2*	−0.71 ± 0.26 0.11	26.4 (1995)	11.7 (2015)	5	4	0.67 −2.2%	
**Norway**	25.1 ± 11.4 0.16	−0.35 ± 0.37 0.44	19.9 (1994)	13.4 (2013)	5	4	0.27 −2.2%	
**Portugal**	21.0 ± 151 0.89	+0.73 ± 4.8 0.88	41.4 (2008)	45.9 (2014)	11	6	0.00 0.0%	
**Sweden**	−12.2 ± 51.2 0.83	+0.94 ± 2.1 0.70	9.6 (2003)	13.4 (2006)	5	4	0.06 −3.0%	
**UK**	27.8 ± 5.8 8.3E-5***	−0.35 ± 0.37 0.14	22.3 (1995)	14.3 (2017)	25	13	0.08 1.6%	
**Finland**	19.0 ± 2.9 1.2E-3**	−0.42 ± 0.11 1.3E-2*	11.3 (1998)	5.4 (2012)	8	6	0.61 −6.4%	
*Athens*	46.7 ± 5.0 1.3E-5***	−1.0 ± 0.15 1.5E-4***	28.1 (1998)	10.7 (2015)	11	7	0.85 0.4%	
*Oslo*	25.1 ± 11.4 0.16	−0.35 ± 0.37 0.44	19.9 (1994)	13.4 (2013)	5	4	0.26 −2.2%	
*Oporto*	−53 ± 207 0.81	+3.53 ± 6.7 0.64	45.6 (2008)	63.2 (2013)	6	4	0.06 −0.6%	
**Czechia**	47.0 ± 23.6 0.12	−1.16 ± 0.87 0.25	25.5 (1998)	8.8 (2012)	7	4	0.23 −11%	
*Prague*	46.8 ± 26.8 0.18	−1.11 ± 1.00 0.35	26.3 (1998)	10.4 (2012)	6	4	0.20 −9.3%	
**Chile**	239 ± 56 5.2E-3**	−5.6 ± 1.49 9.4E-3**	129 (1999)	27.7 (2017)	9	6	0.89 7.4%	
**Singapore**	36.4 ± 37.3 0.36	−0.60 ± 1.14 0.61	21.7 (2004)	14.8 (2015)	10	4	0.04 −1.5%	
**India**	224 ± 90 2.4E-2*	−3.5 ± 2.9 0.23	156 (1999)	92.3 (2017)	19	11	0.09 0.1%	
**Pakistan**	−655 ± 415 0.15	+29 ± 14 7.5E-2.	154 (2007)	337 (2014)	10	4	0.34 0.9%	
*Lahore*	−480 ± 710 0.52	+24 ± 23 0.33	195 (2008)	327 (2014)	9	4	0.11 0.4%	
**Israel**	927 ± 458 0.13	−24.1 ± 12.4 0.15	139 (2012)	22.8 (2017)	6	4	0.40 7.7%	

Additional parameters added to pooled models. Concentrations in μg/m^3^.R^2^_d_ is the pseudo coefficient of determination, along with relative distortion (dist.); FK = Fligner-Killeen non-parametric test on homogeneity of residuals variances. Only results from locations with at least 4 different studies are presented. Data sources, organized geographically and by PM species, in Table S5. Significance codes:

*** <1E-3;

** <1E-2;

* <5E-2; .<1E-1

**Table 9.** Coefficients mean, standard error, and significance for log-normal weighted regression of mean concentrations of indoor PM_10_ (and PM_1–10_) over time: Indoor ~ Intercept + β_1_ * Weight *(Sampling midpoint – 1980).

Location	Intercept	Year since 1980	Earliest estimate	Latest estimate	n of means	n of studies	R^2^_d_ dist.	Pooled models
**USA**	45.7±11.2 1.4E-4***	−1.05±0.43 1.7E-2*	37.4 (1987)	9.7 (2014)	58	31	0.10 −2.5%	
*Baltimore*	−136±49.4 0.2E-2	8.18±2.3 1.7E-1	15.9 (1998)	68.7 (2005)	4	4	0.92 23.7%	
**China**	101±33.4 4.5E-3***	−0.13±1.05 0.90	99.9 (1987)	95.7 (2018)	40	20	0.00 −0.0%	n of means = 64; n of studies = 32 FK p-value =0.1111 R^2^_d_ = 0.71, dist. = −0.4% Intercept: 98.9 ± 31.3; p = 2.6E-3** Year: 0.01 ± 1.0; p = 0.98 No ETS: - 6.3 ± 9.4; p = 0.50 Air cleaners: - 56.5 ± 24.5; p =2.5E-2* Japan: −122 ± 89.5; p = 0.17 Taiwan: 30.3 ± 61.0; p = 0.62 Japan × Year: 3.2 ± 5.1 p = 0.53 Taiwan × Year: −3.1 ± 2.0 p = 0.14
**Japan**	−50.0±67.2 .51	4.46±4.21 .37	16.4 (1994)	104.0 (2014)	6	4	0.26 −7.0%	
**Taiwan**	137±49.9 0.1E-1*	−3.5±1.5 3.7E-2*	91.4 (1993)	13.2 (2015)	18	8	0.41 −1.1%	
**South Korea**	−25.7±40.2 5.3E-1	2.5±1.25 6.9E-2.	34.5 (2004)	63.0 (2015)	15	9	0.20 −1.2%	
*Hong Kong*	86±37 4.4E-2*	−1.7 2.8E-1	57.7 (1996)	21.7 (2018)	12	6	0.06 −0.6%	
*Seoul*	−22±69 7.7E-1	2.4±2.1 0.3	46.5 (2008)	63.0 (2015)	9	6	0.14 −1.3%	
*Taipei*	128±69 1.1E-1	−3.2±2.3 1.9E-1	86.3 (1993)	24.8 (2012)	10	6	0.35 −0.8%	
**Portugal**	−129±102 0.23	5.7±3.3 0.10	34.1 (2008)	66.0 (2014)	17	6	0.15 −0.6%	
**Greece**	56.8±17.6 1.8E-2*	−0.88±0.62 0.21	37.0 (2002)	25.3 (2015)	9	5	0.20 2.6%	Pooled model also includes data from Finland, France, Germany, Netherlands, Norway, and Sweden n of means = 56; n of studies = 31 FK p-value = 0.05062 R^2^_d_ = 0.81, dist. = −0.1% Intercept: −11.7 ± 45.7; p = 0.80 Year: 0.88 ± 1.48; p = 0.55 No ETS: −3.3 ± 11.2; p = 0.77 France: 153 ± 50; p = 3.7E-3** Sweden: −859 ± 269; p = 2.9E-3** France × Year: −4.4 ± 1.6; p = 9.5E-3** Sweden × Year: 29.5 ± 9.1; p = 2.6E-3** All other country and interaction terms not significant at 0.05 level.
**Italy**	95.6 ± 23.7 5.6E-2 ·	−2.1 ± 0.6 8.5E-2 ·	38.9 (2007)	18.3 (2017)	5	4	0.85 0.3%	
**United Kingdom**	42.2 ± 10.3 7.9E-4***	−0.50 ± 0.51 0.34	34.5 (1995)	29.1 (2006)	20	6	0.05 −0.2%	
*Athens*	56.8 ± 17.6 1.8E-2*	−0.9 ± 0.6 0.21	37.0 (2002)	25.3 (2015)	9	5	0.20 2.6%	
**India**	389 ± 141 1.9E-2	−6.3 ± 4.9 0.23	267 (1999)	154 (2017)	14	10	0.13 0.5%	
*Delhi*	552 ± 1018 0.68	−14.8 ± 49.3 0.81	267 (1999)	186 (2004)	4	4	0.03 −1.7%	
**Thailand**	126 ± 85 0.24	−2.8 ± 2.3 0.31	80.6 (1996)	19.9 (2017)	6	4	0.54 6.2%	

Additional parameters added to pooled models. Concentrations in μg/m^3^. R^2^_d_ is the pseudo coefficient of determination, along with relative distortion (dist.); FK = Fligner-Killeen non-parametric test on homogeneity of residuals variances. Only results from locations with at least 4 different studies are presented. Data sources, organized geographically and by PM species, in Table S5. Significance codes:

*** <1E-3;

** <1E-2;

* <5E-2; .<1E-1

**Table 10.** Coefficients mean, standard error, and significance for log-normal weighted regression of mean concentrations of indoor PM_1_ (and PM_0.1–1_) over time: Indoor ~ Intercept + β_1_ * Weight *(Sampling midpoint – 1980).

Location	Intercept	Year since 1980	Earliest estimate	Latest estimate	n of means	n of studies	R^2^_d_ dist.
**Western Europe**	24.4 ± 12.6 6.5E-2 .	−0.63 ± 0.38 0.11	12.7 (1998)	1.6 (2015)	26	15	0.11 0.1%
**East Asia**	13.5 ± 21.0 0.53	0.68 ± 0.66 0.32	27.1 (1999)	38.0 (2015)	16	9	0.06 0.3%
*China*	213 ± 327 0.54	−4.9 ± 9.2 0.61	55.6 (2011)	36.2 (2015)	10	5	0.05 −1.6%
*Taiwan*	8.5 ± 16.9 0.65	0.88 ± 0.69 0.29	25.9 (1999)	36.6 (2012)	6	4	0.30 0.1%
**North America**	−150 ± 105 0.22	5.4 ±. 3.5 0.20	2.0 (2008)	46.1 (2016)	7	5	0.09 −13%
**South Asia**	302 ± 48 2.7E-5***	−6.9 ± 1.6 7.1E-4***	144 (2003)	42.9 (2017)	16	6	0.41 0.1%

Concentrations in μg/m^3^. R^2^_d_ is the pseudo coefficient of determination, along with relative distortion (dist.). Only results from locations with at least 4 different studies are presented. Data sources, organized geographically and by PM species, in Table S5. Significance codes:

*** <1E-3

** <1E-2

* <5E-2 .<1E-1

**Table 11.** Coefficients mean, standard error, and significance for log-normal weighted regression of mean concentrations of indoor UFP (and PM_0.25_ and PM_0.1_) over time: Indoor ~ Intercept + β_1_ * Weight *(Sampling midpoint – 1980).

Location	Intercept	Year since 1980	Earliest estimate	Latest estimate	n of means	n of studies	R^2^_d_ dist.
**Western Europe**	−19 123 ± 19 053 0.33	1 098 ± 612 8.6E-2 .	5 539 (2002)	19 814 (2015)	25	12	0.09 3.2%
**North America**	−3 728 ± 5 967 0.54	474 ± 202 3E-2*	8 400 (2005)	13 556 (2016)	18	10	0.26 −1.2%
*USA*	−1 403 ± 69 635 0.99	411 ± 2 614 0.88	9 286 (2006)	13 031 (2015)	7	4	0.01 −0.2%
*Canada*	−7 906 ± 5 163 0.16	595 ± 166 7.2E-3**	7307 (2005)	13774 (2016)	11	6	0.55 −2.9%
**East Asia**	70 696 ± 41 947 0.19	−1 023 ± 1 158 0.44	58 126 (1992)	32 520 (2017)	6	4	0.24 2.1%

Concentrations in cm^−3^. R^2^_d_ is the pseudo coefficient of determination, along with relative distortion (dist.); s = standard deviation of model. Only results from locations with at least 4 different studies are presented. Data sources, organized geographically and by PM species, in Table S5..Significance codes:

*** <1E-3;

** <1E-2;

* <5E-2; .<1E-1

**Table 12.** Coefficients mean, standard error, and significance for log-normal weighted regression of mean concentrations of indoor PM_2.5_ (and PM_0.25–2.5_) with respect to concentrations measured outdoor (on site): Indoor ~ Intercept + β_1_ * Weight *Outdoor + β_2_ * ETS excluded (True or False).

Location	Intercept	Outdoor (β_1_)	ETS excluded (β_2_)	R^2^_d_ dist.	n of means pairs	n of studies
Global model	4.1 ± 0.6 2.3E-10***	0.86 ± 0.05 <2.0E-16***	−1.6 ± 0.5 1.9E-3**	0.72 8.2%	389	149
Global model, only outdoor concentrations ≤ 50	3.2 ± 0.8 8.4E-5***	0.95 ± 0.06 <2.0E-16***	−1.7 ± 0.6 4.1E-3**	0.51 7.8%	290	97
Global model, only outdoor concentrations ≤ 25	2.0 ± 0.9 3.2E-2*	1.07 ± 0.08 <2.0E-16***	−1.5 ± 0.6 1.9E-3**	0.47 6.6%	219	81
Global model, only pairs excluding ETS	3.8 ± 1.1 4.2E-4***	0.75 ± 0.08 2.4E-16***		0.55 2.4%	150	59
North America	2.6 ± 1.0 1.4E-2*	1.04 ± 0.09 <2.0E-16***	−2.7 ± 0.6 4.5E-3**	0.43 5.1%	149	48
*Canada*	1.8 ± 1.4 0.19	0.87 ± 0.16 2.0E-5***	−3.2 ± 1.1 7.7E-3**	0.55 −2.9%	27	9
*USA*	7.0 ± 1.6 3.2E-5***	0.79 ± 0.13 1.1E-8***	−2.8 ± 0.9 2.7E-3**	0.30 2.5%	122	39
East Asia	14.3 ± 2.8 2.2E-6***	0.48 ± 0.04 <2.0E-16***	−8.0 ± 2.5 2.0E-3**	0.86 9.1%	86	31
*China*	19.8 ± 3.8 2.1E-6***	0.37 ± 0.06 6.5E-9***	6.9 ± 6.2 0.26	0.58 2.7%	68	23
*Taiwan*	26.2 ± 3.8 3.E-5***	0.31 ± 0.03 6.5E-5***	3.9 ± 2.2 0.14	0.96 0.1%	9	4
Western Europe	5.8 ± 1.2 1.9E-5***	0.64 ± 0.08 8.1E-12***	−2.9 ± 0.8 1.2E-3**	0.70 1.1%	60	26
*Finland*	9.9 ± 5.3 0.20	0.07 ± 0.45 0.89	−2.3 ± 2.4 0.45	0.25 0.2%	6	4
*Greece*	9.4 ± 6.0 0.18	0.52 ± 0.23 0.07 .	−4.6 ± 3.8 0.27	0.51 −0.4%	9	5
*Italy*	5.4 ± 6.9 0.46	0.67 ± 0.39 0.13	−0.3 ± 10.6 0.98	0.29 −2.6%	12	5
*UK*	11.2 ± 4.2 3.1E-2*	0.39 ± 0.23 0.13	−1.3 ± 4.6 0.79	0.32 −1.8%	11	5
Eastern Europe	0.4 ± 2.3 0.87	0.58 ± 0.14 4.0E-4***	17.7 ± 6.0 8.3E-3**	0.61 −1.0%	24	7
West Asia & North Africa	2.3 ± 3.6 0.53	1.09 ± 0.14 5.9E-7***	−16.8 ± 2.3 9.8E-7***	0.98 5.1%	22	7
South America	6.1 ± 5.0 0.25	0.93 ± 0.13 5.3E-6***	4.4 ± 3.9 0.27	0.90 0.3%	18	6
*Chile*	48.8 ± 17.0 2.1E-2*	0.27 ± 0.28 0.35	−6.4 ± 8.5 0.46	0.14 0.5%	12	4
Central America & Caribbean	27.1 ± 5.4 4.9E-4***	0.50 ± 0.11 1.4E-3**	−12.1 ± 3.6 6.8E-3**	0.80 0.5%	14	2

Concentrations in μg/m^3^. Weight is total sampling time. Only results from locations with at least 4 different studies are presented. Significance codes:

*** <1E-3;

** <1E-2;

* <5E-2; .<1E-1

**Table 13.** Coefficients mean, standard error, and significance for log-normal weighted regression of mean concentrations of indoor PM_2.5_ (and PM_0.25–2.5_) with respect to concentrations measured at nearest ambient air monitor (not on site of indoor measurements): Indoor ~ Intercept + β_1_ * Weight *Ambient + β_2_ * ETS excluded (True or False).

Location	Intercept	Ambient (not on site) (β_1_)	ETS excluded (β_2_)	R^2^_d_ dist.	n of means pairs	n of studies
Global model	6.1 ± 1.3 2.1E-5***	0.49 ± 0.07 3.0E-9***	−0.01 ± 1.3 0.995	0.56 7.3%	78	36
Global model, only pairs excluding ETS	7.7 ± 1.5 2.0E-5***	0.41 ± 0.11 5.6E-4***		0.37 12.0%	46	22
North America	0.3 ± 3.1 0.92	1.13 ± 0.31 1.2E-3**	0.2 ± 1.7 0.93	0.46 1.9%	30	16
*USA*	0.6 ± 3.3 0.84	1.09 ± 0.33 3.5E-3**	0.6 ± 1.8 0.75	0.44 2.3%	26	15
East Asia	24.5 ± 8.1 8.5E-3**	0.16 ± 0.14 0.28	−17.8 ± 5.1 3.3E-3**	0.45 −4.5%	19	11
*China*	7.7 ± 9.2 0.42	0.51 ± 0.19 1.9E-2*	−28.7 ± 5.3 1.1E-4***	0.46 −5.2%	17	10
Western Europe	0.6 ± 1.6 0.71	0.92 ± 0.15 3.1E-6***	1.7 ± 1.8 0.35	0.61 −6.7%	26	6

Concentrations in μg/m^3^. Weight is total sampling time. Only results from locations with at least 4 different studies are presented. Significance codes:

*** <1E-3;

** <1E-2;

* <5E-2; .<1E-1

**Table 14.** Coefficients mean, standard error, and significance for log-normal weighted regression of mean concentrations of indoor PM_10_ (and PM_1–10_) with respect to concentrations measured outdoor (on site) or, when specified, at an ambient monitor (not on site): Indoor ~ Intercept + β_1_ * Weight *Outdoor + β_2_ * ETS.excluded (True or False).

Location	Intercept	Outdoor (β_1_)	ETS excluded (β_2_)	R^2^_d_ dist.	n of means pairs	n of studies
Global model	11.6 ± 1.4 3.6E-15***	0.63 ± 0.04 <2.0E-16***	Regression with β_2_ not converging	0.69 1.3%	206	76
Global model, only pairs excluding ETS	12.0 ± 2.6 2.4E-5***	0.69 ± 0.09 2.1E-10***		0.67 1.7%	74	34
Global model, ambient monitor (not on site)	13.2 ± 6.2 4.4E-2*	0.64 ± 0.23 1.0E-3**	−6.4 ± 5.4 0.25	0.41 1.4%	28	12
East Asia	19.7 ± 4.5 4.5E-5***	0.62 ± 0.04 <2.0E-16***	−12.7 ± 3.5 5.7E-4***	0.89 5.9%	66	20
East Asia, only pairs excluding ETS	3.7 ± 4.9 0.46	0.68 ± 0.08 1.3E-6***		0.90 1.9%	17	9
North America	0.3 ± 3.7 0.94	1.2 ± 0.17 3.0E-8***	−3.6 ± 2.5 0.16	0.67 3.9%	43	20
North America, only pairs excluding ETS	2.0 ± 3.3 0.54	0.93 ± 0.15 1.5E-5***		0.81 7.8%	19	12
Western Europe	7.9 ± 2.9 1.0E-2**	0.62 ± 0.10 4.2E-7***	−4.0 ± 2.2 7.5E-2 .	0.67 2.7%	37	16
Western Europe, only pairs excluding ETS	2.6 ± 1.6 0.17	0.70 ± 0.09 1.4E-3**		0.96 0.6%	7	4
West Asia & North Africa	7.2 ± 13.2 0.59	0.58 ± 0.16 1.9E-3**	1.8 ± 8.0 0.82	0.57 −15%	21	6
Eastern Europe	8.9 ± 4.6 7.6E-2 .	0.38 ± 0.13 1.1E-2*	25.9 ± 7.5 4.2E-3**	0.69 2.9%	17	4

Concentrations in μg/m^3^. Weight is total sampling time. Only results from locations with at least 4 different studies are presented. Significance codes:

*** <1E-3

** <1E-2

* <5E-2 .<1E-1

**Table 15.** Coefficients mean, standard error, and significance for log-normal weighted regression of mean concentrations of indoor PM_2.5–10_ (and PM_2–10_) with respect to concentrations measured outdoor (on site): Indoor ~ Intercept + β_1_ * Weight *Outdoor + β_2_ * ETS excluded (True or False).

Location	Intercept	Outdoor (β_1_)	ETS excluded (β_2_)	R^2^_d_ dist.	n of means pairs	n of studies
Global model	2.0 ± 1.2 9.6E-1 .	0.74 ± 0.15 1.2E-5***	1.8 ± 1.2 3.8E-3**	0.47 1.8%	51	20
Global model, only pairs excluding ETS	3.0 ± 1.8 0.10	0.83± 0.21 4.2E-4***		0.40 2.4%	31	14
North America	4.5 ± 1.8 2.3E-2*	0.33 ± 0.22 0.17	0.2 ± 1.5 0.87	0.09 1.1%	23	10
East Asia	2.1 ± 1.7 0.26	0.74 ± 0.22 1.3E-6***	−1.0 ± 1.5 0.53	1.02 3.4%	12	4

Concentrations in μg/m^3^. Weight is total sampling time. Only results from locations with at least 4 different studies are presented. Significance codes:

*** <1E-3;

** <1E-2;

* <5E-2; .<1E-1

**Table 16.** Coefficients mean, standard error, and significance for log-normal weighted regression of mean concentrations of indoor PM_1_ (and PM_0.1–1_) with respect to concentrations measured outdoor (on site): Indoor ~ Intercept + β_1_ * Weight *Outdoor + β_2_ * ETS excluded (True or False).

Location	Intercept	Outdoor (β_1_)	ETS excluded (β_2_)	R^2^_d_ dist.	n of means pairs	n of studies
Global model	2.3 ± 0.5 1.1E-5***	0.55 ± 0.04 <2.0E-16***	−2.3 ± 0.8 3.8E-3**	0.76 −13%	52	16
Global model, only pairs excluding ETS	2.8 ± 1.6 0.11	0.38 ± 0.08 5.5E-4***		0.53 −22%	13	4

Concentrations in μg/m^3^. Weight is total sampling time. Only results from locations with at least 4 different studies are presented. Significance codes:

*** <1E-3;

** <1E-2;

* <5E-2; .<1E-1

**Table 17.** Coefficients mean, standard error, and significance for log-normal weighted regression of mean concentrations of indoor UFP (and PM_0.1_) with respect to concentrations measured outdoor (on site): Indoor ~ Intercept + β_1_ * Weight *Outdoor + β_2_ * ETS excluded (True or False).

Location	Intercept	Outdoor (β_1_)	ETS excluded (β_2_)	R^2^_d_ dist.	n of means pairs	n of studies
Global model	1 654 ± 868 7.5E-2 .	0.79 ± 0.14 4.8E-5***	−2 207 ± 1 479 0.16	0.66 −5.7%	20	9
Global model, only pairs excluding ETS	−1 366 ± 1 973 0.50	0.86 ± 0.18 6.8E-4***		0.64 −3.3%	13	5

Concentrations in cm^−3^. Weight is total sampling time. Only results from locations with at least 4 different studies are presented. Significance codes:

*** <1E-3;

** <1E-2;

* <5E-2; .<1E-1

## Data Availability

The data that support the findings of this study are available in peer-reviewed publications. Data sources, by country or territory, that were used to compile the data are listed in the Supplementary Information.

## Practical implications

- Residential indoor PM concentration ranges for several size fractions measured in different worldwide regions are provided and may inform future public health research and practice, including PM exposure and risk assessment, and evaluation of IAQ-related interventions and consumer products, such as portable air cleaners.
- This long-term indoor PM concentration record provides insights regarding the degree of change in observed indoor PM concentrations by world region and some of the factors contributing to increasing or decreasing temporal trends.
- Outdoor air pollution remains a major influence on indoor concentrations of PM of all sizes.
- Greater variability of indoor concentrations of PM relative to outdoor concentrations demonstrates the potential for exposure misclassification when using outdoor concentrations to estimate indoor exposures and risk.
- IAQ interventions, including removing environmental tobacco smoke or using filtration-based portable air cleaners, can produce major improvements in IAQ through reduced indoor particle concentrations.
