# Supplementary material for: Survey of residential indoor particulate matter measurements 1990–2019
Source: Indoor Air. Author manuscript; Available in PMC 2023 Sep 13. (PMC10499005; doi:10.1111/ina.13057)
Supplement: Author manuscript Supplementary info [file NIHMS1930196-supplement-Author_manuscript_Supplementary_info.pdf]

Supplemental Material for

Survey of Residential Indoor

Particulate Matter measurements 1990-2019

Vito Ilacqua[1*], Nicole Scharko[2], Jordan Zambrana[1], Daniel Malashock[1]

1. United States Environmental Protection Agency, Office of Radiation and Indoor Air, Indoor Environments Division
2. American Association for the Advancement of Science (AAAS) - Science, Technology, and Policy Fellow

* Corresponding author

Contents

Table S1. Summary of existing literature reviews of indoor PM. 2

Table S2. Performance of calculated mean .... **Error! Bookmark not defined.**

Figure S1. Distribution of total sampling times (hr) used as weighting factor for the analyses 4

Figure S2. Violin plots of PM_{10}, $PM_{2.5\text{-}10}$ and $PM_{2.5}$ for Central America and Caribbean, Eastern Europe, South Asia and West Asia & North Africa.. 5

Figure S3. Violin plots of PM_1 and UFP (in µg/m^3 and cm^{-3}) for Eastern Europe, South Asia and West Asia & North Africa. 6

Figure S4. Violin plots of PM_{10}, $PM_{2.5\text{-}10}$ and $PM_{2.5}$ for Africa (except North), Oceania, South America and South East Asia. 7

Figure S5. Violin plots of PM_1 and UFP (in µg/m^3 and cm^{-3}) for Africa (except North), Oceania, South America and South East Asia.. 8

Table S3. Statistics for indoor means from studies that both included and excluded ETS. 9

Table S4. Statistics for indoor means from studies with and without air cleaners. 9

Table S5. List of publications with data used in the survey, by country. 10

References 136

Table S1. Summary of existing literature reviews of indoor PM.

Table S1a. Studies that reviewed residential environments and do not provide pooled statistics.							
**Study**	**Timeframe***	**Geography**	**Indoor micro-environment**	**Particle metrics of interest**	**Individual study statistics provided**	**# of Studies Reviewed**	**Additional notes**
Vardoulakis et al., 2020	2000-2017	No limitations	Domestic indoor environments	$PM_{2.5}$ and PM_{10} (also include studies with $PM_{2.5-10}$, PNC, UFP etc.)	In the main manuscript they do provide the range for mean concentrations for $PM_{2.5}$ and PM_{10}.	73 studies for $PM_{2.5}$ and 37 studies for PM_{10}.	Excluded studies published in other languages and biomass burning homes.
Ye et al., 2017	last 10 years (assuming 2006-2016)	China (separated by rural and urban)	Chinese residential buildings	PM_1, $PM_{2.5}$ and PM_{10}	Min, max and mean.	11 studies for urban and 7 studies for rural (for all PM species)	Only English and Chinese literature reviewed. Studies needed normal ventilation conditions.
Li et al., 2017	2000-2016	No limitations	All indoor environments (restaurant, residence, school etc.)	$PM_{2.5}$	Statistics provided in their review table varied depending on the study results.	11 studies with residential homes as the micro-environment	Only English and Chinese literature reviewed.

Table S1b. Studies that reviewed residential environments and report pooled statistics.							
**Study**	**Timeframe***	**Geography**	**Indoor micro-environment**	**Particle metrics of interest**	**Summary statistics provided and quantitative approach**	**# of Studies used in analysis**	**Additional notes**
Logue et al., 2011	1995–2010	US and other indus-trialized nations	residences	$PM_{2.5}$	Mean, 25th, 50th, 75, and 95 percentiles. Studies were weighted by the number of measurements within each study	13 studies for PM2.5 used to determine weighted mean	One of their study reviews was a new home, and the authors do provide statistics for that home for comparison purpose.
Morawska et al., 2013	January 1989 and October 2012	No limitation	Residences, personal and schools	PM_{10}, $PM_{2.5}$ and PN	Min, 1st quartile, median, 3rd quartile and max. Summary statistics were obtained from average values reported in the studies.	44 studies for all PM species: PM_{10}, $PM_{2.5}$ and PN. (residential environments)	Smoking excluded, biomass burning excluded.
Morawska et al., 2017	1990–2017	No limitation	Homes, schools and day cares, offices, and aged care facilities	PM_{10}, $PM_{2.5}$ and PN	Mean and standard errors (SE), averaging time 24 h. Summary statistics were obtained from weighing the number of individual locations in each study; assumed to be 1 if not reported	5 studies for PM_{10}, 7 studies for $PM_{2.5}$ and 5 studies for PN. (with residential homes as the microenvironment)	Smoking and unoccupied homes, biomass burning homes or homes where normal activity was not allowed were excluded. studies needed a mean, standard deviation, and averaging period of 24 h or multiple of 24.

***Timeframe or Paper range used in review for homes**

Table S2. Performance of calculated means and standard deviations where both actual and calculated value exist.

**All values (mean = 385 pairs and SD =283 pairs)**				
Statistical measure	RD_{Mean} = (Act Mean- Calc Mean)/Act Mean	AD_{Mean} = abs(Act Mean- Calc Mean)/Act Mean	RD_{SD}= (Act SD- Calc SD)/Act SD	AD_{SD}= abs(Act SD- Calc SD)/Act SD
Min	-3.76	0.0004	-12.3	0.0001
1st Qu.	-0.044	0.029	-0.117	0.092
Median	0.020	0.064	0.074	0.189
Mean	-0.001	0.126	-0.077	0.370
3rd Qu.	0.084	0.133	0.230	0.355
Max	0.513	3.76	0.751	12.3
**Indoor values only (mean = 249 pairs and SD = 174 pairs)**				
Statistical measure	RD_{Mean} = (Act Mean- Calc Mean)/Act Mean	AD_{Mean} = abs(Act Mean- Calc Mean)/Act Mean	RD_{SD}= (Act SD- Calc SD)/Act SD	AD_{SD}= abs(Act SD- Calc SD)/Act SD
Min	-3.76	0.001	-12.3	0.0001
1st Qu.	-0.051	0.033	-0.114	0.092
Median	0.018	0.069	0.079	0.185
Mean	-0.010	0.145	-0.111	0.415
3rd Qu.	0.098	0.151	0.227	0.415
Max	0.513	3.76	0.741	12.3
**Outdoor values only (mean = 110 pairs and SD = 84 pairs)**				
Statistical measure	RD_{Mean} = (Act Mean- Calc Mean)/Act Mean	AD_{Mean} = abs(Act Mean- Calc Mean)/Act Mean	RD_{SD}= (Act SD- Calc SD)/Act SD	AD_{SD}= abs(Act SD- Calc SD)/Act SD
Min	-1.84	0.001	-7.13	0.001
1st Qu.	-0.035	0.026	-0.164	0.112
Median	0.023	0.055	0.025	0.219
Mean	0.014	0.102	-0.070	0.340
3rd Qu.	0.076	0.100	0.243	0.342
Max	0.419	1.84	0.751	7.13
**Ambient values only (mean = 26 pairs and SD = 25 pairs)**				
Statistical measure	RD_{Mean} = (Act Mean- Calc Mean)/Act Mean	AD_{Mean} = abs(Act Mean- Calc Mean)/Act Mean	RD_{SD}= (Act SD- Calc SD)/Act SD	AD_{SD}= abs(Act SD- Calc SD)/Act SD
Min	-0.073	0.000	-0.218	0.026
1st Qu.	-0.007	0.019	0.074	0.082
Median	0.024	0.041	0.110	0.173
Mean	0.025	0.047	0.135	0.161
3rd Qu.	0.064	0.072	0.206	0.218
Max	0.156	0.156	0.447	0.447

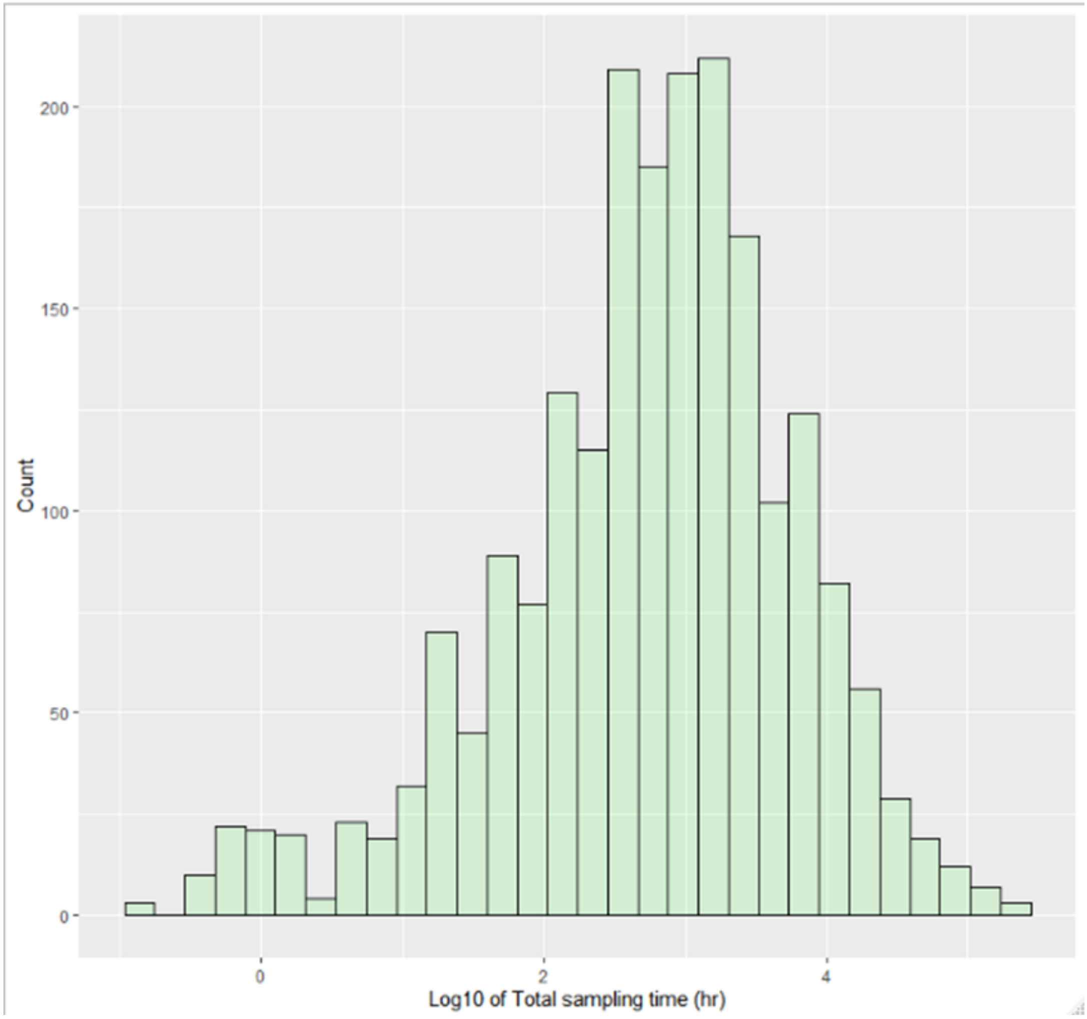

Figure S1. Distribution of total sampling times (hr) used as weighting factor for the analyses. Values on a log scale.

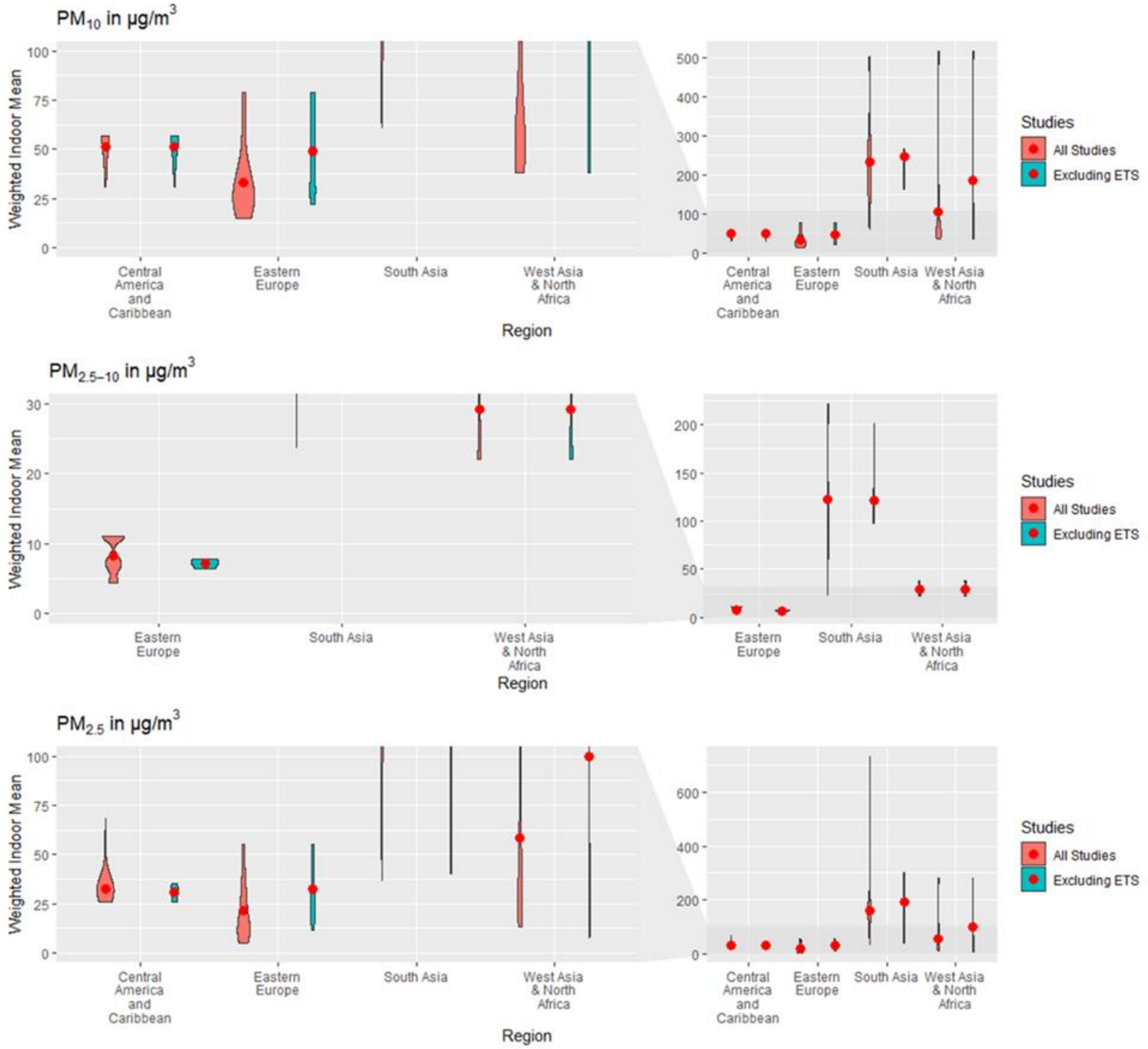

Figure S2. Violin plots of PM_{10}, $PM_{2.5\text{-}10}$ and $PM_{2.5}$ for Central America and Caribbean, Eastern Europe, South Asia and West Asia & North Africa. The red points are displaying the weighted indoor mean, and the density profiles are displaying the weighted distribution of the mean. Studies labeled 'Excluded ETS' are restricted to mean measurements in homes without environmental tobacco smoke.

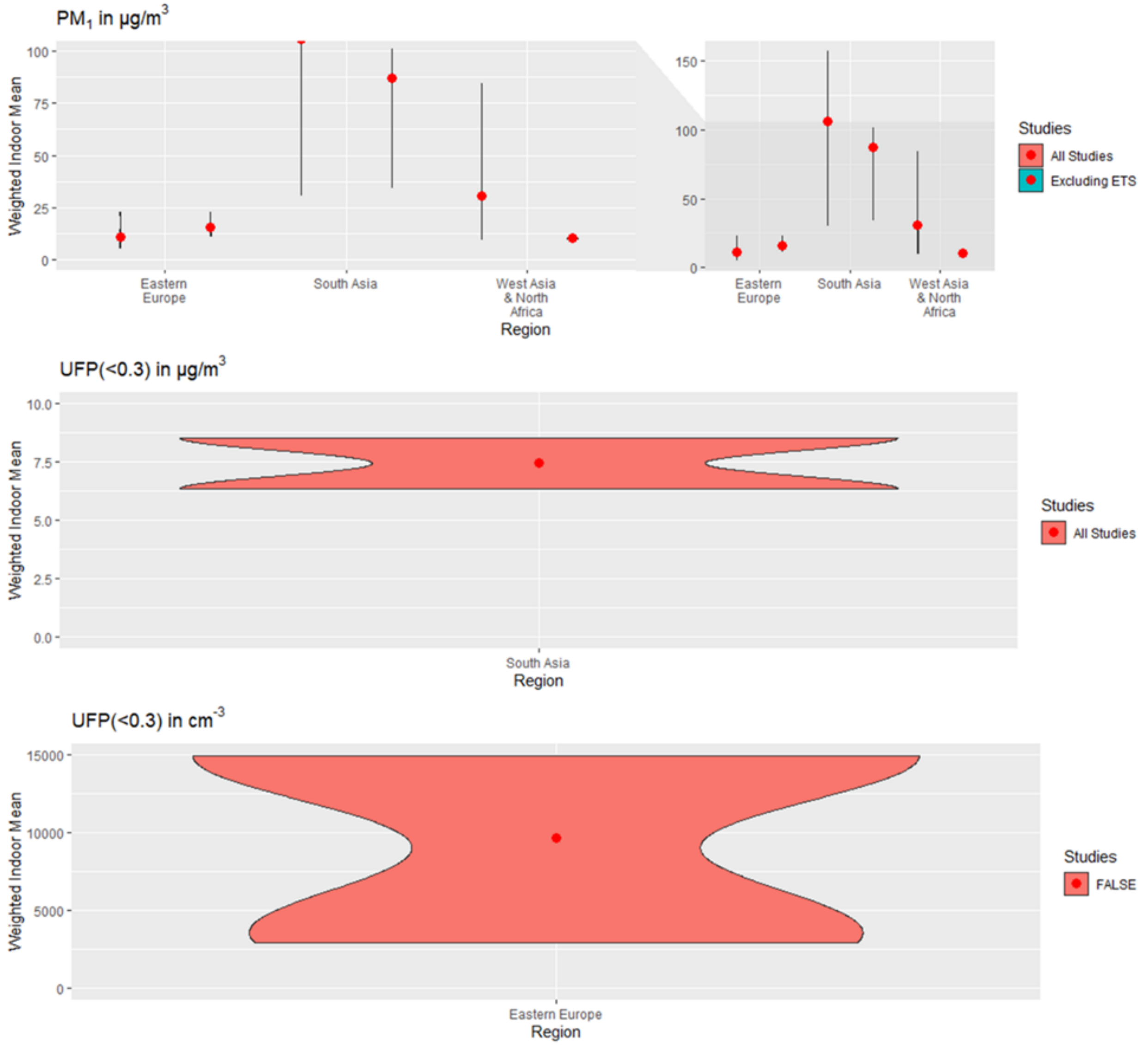

Figure S3. Violin plots of PM_1 and UFP (in µg/m^3 and cm^{-3}) for Eastern Europe, South Asia and West Asia & North Africa. The red points are displaying the weighted indoor mean, and the density profiles are displaying the weighted distribution of the mean. Studies labeled 'Excluded ETS' are restricted to mean measurements in homes without environmental tobacco smoke.

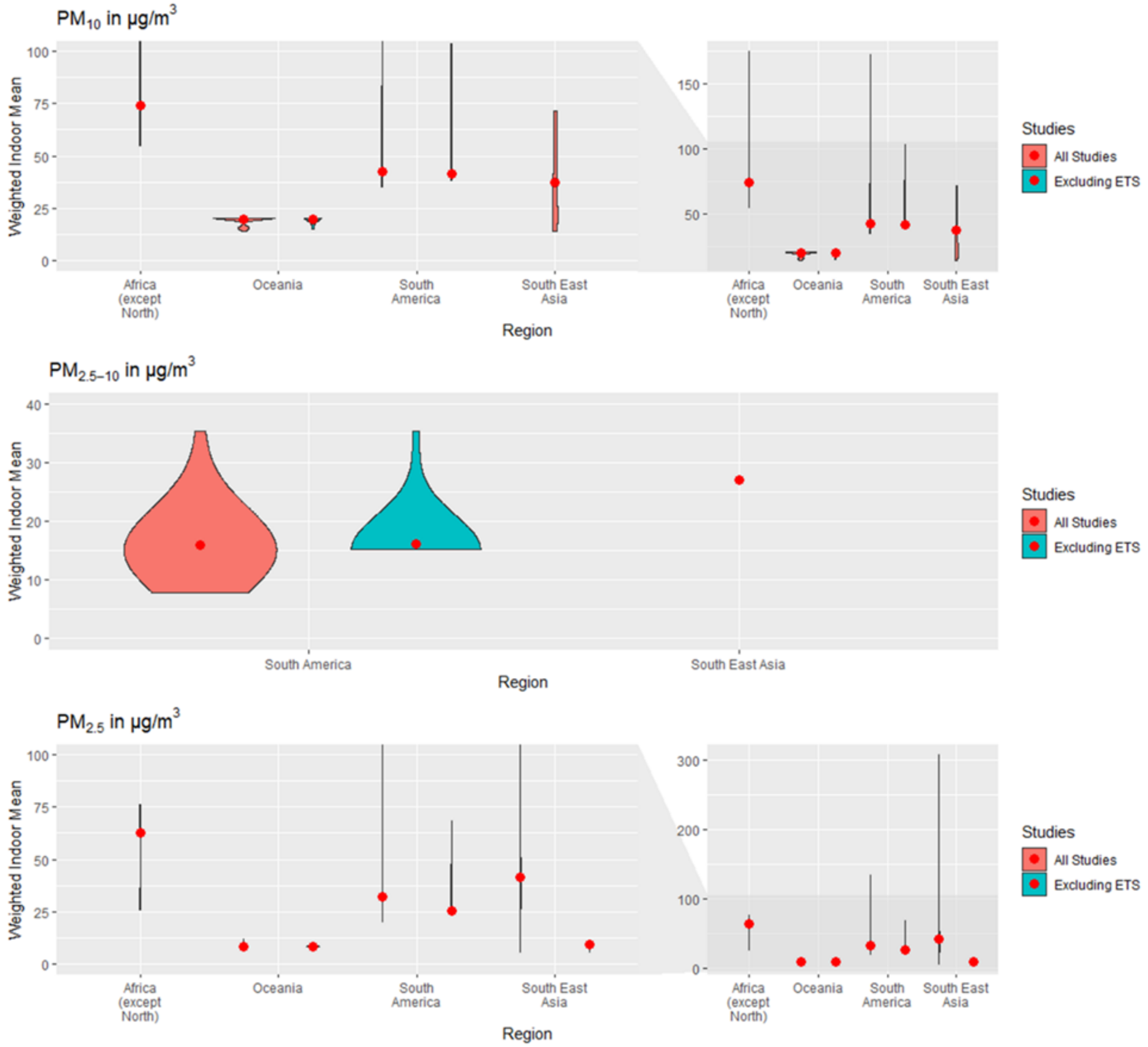

Figure S4. Violin plots of PM_{10}, $PM_{2.5\text{-}10}$ and $PM_{2.5}$ for Africa (except North), Oceania, South America and South East Asia. The red points are displaying the weighted indoor mean and the density profiles, are displaying the weighted distribution of the mean. Studies labeled 'Excluded ETS' are restricted to mean measurements in homes without environmental tobacco smoke.

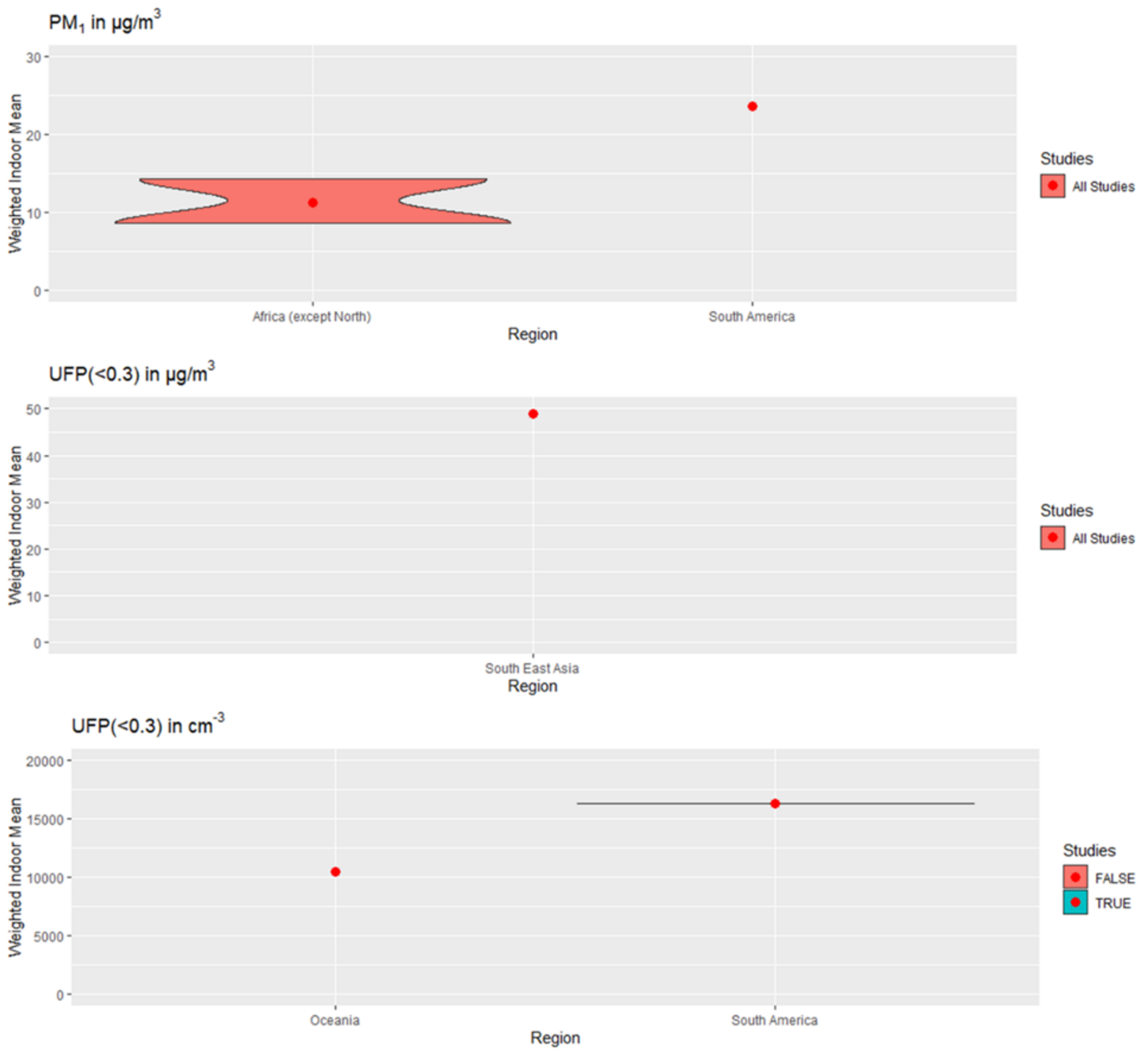

Figure S5. Violin plots of PM_1 and UFP (in µg/m^3 and cm^{-3}) for Africa (except North), Oceania, South America and South East Asia. The red points are displaying the weighted indoor mean and the density profiles are displaying the weighted distribution of the mean. Studies labeled 'Excluded ETS' are restricted to mean measurements in homes without environmental tobacco smoke.

Table S3. Statistics for indoor means from studies that both included and excluded ETS. Statistics labeled 'Without ETS' are restricted to measurements in homes without environmental tobacco smoke, while 'With ETS' contains measurements in homes with and without environmental tobacco smoke. Wilcoxon–Mann–Whitney test was carried out on the original means and the W (statistic value) and p-values (significance) are provided in the table. †The exact p-value was not computed; a normal approximation was used.

PM species, unit and with or without ETS		Studies	Homes	Statistics for Mean Measurements						Wilcoxon–Mann–Whitney statistics	
				n	Mean	SD	Wt. Mean	Min	Max	W	p-value
$PM_{2.5}$ ($\mu g/m^3$)	With ETS	46	3302	87	46.1	57.9	25.9	2.7	344	4226.5	0.0065
	Without ETS	46	1954	78	36.7	52.0	21.9	2.8	302		
$PM_{2.5-10}$ ($\mu g/m^3$)	With ETS	5	160	20	17.3	24.8	12.3	3.4	119.2	84	0.84†
	Without ETS	5	53	8	23.0	39.7	20.0	5.5	120.3		
PM_{10} ($\mu g/m^3$)	With ETS	28	1291	49	68.3	77.8	45.1	5.5	425	1158	0.14†
	Without ETS	28	746	40	66.3	92.6	69.9	5.6	517.74		
PM_1 ($\mu g/m^3$)	With ETS	6	184	12	38.3	42.1	27.5	5	156.3	57	0.23
	Without ETS	6	142	7	25.7	35.8	21.9	3	101.1		
Number (cm^{-3})	With ETS	3	203	8	14223	12098	10296	36.8	31816	25	0.15
	Without ETS	3	84	4	4964	5894	1608	29.1	11815		

Table S4. Statistics for indoor means from studies with and without air cleaners. Statistics labeled 'No air cleaner' are restricted to measurements in homes without air cleaners, yet the corresponding study contains measurements with air cleaners. Statistics labeled 'Air cleaner' contains measurements in homes with air cleaners. Wilcoxon–Mann–Whitney test was carried out on the original means and the W (statistic value) and p-values (significance) are provided in the table. †The exact p-value was not computed; a normal approximation was used.

PM species, unit and with or without air cleaner		Studies	Homes	Statistics for Mean Measurements						Wilcoxon–Mann–Whitney statistics	
				n	Mean	SD	Wt. Mean	Min	Max	W	p-value
$PM_{2.5}$ ($\mu g/m^3$)	No air cleaner	21	591	30	37.0	33.9	22.2	4.61	138.3	624	0.0042†
	Air cleaner	21	520	29	18.3	18.4	10.6	2.22	75.83		
$PM_{2.5-10}$ ($\mu g/m^3$)	No air cleaner	3	82	4	21.2	20.6	15.9	5.7	51.2	10	0.69
	Air cleaner	3	78	4	14.9	13.4	12.8	3.8	34.1		
PM_{10} ($\mu g/m^3$)	No air cleaner	3	70	3	45.7	24.2	27.1	18.1	63	7	0.40
	Air cleaner	3	70	3	26.5	18.6	7.2	5	38		
PM_1 ($\mu g/m^3$)	No air cleaner	2	40	2	47.6	0.8	47.2	47	48.2	4	0.33
	Air cleaner	2	40	2	28.6	3.7	27.1	25.9	31.2		
Number (cm^{-3})	No air cleaner	3	58	3	6903	11648	17787	63.4	20352	6	0.70
	Air cleaner	3	71	3	4090	6922	10086	29.1	12083		

Table S5. List of publications with data used in the survey, by PM species, world region, and country (or territory).

Light Scattering

North America — Light Scattering

USA — Light Scattering

Anuszewski J, TV Larson, JQ Koenig	1998	Simultaneous indoor and outdoor particle light-scattering measurements at nine homes using a portable nephelometer	Journal of Exposure Analysis and Environmental Epidemiology	8	4	483-493

Western Europe — Light Scattering

Belgium — Light Scattering

Dons E, LI Panis, M Van Poppel, J Theunis, H Willems, R Torfs, G Wets	2011	Impact of time-activity patterns on personal exposure to black carbon	Atmospheric Environment	45	21	3594-3602

Number (size cut-offs unspecified)

East Asia — Number

China — Number

Mullen NA, C Liu, YP Zhang, SX Wang, WW Nazaroff	2011	Ultrafine particle concentrations and exposures in four high-rise Beijing apartments	Atmospheric Environment	45	40	7574-7582

Japan — Number

Kanatani K, M Okumura, S Tohno, Y Adachi, K Sato, T Nakayama	2014	Indoor particle counts during Asian dust events under everyday conditions at an apartment in Japan	Environmental Health and Preventive Medicine	19	1	81-88

Ma CJ, GU Kang, CH Kang	2015	Properties of Indoor Particles Collected in Japanese Homes	Asian Journal of Atmospheric Environment	9	1	31-38

North America Number

Canada Number

Kovesi T, D Creery, NL Gilbert, R Dales, D Fugler, B Thompson, N Randhawa,	2006	Indoor air quality risk factors for severe lower respiratory tract infections in Inuit infants in Baffin Region, Nunavut: a pilot study	Indoor Air	16	4	266-275

USA Number

Batterman S, C Godwin, CR Jia	2005	Long duration tests of room air filters in cigarette smokers' homes	Environmental Science & Technology	39	18	7260-7268
Batterman S, L Du, G Mentz, B Mukherjee, E Parker, C Godwin, JY Chin,	2012	Particulate matter concentrations in residences: an intervention study evaluating stand-alone filters and air conditioners	Indoor Air	22	3	235-252
Brugge D, MC Simon, N Hudda, M Zellmer, L Corlin, S Cleland, EY Lu, S Rivera,	2017	Lessons from in-home air filtration intervention trials to reduce urban ultrafine particle number concentrations	Building and Environment	126		266-275
Hudda N, MC Simon, W Zamore, JL Durant	2018	Aviation-Related Impacts on Ultrafine Particle Number Concentrations Outside and Inside Residences near an Airport	Environmental Science & Technology	52	4	1765-1772
Less, B., Mullen, N., Singer, B., & Walker, I	2015	Indoor air quality in 24 California residences designed as high-performance homes	Science and Technology for the Built Environment	21	1	14-24
Singer BC, RZ Pass, WW Delp, DM Lorenzetti, RL Maddalena	2017	Pollutant concentrations and emission rates from natural gas cooking burners without and with range hood exhaust in nine California homes	Building and Environment	122		215-229

Wallace L, W Ott	2011	Personal exposure to ultrafine particles	Journal of Exposure Science and Environmental Epidemiology	21	1	20-30
Zhu YF, WC Hinds, M Krudysz, T Kuhn, J Froines, C Sioutas	2005	Penetration of freeway ultrafine particles into indoor environments	Journal of Aerosol Science	36	3	303-322

Oceania Number

Australia Number

Morawska L, CR He, J Hitchins, K Mengersen, D Gilbert	2003	Characteristics of particle number and mass concentrations in residential houses in Brisbane, Australia	Atmospheric Environment	37	30	4195-4203

South East Asia Number

Vietnam Number

Quang TN, NT Hue, P Thai, M Mazaheri, L Morawska	2017	Exploratory assessment of indoor and outdoor particle number concentrations in Hanoi households	Science of the Total Environment	599		284-290

Western Europe Number

Finland Number

Hoek G, G Kos, RM Harrison, J de Hartog, K Meliefste, H ten Brink, K	2008	Indoor-outdoor relationships of particle number and mass in four European cities	Atmospheric Environment	42	1	156-169
Hussein T, K Hameri, MSA Heikkinen, M Kulmala	2005	Indoor and outdoor particle size characterization at a family house in Espoo-Finland	Atmospheric Environment	39	20	3697-3709

Wierzbicka A, M Bohgard, JH Pagels, A Dahl, J Londahl, T Hussein, E	2015	Quantification of differences between occupancy and total monitoring periods for better assessment of exposure to particles in indoor environments	Atmospheric Environment	106		419-428

Greece Number

Hoek G, G Kos, RM Harrison, J de Hartog, K Meliefste, H ten Brink, K	2008	Indoor-outdoor relationships of particle number and mass in four European cities	Atmospheric Environment	42	1	156-169

Netherlands Number

Hoek G, G Kos, RM Harrison, J de Hartog, K Meliefste, H ten Brink, K	2008	Indoor-outdoor relationships of particle number and mass in four European cities	Atmospheric Environment	42	1	156-169

Sweden Number

Wierzbicka A, M Bohgard, JH Pagels, A Dahl, J Londahl, T Hussein, E	2015	Quantification of differences between occupancy and total monitoring periods for better assessment of exposure to particles in indoor environments	Atmospheric Environment	106		419-428

UK Number

Hoek G, G Kos, RM Harrison, J de Hartog, K Meliefste, H ten Brink, K	2008	Indoor-outdoor relationships of particle number and mass in four European cities	Atmospheric Environment	42	1	156-169
Nasir ZA, I Colbeck	2013	Particulate pollution in different housing types in a UK suburban location	Science of the Total Environment	445		165-176

PM>1

Oceania PM>1

Australia PM>1

Hargreaves M, S Parappukkaran, L Morawska, J Hitchins, CR	2003	A pilot investigation into associations between indoor airborne fungal and non-biological particle concentrations in residential houses in Brisbane, Australia	Science of the Total Environment	312		89-101
He C, H Salonen, X Ling, L Crilley, N Jayasundara, HC Cheung, M Hargreaves, F	2014	The impact of flood and post-flood cleaning on airborne microbiological and particle contamination in residential houses	Environment International	69		43725

PM>4

North America PM>4

USA PM>4

Abraham ME	1999	Microanalysis of indoor aerosols and the impact of a Compact High-Efficiency Particulate air (HEPA) filter system	Indoor Air-International Journal of Indoor Air Quality and Climate	9	1	33-40

PM0.1

East Asia PM0.1

Taiwan PM0.1

Li CS, WH Lin, FT Jenq	1993	SIZE DISTRIBUTIONS OF SUBMICROMETER AEROSOLS FROM COOKING	Environment International	19	2	147-154

Lin LY, IJ Liu, HC Chuang, HY Lin, KJ Chuang	2013	Size and composition effects of household particles on inflammation and endothelial dysfunction of human coronary artery endothelial cells	Atmospheric Environment	77		490-495

Eastern Europe PM0.1

Czechia PM0.1

Hussein T, T Glytsos, J Ondracek, P Dohanyosova, V Zdimal, K Hameri, M	2006	Particle size characterization and emission rates during indoor activities in a house	Atmospheric Environment	40	23	4285-4307

North America PM0.1

Canada PM0.1

Evans GJ, A Peers, K Sabaliauskas	2008	Particle dose estimation from frying in residential settings	Indoor Air	18	6	499-510
Ghoshdastidar AJ, Z Hu, Y Nazarenko, PA Ariya	2018	Exposure to nanoscale and microscale particulate air pollution prior to mining development near a northern indigenous community in Quebec, Canada	Environ Sci Pollut Res Int	25	9	8976-8988

USA PM0.1

McAuley TR, R Fisher, X Zhou, PA Jaques, AR Ferro	2010	Relationships of outdoor and indoor ultrafine particles at residences downwind of a major international border crossing in Buffalo, NY	Indoor Air	20	4	298-308

Western Europe PM0.1

Spain PM0.1

Panella P, M Casas, D Donaire-Gonzalez, R Garcia-Esteban, O	2017	Ultrafine particles and black carbon personal exposures in asthmatic and non-asthmatic children at school age	Indoor Air	27	5	891-899

Switzerland PM0.1

Meier R, M Eeftens, HC Phuleria, A Ineichen, E Corradi, M Davey, M Fierz,	2015	Differences in indoor versus outdoor concentrations of ultrafine particles, PM2.5, PMabsorbance and NO2 in Swiss homes	J Expo Sci Environ Epidemiol	25	5	499-505

PM0.1-1

East Asia PM0.1-1

Taiwan PM0.1-1

Lin LY, IJ Liu, HC Chuang, HY Lin, KJ Chuang	2013	Size and composition effects of household particles on inflammation and endothelial dysfunction of human coronary artery endothelial cells	Atmospheric Environment	77		490-495

PM0.25

North America PM0.25

Canada PM0.25

Ghoshdastidar AJ, Z Hu, Y Nazarenko, PA Ariya	2018	Exposure to nanoscale and microscale particulate air pollution prior to mining development near a northern indigenous community in Quebec, Canada	Environ Sci Pollut Res Int	25	9	8976-8988

	USA	PM0.25				
Arhami M, A Polidori, RJ Delfino, T Tjoa, C Sioutas	2009	Associations between Personal, Indoor, and Residential Outdoor Pollutant Concentrations: Implications for Exposure Assessment to Size-Fractionated Particulate Matter	Journal of the Air & Waste Management Association	59	4	392-404
Delfino RJ, N Staimer, T Tjoa, A Polidori, M Arhami, DL Gillen, MT Kleinman, ND	2008	Circulating biomarkers of inflammation, antioxidant activity, and platelet activation are associated with primary combustion aerosols in subjects with coronary artery disease	Environmental Health Perspectives	116	7	898-906

South Asia PM0.25

	India	PM0.25				
Massey D, J Masih, A Kulshrestha, M Habil, A Teneja	2009	Indoor/outdoor relationship of fine particles less than 2.5 mu m (PM2.5) in residential homes locations in central Indian region	Building and Environment	44	10	2037-2045

Western Europe PM0.25

	Italy	PM0.25				
Cattaneo A, C Peruzzo, G Garramone, P Urso, R Ruggeri, P Carrer, DM	2011	Airborne particulate matter and gaseous air pollutants in residential structures in Lodi province, Italy	Indoor Air	21	6	489-500

PM0.25-2.5

North America PM0.25-2.5

USA PM0.25-2.5

Arhami M, A Polidori, RJ Delfino, T Tjoa, C Sioutas	2009	Associations between Personal, Indoor, and Residential Outdoor Pollutant Concentrations: Implications for Exposure Assessment to Size-Fractionated Particulate Matter	Journal of the Air & Waste Management Association	59	4	392-404
Delfino RJ, N Staimer, T Tjoa, A Polidori, M Arhami, DL Gillen, MT Kleinman, ND	2008	Circulating biomarkers of inflammation, antioxidant activity, and platelet activation are associated with primary combustion aerosols in subjects with coronary artery disease	Environmental Health Perspectives	116	7	898-906
Hughes SC, Bellettiere J, Nguyen B, Liles S, Klepeis NE, Quintana PJE, et al.	2018	Randomized Trial to Reduce Air Particle Levels in Homes of Smokers and Children.	American Journal of Preventive Medicine	54	3	359-67
Shrestha PM, Humphrey JL, Carlton EJ, Adgate JL, Barton KE, Root ED, et al.	2019	Impact of Outdoor Air Pollution on Indoor Air Quality in Low-Income Homes during Wildfire Seasons.	International Journal of Environmental Research and Public Health	16	19	

South East Asia PM0.25-2.5

Singapore PM0.25-2.5

Sharm R, R Balasubramanian	2018	Size-fractionated Particulate Matter in Indoor and Outdoor Environments during the 2015 Haze in Singapore: Potential Human Health Risk Assessment	Aerosol and Air Quality Research	18	4	904-917
Zhou J, AL Chen, QL Cao, B Yang, VWC Chang, W Nazaroff	2015	Particle exposure during the 2013 haze in Singapore: Importance of the built environment	Building and Environment	93		14-23

PM0.5

East Asia PM0.5

China PM0.5

Hu JH, NP Li, H Yoshino, U Yanagi, K Hasegawa, N Kagi, YD He, XQ Wei	2017	Field study on indoor health risk factors in households with schoolchildren in south-central China	Building and Environment	117		260-273
Hu YJ, LJ Bao, CL Huang, SM Li, P Liu, EY Zeng	2018	Exposure to air particulate matter with a case study in Guangzhou: Is indoor environment a safe haven in China?	Atmospheric Environment	191		351-359

Taiwan PM0.5

Yu KP, KR Yang, YC Chen, JY Gong, YP Chen, HC Shih, SCC Lung	2015	Indoor air pollution from gas cooking in five Taiwanese families	Building and Environment	93		258-266

Eastern Europe PM0.5

Czechia PM0.5

Hussein T, T Glytsos, J Ondracek, P Dohanyosova, V Zdimal, K Hameri, M	2006	Particle size characterization and emission rates during indoor activities in a house	Atmospheric Environment	40	23	4285-4307
Lazaridis M, K Eleftheriadis, V Zdimal, J Schwarz, Z Wagner, J Ondracek, Y	2017	Number Concentrations and Modal Structure of Indoor/Outdoor Fine Particles in Four European Cities	Aerosol and Air Quality Research	17	1	131-146

North America PM0.5

USA PM0.5

Brown, D.R., Alderman, N., Weinberger, B., Lewis, C., Bradley, J. and Curtis, L.	2014	Outdoor wood furnaces create significant indoor particulate pollution in neighboring homes	Inhalation toxicology	26	10	628-635
Pickett AR, ML Bell	2011	Assessment of Indoor Air Pollution in Homes with Infants	International Journal of Environmental Research and Public Health	8	12	4502-4520
Wallace L	2005	Ultrafine particles from a vented gas clothes dryer	Atmospheric Environment	39	32	5777-5786

South Asia PM0.5

India PM0.5

Massey D, J Masih, A Kulshrestha, M Habil, A Teneja	2009	Indoor/outdoor relationship of fine particles less than 2.5 mu m (PM2.5) in residential homes locations in central Indian region	Building and Environment	44	10	2037-2045

West Asia & North Africa PM0.5

Iran PM0.5

Mirhoseini SH, M Nikaeen, K Satoh, K Makimura	2016	Assessment of Airborne Particles in Indoor Environments: Applicability of Particle Counting for Prediction of Bioaerosol Concentrations	Aerosol and Air Quality Research	16	8	1903-1910

Western Europe PM0.5

Greece PM0.5

Lazaridis M, K Eleftheriadis, V Zdimal, J Schwarz, Z Wagner, J Ondracek, Y	2017	Number Concentrations and Modal Structure of Indoor/Outdoor Fine Particles in Four European Cities	Aerosol and Air Quality Research	17	1	131-146

Saraga DE, T Maggos, DA Missia, EI Tolis, C Vasilakos, JG Bartzis	2010	SECONDARY ORGANIC PARTICLES FORMATION FROM OZONE-TERPENES REACTION: A CASE STUDY IN A RESIDENCE OF A MEDITERRANEAN CITY	Fresenius Environmental Bulletin	19		2019-2025

Italy PM0.5

Cattaneo A, C Peruzzo, G Garramone, P Urso, R Ruggeri, P Carrer, DM	2011	Airborne particulate matter and gaseous air pollutants in residential structures in Lodi province, Italy	Indoor Air	21	6	489-500

Portugal PM0.5

Almeida-Silva M, Pilou M, Housiadas C, Almeida SM.	2018	Internal dose of particles in the elderly—modeling based on aerosol measurements.	Environmental Science and Pollution Research	25	24	23645-56
Gomes JFP, JCM Bordado, PCS Albuquerque	2012	Monitoring exposure to airborne ultrafine particles in Lisbon, Portugal	Inhalation Toxicology	24	7	425-433

PM0.5-1

Western Europe PM0.5-1

Portugal PM0.5-1

Gomes JFP, JCM Bordado, PCS Albuquerque	2012	Monitoring exposure to airborne ultrafine particles in Lisbon, Portugal	Inhalation Toxicology	24	7	425-433

PM0.5-2.5

North America PM0.5-2.5

USA PM0.5-2.5

Klepeis, N.E., Bellettiere, J., Hughes, S.C., Nguyen, B., Berardi, V., Liles, S.,	2017	Fine particles in homes of predominantly low-income families with children and smokers: Key physical and behavioral determinants to inform indoor-air-quality interventions	PloS one	12	5	e0177718

PM1

Africa (except North) PM1

Ethiopia PM1

Embiale A, Zewge F, Chandravanshi BS, Sahle-Demessie E.	2019	Short-term exposure assessment to particulate matter and total volatile organic compounds in indoor air during cooking Ethiopian sauces (Wot) using electricity, kerosene and charcoal fuels.	Indoor and Built Environment	28	8	1140-54

East Asia PM1

China PM1

Cai J, Yu W, Li BZ, Yao RM, Zhang TJW, Guo M, et al.	2019	Particle removal efficiency of a household portable air cleaner in real-world residences: A single-blind cross-over field study.	Energy and Buildings	203		
Hu YJ, LJ Bao, CL Huang, SM Li, P Liu, EY Zeng	2018	Exposure to air particulate matter with a case study in Guangzhou: Is indoor environment a safe haven in China?	Atmospheric Environment	191		351-359

Huang C, X Wang, W Liu, J Cai, L Shen, Z Zou, R Lu, J Chang, X Wei, C Sun, Z	2016	Household indoor air quality and its associations with childhood asthma in Shanghai, China: On-site inspected methods and preliminary results	Environ Res	151		154-167
Liu ZJ, AG Li, ZP Hu, HF Sun	2014	Study on the potential relationships between indoor culturable fungi, particle load and children respiratory health in Xi'an, China	Building and Environment	80		105-114
Niu X, B Guinot, J Cao, H Xu, J Sun	2015	Particle size distribution and air pollution patterns in three urban environments in Xi'an, China	Environ Geochem Health	37	5	801-12
Zhu SW, W Cai, H Yoshino, U Yanagi, K Hasegawa, N Kagi, MQ Chen	2015	Primary pollutants in schoolchildren's homes in Wuhan, China	Building and Environment	93		41-53
	Taiwan	PM1				
Li CS, CH Lin	2003	Carbon profile of residential indoor PM1 and PM2.5 in the subtropical region	Atmospheric Environment	37	7	881-888
Li CS, WH Lin, FT Jenq	1993	SIZE DISTRIBUTIONS OF SUBMICROMETER AEROSOLS FROM COOKING	Environment International	19	2	147-154
Yen YC, Yang CY, Mena KD, Cheng YT, Yuan CS, Chen PS.	2019	Jumping on the bed and associated increases of PM10, PM2.5, PM1, airborne endotoxin, bacteria, and fungi concentrations.	Environ Pollut	245		799-809
Yu KP, KR Yang, YC Chen, JY Gong, YP Chen, HC Shih, SCC Lung	2015	Indoor air pollution from gas cooking in five Taiwanese families	Building and Environment	93		258-266

Eastern Europe PM1

Czechia PM1

Braniš, M., Hovorka, J., Řezáčcová, P., Domasová, M., & Lazaridis, M.	2005	Effect of Indoor and Outdoor Sources on Particulate Matter Concentration in a Naturally Ventilated Flat (URBAN-AEROSOL Project-Prague)	Indoor Built Environment	14	3	307-12
Talbot N, L Kubelova, O Makes, J Ondracek, M Cusack, J Schwarz, P	2017	Transformations of Aerosol Particles from an Outdoor to Indoor Environment	Aerosol and Air Quality Research	17	3	653-665

Lithuania PM1

Prasauskas T, D Martuzevicius, E Krugly, D Ciuzas, I Stasiulaitiene, R	2014	Spatial and temporal variations of particulate matter concentrations in multifamily apartment buildings	Building and Environment	76		43755

North America PM1

Canada PM1

Ghoshdastidar AJ, Z Hu, Y Nazarenko, PA Ariya	2018	Exposure to nanoscale and microscale particulate air pollution prior to mining development near a northern indigenous community in Quebec, Canada	Environ Sci Pollut Res Int	25	9	8976-8988
Kearney J, L Wallace, M MacNeill, ME Heroux, W Kindzierski, A Wheeler	2014	Residential infiltration of fine and ultrafine particles in Edmonton	Atmospheric Environment	94		793-805
Weichenthal S, G Mallach, R Kulka, A Black, A Wheeler, H You, M St-Jean,	2013	A randomized double-blind crossover study of indoor air filtration and acute changes in cardiorespiratory health in a First Nations community	Indoor Air	23	3	175-184

USA PM1

Du L, S Batterman, E Parker, C Godwin, JY Chin, A O'Toole, T Robins, W	2011	Particle concentrations and effectiveness of free-standing air filters in bedrooms of children with asthma in Detroit, Michigan	Building and Environment	46	11	2303-2313
Hart JF, TJ Ward, TM Spear, RJ Rossi, NN Holland, BG Loushin	2011	Evaluating the Effectiveness of a Commercial Portable Air Purifier in Homes with Wood Burning Stoves: A Preliminary Study	Journal of Environmental and Public Health			
Koehler K, Good N, Wilson A, Molter A, Moore BF, Carpenter T, et al.	2019	The Fort Collins commuter study: Variability in personal exposure to air pollutants by microenvironment.	Indoor Air: Interational Journal of Indoor Environment and Health	29	2	231-41
Levy JI, T Dumyahn, JD Spengler	2002	Particulate matter and polycyclic aromatic hydrocarbon concentrations in indoor and outdoor microenvironments in Boston, Massachusetts	Journal of Exposure Analysis and Environmental Epidemiology	12	2	104-114
Patton AP, L Calderon, YY Xiong, ZC Wang, J Senick, MS Allacci, D Plotnik, R	2016	Airborne Particulate Matter in Two Multi-Family Green Buildings: Concentrations and Effect of Ventilation and Occupant Behavior	International Journal of Environmental Research and Public Health	13	1	
Wallace L	2006	Indoor sources of ultrafine and accumulation mode particles: Size distributions, size-resolved concentrations, and source strengths	Aerosol Science and Technology	40	5	348-360

Oceania PM1

Australia PM1

Cheong CD, HG Neumeister-Kemp, PW Dingle, GS Hardy	2004	Intervention study of airborne fungal spora in homes with portable HEPA filtration units	Journal of Environmental Monitoring	6	11	866-873

Hargreaves M, S Parappukkaran, L Morawska, J Hitchins, CR	2003	A pilot investigation into associations between indoor airborne fungal and non-biological particle concentrations in residential houses in Brisbane, Australia	Science of the Total Environment	312		89-101

South America PM1

Brazil PM1

Segalin B, P Kumar, K Micadei, A Fornaro, FLT Goncalves	2017	Size-segregated particulate matter inside residences of elderly in the Metropolitan Area of Sao Paulo, Brazil	Atmospheric Environment	148		139-151

South Asia PM1

Bangladesh PM1

Akther T, Ahmed M, Shohel M, Ferdousi FK, Salam A.	2019	Particulate matters and gaseous pollutants in indoor environment and Association of ultra-fine particulate matters (PM1) with lung function.		26	6	5475-84.

India PM1

Gupta S, A Srivastava, VK Jain	2008	Particle size distribution of aerosols and associated heavy metals in kitchen environments	Environ Monit Assess	142		141-8
Kulshreshtha P, M Khare, P Seetharaman	2008	Indoor air quality assessment in and around urban slums of Delhi city, India	Indoor Air	18	6	488-98
Massey D, A Kulshrestha, J Masih, A Taneja	2012	Seasonal trends of PM10, PM5.0, PM2.5 & PM1.0 in indoor and outdoor environments of residential homes located in North-Central India	Building and Environment	47		223-231

Massey D, J Masih, A Kulshrestha, M Habil, A Teneja	2009	Indoor/outdoor relationship of fine particles less than 2.5 mu m (PM2.5) in residential homes locations in central Indian region	Building and Environment	44	10	2037-2045
Srivastava A, S Gupta, VK Jain	2009	Winter-time size distribution and source apportionment of total suspended particulate matter and associated metals in Delhi	Atmospheric Research	92	1	88-99

Pakistan PM1

Ali Z, F Naz, ZA Nasir, S Sidra, I Colbeck	2015	PARTICULATE POLLUTION IN URBAN RESIDENTIAL BUILT ENVIRONMENTS DURING WINTER AND SUMMER SEASONS IN LAHORE, PAKISTAN	Journal of Animal and Plant Sciences	25	3	660-665
Nasir, Z. A., Colbeck, I., Ali, Z., & Ahmad, S	2013	Indoor particulate matter in developing countries: a case study in Pakistan and potential intervention strategies	Environmental Research Letters	8	2	024002

West Asia & North Africa PM1

Iran PM1

Hassanvand MS, K Naddafi, H Kashani, S Faridi, N Kunzli, R Nabizadeh, F	2017	Short-term effects of particle size fractions on circulating biomarkers of inflammation in a panel of elderly subjects and healthy young adults	Environmental Pollution	223		695-704
Hassanvand MS, K Naddafi, S Faridi, M Arhami, R Nabizadeh, MH Sowlat, Z	2014	Indoor/outdoor relationships of PM10, PM2.5, and PM1 mass concentrations and their water-soluble ions in a retirement home and a school dormitory	Atmospheric Environment	82		375-382
Mirhoseini SH, M Nikaeen, K Satoh, K Makimura	2016	Assessment of Airborne Particles in Indoor Environments: Applicability of Particle Counting for Prediction of Bioaerosol Concentrations	Aerosol and Air Quality Research	16	8	1903-1910

Israel PM1

Jodeh S, AR Hasan, J Amarah, F Judeh, R Salghi, H Lgaz, W Jodeh	2018	Indoor and outdoor air quality analysis for the city of Nablus in Palestine: seasonal trends of PM10, PM5.0, PM2.5, and PM1.0 of residential homes	Air Quality Atmosphere and Health	11	2	229-237

Western Europe PM1

Belgium PM1

Buczynska AJ, A Krata, R Van Grieken, A Brown, G Polezer, K De Wael, S	2014	Composition of PM2.5 and PM1 on high and low pollution event days and its relation to indoor air quality in a home for the elderly	Science of the Total Environment	490		134-143
Stranger M, SS Potgieter-Vermaak, R Van Grieken	2009	Particulate matter and gaseous pollutants in residences in Antwerp, Belgium	Science of the Total Environment	407	3	1182-1192

Finland PM1

Sippula O, H Rintala, M Happo, P Jalava, K Kuuspalo, A Viren, A	2013	Characterization of Chemical and Microbial Species from Size-Segregated Indoor and Outdoor Particulate Samples	Aerosol and Air Quality Research	13	4	1212-U343
Toivola M, A Nevalainen, S Alm	2004	Personal exposures to particles and microbes in relation to microenvironmental concentrations	Indoor Air	14	5	351-359

France PM1

Maesano CN, Caillaud D, Youssouf H, Banerjee S, Prud'Homme J, Audi C, et	2019	Indoor exposure to particulate matter and volatile organic compounds in dwellings and workplaces and respiratory health in French farmers.	Multidisciplinary Respiratory Medicine	14	1	eCollection

	Germany	PM1				
Franck U, O Herbarth, S Roder, U Schlink, M Borte, U Diez, U Kramer, I Lehmann	2011	Respiratory effects of indoor particles in young children are size dependent	Science of the Total Environment	409	9	1621-1631
	Greece	PM1				
Assimakopoulos VD, T Bekiari, S Pateraki, T Maggos, P Stamatis, P	2018	Assessing personal exposure to PM using data from an integrated indoor-outdoor experiment in Athens-Greece	Science of the Total Environment	636		1303-1320
Saraga DE, T Maggos, CG Helmis, J Michopoulos, JG Bartzis, C Vasilakos	2010	PM1 and PM2.5 ionic composition and VOCs measurements in two typical apartments in Athens, Greece: investigation of smoking contribution to indoor air concentrations	Environmental Monitoring and Assessment	167		321-331
Stamatelopoulou A, Asimakopoulos DN, Maggos T.	2019	Effects of PM, TVOCs and comfort parameters on indoor air quality of residences with young children.	Building and Environment	150		233-44
	Italy	PM1				
Cattaneo A, C Peruzzo, G Garramone, P Urso, R Ruggeri, P Carrer, DM	2011	Airborne particulate matter and gaseous air pollutants in residential structures in Lodi province, Italy	Indoor Air	21	6	489-500
	Portugal	PM1				
Almeida-Silva M, Pilou M, Housiadas C, Almeida SM.	2018	Internal dose of particles in the elderly—modeling based on aerosol measurements.	Environmental Science and Pollution Research	25	24	23645-56
Almeida-Silva, M., Wolterbeek, H.T. and Almeida, S.M	2014	Elderly exposure to indoor air pollutants	Atmospheric Environment	85		54-63

Miguel AF, AH Reis, M Melgao	2009	Urban indoor-outdoor aerosol measurements in Portugal and the global warming scenario	International Journal of Global Warming	1		356-367
Slezakova K, C Texeira, S Morais, MD Pereira	2015	CHILDREN'S INDOOR EXPOSURES TO (ULTRA) FINE PARTICLES IN AN URBAN AREA: COMPARISON BETWEEN SCHOOL AND HOME ENVIRONMENTS	Journal of Toxicology and Environmental Health-Part a-Current Issues	78		886-896
	Sweden	PM1				
Isaxon C, A Gudmundsson, EZ Nordin, L Lonnblad, A Dahl, G Wieslander, M	2015	Contribution of indoor-generated particles to residential exposure	Atmospheric Environment	106		458-466
Johannesson S, P Gustafson, P Molnar, L Barregard, G Sallsten	2007	Exposure to fine particles (PM2.5 and PM1) and black smoke in the general population: personal, indoor, and outdoor levels	Journal of Exposure Science and Environmental Epidemiology	17	7	613-624
	UK	PM1				
Jones NC, CA Thornton, D Mark, RM Harrison	2000	Indoor/outdoor relationships of particulate matter in domestic homes with roadside, urban and rural locations	Atmospheric Environment	34	16	2603-2612
Nasir ZA, I Colbeck	2013	Particulate pollution in different housing types in a UK suburban location	Science of the Total Environment	445		165-176

PM10

Africa (except North) PM10

Ethiopia PM10

Embiale A, Zewge F, Chandravanshi BS, Sahle-Demessie E.	2019	Short-term exposure assessment to particulate matter and total volatile organic compounds in indoor air during cooking Ethiopian sauces (Wot) using electricity, kerosene and charcoal fuels.	Indoor and Built Environment	28	8	1140-54

Nigeria PM10

Ibhafidon LI, DO Obaseki, GE Erhabor, AA Akor, I Irabor, I Obioh	2014	Respiratory symptoms, lung function and particulate matter pollution in residential indoor environment in Ile-Ife, Nigeria	Niger Med J	55	1	48-53

South Africa PM10

Vanker A, Nduru PM, Barnett W, Dube FS, Sly PD, Gie RP, et al.	2019	Indoor air pollution and tobacco smoke exposure: impact on nasopharyngeal bacterial carriage in mothers and infants in an African birth cohort study.	ERJ Open Research	5	1	00052-2018
Vanker A, W Barnett, PM Nduru, RP Gie, PD Sly, HJ Zar	2015	Home environment and indoor air pollution exposure in an African birth cohort study	Science of the Total Environment	536		362-367

Central America and Caribbean PM10

Mexico PM10

Cortez-Lugo M, H Moreno-Macias, F Holguin-Molina, JC Chow, JG Watson, V	2008	Relationship between indoor, outdoor, and personal fine particle concentrations for individuals with COPD and predictors of indoor-outdoor ratio in Mexico city	Journal of Exposure Science and Environmental Epidemiology	18	1	109-115

East Asia PM10

China PM10

Ai ZT, CM Mak, DJ Cui	2015	On-site measurements of ventilation performance and indoor air quality in naturally ventilated high-rise residential buildings in Hong Kong	Indoor and Built Environment	24	2	214-224
Cai J, Yu W, Li BZ, Yao RM, Zhang TJW, Guo M, et al.	2019	Particle removal efficiency of a household portable air cleaner in real-world residences: A single-blind cross-over field study.	Energy and Buildings	203		
Chao CY, EC Cheng	2002	Source apportionment of indoor PM(2.5) and PM(10) in homes	Indoor and Built Environment	11	1	27-37
Chao CY, KK Wong	2002	Residential indoor PM10 and PM2.5 in Hong kong and the elemental composition	Atmospheric Environment	36	2	265-277
Chao CY, KK Wong, EC Cheng	2002	Size distribution of indoor particulate matter in 60 homes in Hong Kong	Indoor and Built Environment	11	1	18-26
Chao CYH, TCW Tung, J Burnett	1998	Influence of different indoor activities on the indoor particulate levels in residential buildings	Indoor and Built Environment	7	2	110-121
Cheng YL, M Yan, JL Li, ZR Liu, YH Bai, W Tian, DG Wu, Q Cheng	2006	Variations in indoor PM10 concentrations in sixteen homes in Guiyang City, People's Republic of China	Bulletin of Environmental Contamination and Toxicology	77	1	112-118
Cheung PK, Jim CY.	2019	Indoor air quality in substandard housing in Hong Kong.	Sustainable Cities and Society	48		101583
Hu YJ, LJ Bao, CL Huang, SM Li, P Liu, EY Zeng	2018	Exposure to air particulate matter with a case study in Guangzhou: Is indoor environment a safe haven in China?	Atmospheric Environment	191		351-359

Huang C, X Wang, W Liu, J Cai, L Shen, Z Zou, R Lu, J Chang, X Wei, C Sun, Z	2016	Household indoor air quality and its associations with childhood asthma in Shanghai, China: On-site inspected methods and preliminary results	Environ Res	151		154-167
Lee SC, M Chang, KY Chan	1999	Indoor and outdoor air quality investigation at six residential buildings in Hong Kong	Environment International	25	4	489-496
Lee SC, WM Li, CH Ao	2002	Investigation of indoor air quality at residential homes in Hong Kong - case study	Atmospheric Environment	36	2	225-237
Liu J, Y Man, Y Liu	2014	Temporal variability of PM10 and PM2.5 inside and outside a residential home during 2014 Chinese Spring Festival in Zhengzhou, China	Natural Hazards	73	3	2149-2154
Liu Z, K Cheng, H Li, G Cao, D Wu, Y Shi	2018	Exploring the potential relationship between indoor air quality and the concentration of airborne culturable fungi: a combined experimental and neural network modeling study	Environ Sci Pollut Res Int	25	4	3510-3517
Liu ZJ, AG Li, ZP Hu, HF Sun	2014	Study on the potential relationships between indoor culturable fungi, particle load and children respiratory health in Xi'an, China	Building and Environment	80		105-114
Niu X, B Guinot, J Cao, H Xu, J Sun	2015	Particle size distribution and air pollution patterns in three urban environments in Xi'an, China	Environ Geochem Health	37	5	801-12
Tian LW, GQ Zhang, Q Zhang, DJ Moschandreas, JH Hao, JP Lin, YH Liu	2008	The impact of kitchen activities on indoor pollutant concentrations	Indoor and Built Environment	17	4	377-383
Tung TCW, CYH Chao, J Burnett, SW Pang, RYM Lee	1999	A territory wide survey on indoor particulate level in Hong Kong	Building and Environment	34	2	213-220

Wang JN, Y Zhang	1990	CO and particle pollution of indoor air in Beijing and its elemental analysis	Biomed Environ Sci	3	2	132-8
Wang ZJ, QW Xue, YC Ji, ZY Yu	2018	Indoor environment quality in a low-energy residential building in winter in Harbin	Building and Environment	135		194-201
Wu YJ, Li GY, Yang Y, An TC.	2019	Pollution evaluation and health risk assessment of airborne toxic metals in both indoors and outdoors of the Pearl River Delta, China.	Environmental Research	179		Epub
Zhang HB, JC Xie, H Yoshino, U Yanagi, K Hasegawa, N Kagi, ZW Lian	2016	Thermal and environmental conditions in Shanghai households: Risk factors for childhood health	Building and Environment	104		35-46
Zhao HY, LY Shao, Q Yao	2005	Microscopic morphology and size distribution of residential indoor PM10 in Beijing City	Indoor and Built Environment	14	6	513-520
Zhao WC, JP Cheng, ZY Yu, QL Tang, F Cheng, YW Yin, WH Wang	2013	Levels, seasonal variations, and health risks assessment of ambient air pollutants in the residential areas	International Journal of Environmental Science and Technology	10	3	487-494
Zhou J, B Han, ZP Bai, Y You, JF Zhang, C Niu, YT Liu, N Zhang, F He, X Ding, B	2012	Particle Exposure Assessment for Community Elderly (PEACE) in Tianjin, China: Mass concentration relationships	Atmospheric Environment	49		77-84
Zhu SW, W Cai, H Yoshino, U Yanagi, K Hasegawa, N Kagi, MQ Chen	2015	Primary pollutants in schoolchildren's homes in Wuhan, China	Building and Environment	93		41-53

	Japan	PM10				
Funasaka K, T Miyazaki, K Tsuruho, K Tamura, T Mizuno, K Kuroda	2000	Relationship between indoor and outdoor carbonaceous particulates in roadside households	Environmental Pollution	110	1	127-134
Kang Y, K Nagano	2016	Field measurement of indoor air quality and airborne microbes in a near-zero energy house with an earth tube in the cold region of Japan	Science and Technology for the Built Environment	22	7	1010-1023
Michikawa T, S Nakai, H Nitta, K Tamura	2014	Validity of using annual mean particulate matter concentrations as measured at fixed site in assessing personal exposure: An exposure assessment study in Japan	Science of the Total Environment	466		673-680
Nakai S, H Nitta, M Ono, K Abe, M Sakaguchi	1999	Measurements of biological contaminants and particulate matter inside a dwelling in Japan	Indoor Air	9	1	41-46
Sugiyama T, T Amagai, H Matsushita, M Soma	1999	Size distribution of indoor airborne particulates collected by a low-flow rate cascade impactor	Indoor and Built Environment	8	6	361-369
	South Korea	PM10				
Byun H, H Bae, D Kim, H Shin, C Yoon	2010	Effects of socioeconomic factors and human activities on children's PM10 exposure in inner-city households in Korea	International Archives of Occupational and Environmental Health	83	8	867-878
Hwang Y, K Lee	2018	Contribution of microenvironments to personal exposures to PM10 and PM2.5 in summer and winter	Atmospheric Environment	175		192-198
Jo WK, JY Lee	2006	Indoor and outdoor levels of respirable particulates (PM10) and Carbon Monoxide (CO) in high-rise apartment buildings	Atmospheric Environment	40	32	6067-6076

Kang K, Kim H, Kim DD, Lee YG, Kim T.	2019	Characteristics of cooking-generated PM10 and PM2.5 in residential buildings with different cooking and ventilation types.	Science of The Total Environment	668		56-66
Lee CH, BK Lee, IB Oh, JH Lee, CS Sim, Y Kim	2012	Indoor Air Quality in Elementary School Children's Homes in Ulsan: Comparison between Groups with and without Allergic Rhinitis	Journal of Korean Society for Atmospheric Environment	28	4	365-373
Lee JH, HS Lee, MR Park, SW Lee, EH Kim, JB Cho, J Kim, Y Han, K Jung, HK	2014	Relationship Between Indoor Air Pollutant Levels and Residential Environment in Children With Atopic Dermatitis	Allergy Asthma & Immunology Research	6	6	517-524
Lee JY, C Kim, J Kim, SH Ryu, GN Bae	2018	Exposure Assessments for Children in Homes and in Daycare Centers to NO2, PMs and Black Carbon	Asian Journal of Atmospheric Environment	12	3	204-214
Oh HJ, Jeong NN, Sohn JR, Kim J.	2019	Personal exposure to indoor aerosols as actual concern: Perceived indoor and outdoor air quality, and health performances.	Building and Environment	165		106403
Seo SC, IS Kang, SG Lim, JT Choung, Y Yoo	2015	Indoor air pollutants and atopic dermatitis in socioeconomically disadvantaged children	Allergy Asthma & Respiratory Disease	3	3	206-212
	Taiwan	PM10				
Chi, M.C., Guo, S.E., Hwang, S.L., Chou, C.T., Lin, C.M. and Lin, Y.C	2017	Exposure to Indoor Particulate MatterWorsens the Symptoms and Acute Exacerbations in Chronic Obstructive Pulmonary Disease Patients of Southwestern Taiwan: A Pilot Study	International journal of environmental research and public health	14	1	4
Li CS	1994	ELEMENTAL COMPOSITION OF RESIDENTIAL INDOOR PM10 IN THE URBAN ATMOSPHERE OF TAIPEI	Atmospheric Environment	28	19	3139-3144

Li CS	1994	RELATIONSHIPS OF INDOOR OUTDOOR INHALABLE AND RESPIRABLE PARTICLES IN DOMESTIC ENVIRONMENTS	Science of the Total Environment	151	3	205-211
Lin LY, CY Lin, YC Lin, KJ Chuang	2009	The effects of indoor particles on blood pressure and heart rate among young adults in Taipei, Taiwan	Indoor Air	19	6	482-488
Lin LY, HC Chuang, IJ Liu, HW Chen, KJ Chuang	2013	Reducing indoor air pollution by air conditioning is associated with improvements in cardiovascular health among the general population	Science of the Total Environment	463		176-181
Lung, S.C.C., Mao, I.F. and Liu, L.J.S.	2007	Residents' particle exposures in six different communities in Taiwan	Science of the total environment	377	1	81-92
Yen YC, Yang CY, Mena KD, Cheng YT, Yuan CS, Chen PS.	2019	Jumping on the bed and associated increases of PM10, PM2.5, PM1, airborne endotoxin, bacteria, and fungi concentrations.	Environ Pollut	245		799-809
Yu KP, KR Yang, YC Chen, JY Gong, YP Chen, HC Shih, SCC Lung	2015	Indoor air pollution from gas cooking in five Taiwanese families	Building and Environment	93		258-266

Eastern Europe PM10

Czechia PM10

Braniš, M., Hovorka, J., Řezáčcová, P., Domasová, M., & Lazaridis, M.	2005	Effect of Indoor and Outdoor Sources on Particulate Matter Concentration in a Naturally Ventilated Flat (URBAN-AEROSOL Project-Prague)	Indoor Built Environment	14	3	307-12
Okonski K, C Degrendele, L Melymuk, L Landlova, P Kukucka, S Vojta, J	2014	Particle Size Distribution of Halogenated Flame Retardants and Implications for Atmospheric Deposition and Transport	Environmental Science & Technology	48	24	14426-

		Lithuania	PM10			
Du L, T Prasauskas, V Leivo, M Turunen, M Pekkonen, M Kiviste, A Aaltonen, D	2015	Assessment of indoor environmental quality in existing multi-family buildings in North-East Europe	Environ Int	79		74-84
Prasauskas T, D Martuzevicius, E Krugly, D Ciuzas, I Stasiulaitiene, R	2014	Spatial and temporal variations of particulate matter concentrations in multifamily apartment buildings	Building and Environment	76		43755
		**North Macedonia PM10**				
Vilcekova S, IZ Apostoloski, L Meciarova, EK Burdova, J Kiselak	2017	Investigation of Indoor Air Quality in Houses of Macedonia	Int J Environ Res Public Health	14	1	
		**Poland PM10**				
Bentayeb M, D Norback, M Bednarek, A Bernard, GH Cai, S Cerrai, KK	2015	Indoor air quality, ventilation and respiratory health in elderly residents Living in nursing homes in Europe	European Respiratory Journal	45	5	1228-1238
Ścibor M, Balcerzak B, Galbarczyk A, Targosz N, Jasienska G.	2019	Are we safe inside? Indoor air quality in relation to outdoor concentration of PM10 and PM2.5 and to characteristics of homes.	Sustainable Cities and Society	48		101537
		**Slovakia PM10**				
Brauer M, F Hruba, E Mihalikova, E Fabianova, P Miskovic, A Plzikova, M	2000	Personal exposure to particles in Banska Bystrica, Slovakia	Journal of Exposure Analysis and Environmental Epidemiology	10	5	478-487
Meciarova L, S Vilcekova, EK Burdova, J Kiselak	2017	Factors Effecting the Total Volatile Organic Compound (TVOC) Concentrations in Slovak Households	International Journal of Environmental Research and Public Health	14	12	

North America PM10

Canada PM10

Akom JB, Sadick AM, Issa MH, Rashwan S, Duhoux M.	2018	The indoor environmental quality performance of green low-income single-family housing.	Journal of Green Building	13	2	98-120
Georghiou PE, P Blagden, DA Snow, L Winsor, DT Williams	1991	MUTAGENICITY OF INDOOR AIR CONTAINING ENVIRONMENTAL TOBACCO-SMOKE - EVALUATION OF A PORTABLE PM-10 IMPACTOR SAMPLER	Environmental Science & Technology	25	8	1496-1500
Ghoshdastidar AJ, Z Hu, Y Nazarenko, PA Ariya	2018	Exposure to nanoscale and microscale particulate air pollution prior to mining development near a northern indigenous community in Quebec, Canada	Environ Sci Pollut Res Int	25	9	8976-8988
Guggisberg M, PA Hessel, D Michaelchuk, M Atiemo	2003	Particulate matter and gaseous contaminants in indoor environments in an isolated northern community	Int J Circumpolar Health	62	2	120-9
Miller JD, Dugandzic R, Frescura AM, Salares V	2007	Indoor-and outdoor-derived contaminants in urban and rural homes in Ottawa, Ontario, Canada	Journal of the Air & Waste Management Association	57	3	297-302
Pellizzari ED, CA Clayton, CE Rodes, RE Mason, LL Piper, B Fort, G Pfeifer, D Lynam	1999	Particulate matter and manganese exposures in Toronto, Canada	Atmospheric Environment	33	5	721-734
Rasmussen PE, C Levesque, M Chenier, HD Gardner	2018	Contribution of metals in resuspended dust to indoor and personal inhalation exposures: Relationships between PM10 and settled dust	Building and Environment	143		513-522
Weichenthal S, G Mallach, R Kulka, A Black, A Wheeler, H You, M St-Jean,	2013	A randomized double-blind crossover study of indoor air filtration and acute changes in cardiorespiratory health in a First Nations community	Indoor Air	23	3	175-184

	USA	PM10				
Abt E, HH Suh, G Allen, P Koutrakis	2000	Characterization of indoor particle sources: A study conducted in the metropolitan Boston area	Environmental Health Perspectives	108	1	35-44
Breysse PN, TJ Buckley, D Williams, CM Beck, SJ Jo, B Merriman, S	2005	Indoor exposures to air pollutants and allergens in the homes of asthmatic children in inner-city Baltimore	Environmental Research	98	2	167-176
Chen Q, LM Hildemann	2009	Size-Resolved Concentrations of Particulate Matter and Bioaerosols Inside versus Outside of Homes	Aerosol Science and Technology	43	7	699-713
Clayton CA, RL Perritt, ED Pellizzari, KW Thomas, RW Whitmore, LA Wallace, H	1993	PARTICLE TOTAL EXPOSURE ASSESSMENT METHODOLOGY (PTEAM) STUDY - DISTRIBUTIONS OF AEROSOL AND ELEMENTAL CONCENTRATIONS IN PERSONAL, INDOOR, AND OUTDOOR AIR SAMPLES IN A SOUTHERN CALIFORNIA COMMUNITY	Journal of Exposure Analysis and Environmental Epidemiology	3	2	227-250
Colome SD, NY Kado, P Jaques, M Kleinman	1992	INDOOR OUTDOOR AIR-POLLUTION RELATIONS - PARTICULATE MATTER LESS THAN 10 MU IN AERODYNAMIC DIAMETER (PM-10) IN HOMES OF ASTHMATICS	Atmospheric Environment Part a-General Topics	26	12	2173-2178
Corsi RL, JA Siegel, C Chiang	2008	Particle resuspension during the use of vacuum cleaners on residential carpet	Journal of Occupational and Environmental Hygiene	5	4	232-238
Delfino RJ, PJ Quintana, J Floro, VM Gastanaga, BS Samimi, MT Kleinman, LJ	2004	Association of FEV1 in asthmatic children with personal and microenvironmental exposure to airborne particulate matter	Environ Health Perspect	112	8	932-41
Diette GB, NN Hansel, TJ Buckley, J Curtin-Brosnan, PA Eggleston, EC Matsui,	2007	Home indoor pollutant exposures among inner-city children with and without asthma	Environmental Health Perspectives	115	11	1665-1669

Doll SC, EL Davison, BR Painting	2016	Weatherization impacts and baseline indoor environmental quality in low income single-family homes	Building and Environment	107		181-190
Eggleston PA, A Butz, C Rand, J Curtin-Brosnan, S Kanchanaraksa, L Swartz, P	2005	Home environmental intervention in inner-city asthma: a randomized controlled clinical trial	Annals of Allergy Asthma & Immunology	95	6	518-524
Evans GF, RV Highsmith, LS Sheldon, JC Suggs, RW Williams, RB Zweidinger, JP	2000	The 1999 Fresno particulate matter exposure studies: Comparison of community, outdoor, and residential PM mass measurements	Journal of the Air & Waste Management Association	50	11	1887-1896
Frey SE, H Destaillats, S Cohn, S Ahrentzen, MP Fraser	2014	Characterization of indoor air quality and resident health in an Arizona senior housing apartment building	Journal of the Air & Waste Management Association	64	11	1251-1259
Frey SE, H Destaillats, S Cohn, S Ahrentzen, MP Fraser	2015	The effects of an energy efficiency retrofit on indoor air quality	Indoor Air	25	2	210-219
Geller MD, MH Chang, C Sioutas, BD Ostro, MJ Lipsett	2002	Indoor/outdoor relationship and chemical composition of fine and coarse particles in the southern California deserts	Atmospheric Environment	36	6	1099-1110
Hsu SI, K Ito, M Kendall, M Lippmann	2012	Factors affecting personal exposure to thoracic and fine particles and their components	Journal of Exposure Science and Environmental Epidemiology	22	5	439-447
Hunt A, JA Crawford, PF Rosenbaum, JL Abraham	2011	Levels of household particulate matter and environmental tobacco smoke exposure in the first year of life for a cohort at risk for asthma in urban Syracuse, NY	Environment International	37	7	1196-1205
Jansen KL, TV Larson, JQ	2005	Associations between health effects and particulate	Environmental Health Perspectives	113	12	1741-1746

Koenig, TF Mar, C Fields, J Stewart, M Lippmann		matter and black carbon in subjects with respiratory disease				
Kamens R, CT Lee, R Wiener, D Leith	1991	A STUDY TO CHARACTERIZE INDOOR PARTICLES IN 3 NONSMOKING HOMES	Atmospheric Environment Part a-General Topics	25		939-948
Keeler GJ, JT Dvonch, FY Yip, EA Parker, BA Israel, FJ Marsik, M Morishita, JA	2002	Assessment of personal and community-level exposures to particulate matter among children with asthma in Detroit, Michigan, as part of Community Action Against Asthma (CAAA)	Environmental Health Perspectives	110		173-181
Leaderer BP, L Naeher, T Jankun, K Balenger, TR Holford, C Toth, J Sullivan,	1999	Indoor, outdoor, and regional summer and winter concentrations of PM10, PM2.5, SO42-, H+, NH4+, NO3-, NH3, and nitrous acid in homes with and without kerosene space heaters	Environmental Health Perspectives	107	3	223-231
Li WW, H Paschold, H Morales, J Chianelli	2003	Correlations between short-term indoor and outdoor PM concentrations at residences with evaporative coolers	Atmospheric Environment	37	19	2691-2703
Linn WS, H Gong, KW Clark, KR Anderson	1999	Day-to-day particulate exposures and health changes in Los Angeles area residents with severe lung disease	Journal of the Air & Waste Management Association	49		108-115
Lioy PJ, NC Freeman, T Wainman, AH Stern, R Boesch, T Howell, SI Shupack	1992	Microenvironmental analysis of residential exposure to chromium-laden wastes in and around New Jersey homes	Risk Anal	12	2	287-99
Lioy, P. J., Waldman, J. M., Buckley, T., Butler, J., & Pietarinen, C.	1990	The personal, indoor and outdoor concentrations of PM-10 measured in an industrial community during the winter	Atmospheric Environment. Part B. Urban Atmosphere	24	1	57-66

Liu LJ, M Box, D Kalman, J Kaufman, J Koenig, T Larson, T Lumley, L	2003	Exposure assessment of particulate matter for susceptible populations in Seattle	Environ Health Perspect	111	7	909-18
Long CM, HH Suh, P Koutrakis	2000	Characterization of indoor particle sources using continuous mass and size monitors	Journal of the Air & Waste Management Association	50	7	1236-1250
McCormack MC, PN Breysse, NN Hansel, EC Matsui, ES Tonorezos, J	2008	Common household activities are associated with elevated particulate matter concentrations in bedrooms of inner-city Baltimore pre-school children	Environmental Research	106	2	148-155
Patton AP, L Calderon, YY Xiong, ZC Wang, J Senick, MS Allacci, D Plotnik, R	2016	Airborne Particulate Matter in Two Multi-Family Green Buildings: Concentrations and Effect of Ventilation and Occupant Behavior	International Journal of Environmental Research and Public Health	13	1	
Pavilonis BT, TR Anthony, PT O'Shaughnessy, MJ Humann, JA Merchant, G	2013	Indoor and outdoor particulate matter and endotoxin concentrations in an intensely agricultural county	Journal of Exposure Science and Environmental Epidemiology	23	3	299-305
Pellizzari ED, CA Clayton, CE Rodes, RE Mason, LL Piper, B Fort, G Pfeifer, D Lynam	2001	Particulate matter and manganese exposures in Indianapolis, Indiana	Journal of Exposure Analysis and Environmental Epidemiology	11	6	423-440
Robin LF, PS Less, M Winget, M Steinhoff, LH Moulton, M Santosham, A	1996	Wood-burning stoves and lower respiratory illnesses in Navajo children	Pediatr Infect Dis J	15	10	859-65
Rojas-Bracho L, HH Suh, P Koutrakis	2000	Relationships among personal, indoor, and outdoor fine and coarse particle concentrations for individuals with COPD	Journal of Exposure Analysis and Environmental Epidemiology	10	3	294-306
Simons E, J Curtin-Brosnan, T Buckley, P Breysse, PA Eggleston	2007	Indoor environmental differences between inner city and suburban homes of children with asthma	Journal of Urban Health-Bulletin of the New York Academy of Medicine	84	4	577-590

Williams R, J Creason, R Zweidinger, R Watts, L Sheldon, C Shy	2000	Indoor, outdoor, and personal exposure monitoring of particulate air pollution: the Baltimore elderly epidemiology-exposure pilot study	Atmospheric Environment	34	24	4193-4204
Williams R, J Suggs, A Rea, K Leovic, A Vette, C Croghan, L Sheldon, C	2003	The Research Triangle Park particulate matter panel study: PM mass concentration relationships	Atmospheric Environment	37	38	5349-5363
Williams R, J Suggs, R Zweidinger, G Evans, J Creason, R Kwok, C Rodes,	2000	The 1998 Baltimore particulate matter Epidemiology-Exposure Study: Part 1. Comparison of ambient, residential outdoor, indoor and apartment particulate matter monitoring	Journal of Exposure Analysis and Environmental Epidemiology	10	6	518-532
Xiong Y, U Krogmann, G Mainelis, LA Rodenburg, CJ Andrews	2015	Indoor air quality in green buildings: A case-study in a residential high-rise building in the northeastern United States	J Environ Sci Health A Tox Hazard Subst Environ Eng	50	3	225-42
Xu Y, S Raja, AR Ferro, PA Jaques, PK Hopke, C Gressani, LE Wetzel	2010	Effectiveness of heating, ventilation and air conditioning system with HEPA filter unit on indoor air quality and asthmatic children's health	Building and Environment	45	2	330-337
Yip FY, GJ Keeler, JT Dvonch, TG Robins, EA Parker, BA Israel, W	2004	Personal exposures to particulate matter among children with asthma in Detroit, Michigan	Atmospheric Environment	38	31	5227-5236

Oceania PM10

Australia PM10

He C, H Salonen, X Ling, L Crilley, N Jayasundara, HC Cheung, M Hargreaves, F	2014	The impact of flood and post-flood cleaning on airborne microbiological and particle contamination in residential houses	Environment International	69		43725
Jones J, S Stick, P Dingle, P Franklin	2007	Spatial variability of particulates in homes: Implications for infant exposure	Science of the Total Environment	376		317-323

Molloy SB, M Cheng, IE Galbally, MD Keywood, SJ Lawson, JC Powell, R	2012	Indoor air quality in typical temperate zone Australian dwellings	Atmospheric Environment	54		400-407

South America PM10

Brazil PM10

Segalin B, P Kumar, K Micadei, A Fornaro, FLT Goncalves	2017	Size-segregated particulate matter inside residences of elderly in the Metropolitan Area of Sao Paulo, Brazil	Atmospheric Environment	148		139-151

Chile PM10

Adonis M, L Gil	2001	Indoor air pollution in a zone of extreme poverty of metropolitan Santiago, Chile	Indoor and Built Environment	10		138-146
Rojas-Bracho L, HH Suh, P Oyola, P Koutrakis	2002	Measurements of children's exposures to particles and nitrogen dioxide in Santiago, Chile	Science of the Total Environment	287	3	249-264

Ecuador PM10

Raysoni AU, RX Armijos, MM Weigel, T Montoya, P Eschanique, M Racines,	2016	Assessment of indoor and outdoor PM species at schools and residences in a high-altitude Ecuadorian urban center	Environmental Pollution	214		668-679

South Asia PM10

Bangladesh PM10

Akther T, Ahmed M, Shohel M, Ferdousi FK, Salam A.	2019	Particulate matters and gaseous pollutants in indoor environment and Association of ultra-fine particulate matters (PM1) with lung function.		26	6	5475-84.

	India	PM10				
Gupta S, A Srivastava, VK Jain	2008	Particle size distribution of aerosols and associated heavy metals in kitchen environments	Environ Monit Assess	142		141-8
Khillare PS, R Pandey, S Balachandran	2004	Characterisation of indoor PM10 in residential areas of Delhi	Indoor and Built Environment	13	2	139-147
Kulshreshtha P, M Khare, P Seetharaman	2008	Indoor air quality assessment in and around urban slums of Delhi city, India	Indoor Air	18	6	488-98
Kulshrestha A, DD Massey, J Masih, A Taneja	2014	Source Characterization of Trace Elements in Indoor Environments at Urban, Rural and Roadside Sites in a Semi Arid Region of India	Aerosol and Air Quality Research	14	6	1738-1751
Kumar P	2001	Characterisation of indoor respirable dust in a locality of Delhi, India	Indoor and Built Environment	10	2	95-102
Lawrence A, N Fatima	2014	Urban air pollution & its assessment in Lucknow City - The second largest city of North India	Science of the Total Environment	488		449-457
Massey D, A Kulshrestha, J Masih, A Taneja	2012	Seasonal trends of PM10, PM5.0, PM2.5 & PM1.0 in indoor and outdoor environments of residential homes located in North-Central India	Building and Environment	47		223-231
Parikh J, K Balakrishnan, V Laxmi, H Biswas	2001	Exposure from cooking with biofuels: pollution monitoring and analysis for rural Tamil Nadu, India	Energy	26	10	949-962
Priyamvada H, C Priyanka, RK Singh, M Akila, R Ravikrishna, SS Gunthe	2018	Assessment of PM and bioaerosols at diverse indoor environments in a southern tropical Indian region	Building and Environment	137		215-225

Roy R, R Jan, S Yadav, MH Vasave, PG Satsangi	2016	Study of metals in radical-mediated toxicity of particulate matter in indoor environments of Pune, India	Air Quality Atmosphere and Health	9	6	669-680
Satsangi PG, S Yadav, AS Pipal, N Kumbhar	2014	Characteristics of trace metals in fine (PM2.5) and inhalable (PM10) particles and its health risk assessment along with in-silico approach in indoor environment of India	Atmospheric Environment	92		384-393
Srivastava A, S Gupta, VK Jain	2009	Winter-time size distribution and source apportionment of total suspended particulate matter and associated metals in Delhi	Atmospheric Research	92	1	88-99
	Pakistan	PM10				
Ali Z, F Naz, ZA Nasir, S Sidra, I Colbeck	2015	PARTICULATE POLLUTION IN URBAN RESIDENTIAL BUILT ENVIRONMENTS DURING WINTER AND SUMMER SEASONS IN LAHORE, PAKISTAN	Journal of Animal and Plant Sciences	25	3	660-665
Nasir, Z. A., Colbeck, I., Ali, Z., & Ahmad, S	2013	Indoor particulate matter in developing countries: a case study in Pakistan and potential intervention strategies	Environmental Research Letters	8	2	024002
	Sri Lanka	PM10				
Ranasinghe RSA, AGT Sugathapala, SC Lee, WT Hung, KF Ho, CS Chan, Y	2015	Exploratory study of the indoor and outdoor relationships and chemical compositions of particulate matter in urban households in Colombo	Indoor and Built Environment	24	5	597-606

South East Asia PM10

Malaysia PM10

Nazariah, S.S.N., Juliana, J. and Abdah, M.A.	2013	Interleukin-6 via sputum induction as biomarker of inflammation for indoor particulate matter among primary school children in Klang Valley, Malaysia	Global journal of health science	5	4	93

Thailand PM10

Kammoolkon R, Taneepanichskul N, Siriwong W, Pitaknoppakul	2018	The relationship between household particulate matter and an increase of carotid intima-media thickness [cimt]: A one-year follow-up study.	Journal of the Medical Association of Thailand	101	11	1529-36
Lappharat S, Taneepanichskul N, Reutrakul S, Chirakalwasan	2018	Effects of bedroom environmental conditions on the severity of obstructive sleep apnea.	Journal of clinical sleep medicine: JCSM: Official Publication of the American Academy of Sleep Medicine	14	4	565-73
Srithawirat T, MT Latif, FR Sulaiman	2016	Indoor PM10 and its heavy metal composition at a roadside residential environment, Phitsanulok, Thailand	Atmosfera	29	4	311-322
Tsai FC, KR Smith, N Vichit-Vadakan, BD Ostro, LG Chestnut, N Kungskulniti	2000	Indoor/outdoor PM10 and PM2.5 in Bangkok, Thailand	Journal of Exposure Analysis and Environmental Epidemiology	10	1	15-26

West Asia & North Africa PM10

Egypt PM10

Abdel-Salam MM	2013	Indoor particulate matter in urban residences of Alexandria, Egypt	J Air Waste Manag Assoc	63	8	956-62

Wagdi D, K Tarabieh, MN Abou Zeid	2018	Indoor air quality index for preoccupancy assessment	Air Quality Atmosphere and Health	11	4	445-458
	Iran	PM10				
Hassanvand MS, K Naddafi, H Kashani, S Faridi, N Kunzli, R Nabizadeh, F	2017	Short-term effects of particle size fractions on circulating biomarkers of inflammation in a panel of elderly subjects and healthy young adults	Environmental Pollution	223		695-704
Hassanvand MS, K Naddafi, S Faridi, M Arhami, R Nabizadeh, MH Sowlat, Z	2014	Indoor/outdoor relationships of PM10, PM2.5, and PM1 mass concentrations and their water-soluble ions in a retirement home and a school dormitory	Atmospheric Environment	82		375-382
Mirhoseini SH, M Nikaeen, K Satoh, K Makimura	2016	Assessment of Airborne Particles in Indoor Environments: Applicability of Particle Counting for Prediction of Bioaerosol Concentrations	Aerosol and Air Quality Research	16	8	1903-1910
	Israel	PM10				
Jodeh S, AR Hasan, J Amarah, F Judeh, R Salghi, H Lgaz, W Jodeh	2018	Indoor and outdoor air quality analysis for the city of Nablus in Palestine: seasonal trends of PM10, PM5.0, PM2.5, and PM1.0 of residential homes	Air Quality Atmosphere and Health	11	2	229-237
Krasnov H, I Katra, MD Friger	2015	Insights into Indoor/Outdoor PM Concentration Ratios due to Dust Storms in an Arid Region	Atmosphere	6	7	879-890
Krasnov H, I Katra, V Novack, A Vodonos, MD Friger	2015	Increased indoor PM concentrations controlled by atmospheric dust events and urban factors	Building and Environment	87		169-176

Lebanon PM10

Saliba NA, M Atallah, G Al-Kadamany	2009	Levels and indoor-outdoor relationships of PM10 and soluble inorganic ions in Beirut, Lebanon	Atmospheric Research	92	1	131-137

Saudi Arabia PM10

Ali N.	2019	Polycyclic aromatic hydrocarbons (PAHs) in indoor air and dust samples of different Saudi microenvironments; health and carcinogenic risk assessment for the general population.		696		133995

Turkey PM10

Pekey B, ZB Bozkurt, H Pekey, G Dogan, A Zararsiz, N Efe, G Tuncel	2010	Indoor/outdoor concentrations and elemental composition of PM10/PM2.5 in urban/industrial areas of Kocaeli City, Turkey	Indoor Air	20	2	112-125

United Arab Emirates PM10

Yeatts KB, M El-Sadig, D Leith, W Kalsbeek, F Al-Maskari, D Couper, WE	2012	Indoor Air Pollutants and Health in the United Arab Emirates	Environmental Health Perspectives	120	5	687-694

Western Europe PM10

Belgium PM10

Bentayeb M, D Norback, M Bednarek, A Bernard, GH Cai, S Cerrai, KK	2015	Indoor air quality, ventilation and respiratory health in elderly residents Living in nursing homes in Europe	European Respiratory Journal	45	5	1228-1238
Stranger M, SS Potgieter-Vermaak, R Van Grieken	2009	Particulate matter and gaseous pollutants in residences in Antwerp, Belgium	Science of the Total Environment	407	3	1182-1192

	Denmark	PM10				
Bentayeb M, D Norback, M Bednarek, A Bernard, GH Cai, S Cerrai, KK	2015	Indoor air quality, ventilation and respiratory health in elderly residents Living in nursing homes in Europe	European Respiratory Journal	45	5	1228-1238
Karottki DG, G Beko, G Clausen, AM Madsen, ZJ Andersen, A Massling, M	2014	Cardiovascular and lung function in relation to outdoor and indoor exposure to fine and ultrafine particulate matter in middle-aged subjects	Environment International	73		372-381
Karottki DG, M Spilak, M Frederiksen, ZJ Andersen, AM Madsen, M Ketzel, A	2015	Indoor and Outdoor Exposure to Ultrafine, Fine and Microbiologically Derived Particulate Matter Related to Cardiovascular and Respiratory Effects in a Panel of Elderly Urban Citizens	International Journal of Environmental Research and Public Health	12	2	1667-1686
	Finland	PM10				
de Hartog JJ, JG Ayres, A Karakatsani, A Analitis, H Brink, K Hameri, RM	2010	Lung function and indicators of exposure to indoor and outdoor particulate matter among asthma and COPD patients	Occupational and Environmental Medicine	67	1	43506
Du L, T Prasauskas, V Leivo, M Turunen, M Pekkonen, M Kiviste, A Aaltonen, D	2015	Assessment of indoor environmental quality in existing multi-family buildings in North-East Europe	Environ Int	79		74-84
Hoek G, G Kos, RM Harrison, J de Hartog, K Meliefste, H ten Brink, K	2008	Indoor-outdoor relationships of particle number and mass in four European cities	Atmospheric Environment	42	1	156-169
Sippula O, H Rintala, M Happo, P Jalava, K Kuuspalo, A Viren, A	2013	Characterization of Chemical and Microbial Species from Size-Segregated Indoor and Outdoor Particulate Samples	Aerosol and Air Quality Research	13	4	1212-U343

France PM10

Bentayeb M, D Norback, M Bednarek, A Bernard, GH Cai, S Cerrai, KK	2015	Indoor air quality, ventilation and respiratory health in elderly residents Living in nursing homes in Europe	European Respiratory Journal	45	5	1228-1238
Gauvin S, P Reungoat, S Cassadou, J Dechenaux, I Momas, J Just, D Zmirou	2002	Contribution of indoor and outdoor environments to PM2.5 personal exposure of children - VESTA study	Science of the Total Environment	297		175-181
Langer S, O Ramalho, M Derbez, J Riberon, S Kirchner, C Mandin	2016	Indoor environmental quality in French dwellings and building characteristics	Atmospheric Environment	128		82-91
Maesano CN, Caillaud D, Youssouf H, Banerjee S, Prud'Homme J, Audi C, et	2019	Indoor exposure to particulate matter and volatile organic compounds in dwellings and workplaces and respiratory health in French farmers.	Multidisciplinary Respiratory Medicine	14	1	eCollection
Nerriere E, D Zmirou-Navier, O Blanchard, I Momas, J Ladner, Y Le	2005	Can we use fixed ambient air monitors to estimate population long-term exposure to air pollutants? The case of spatial variability in the Genotox ER study	Environmental Research	97	1	32-42
Ramalho O, G Wyart, C Mandin, P Blondeau, PA Cabanes, N Leclerc, JU	2015	Association of carbon dioxide with indoor air pollutants and exceedance of health guideline values	Building and Environment	93		115-124

Germany PM10

Franck U, O Herbarth, S Roder, U Schlink, M Borte, U Diez, U Kramer, I Lehmann	2011	Respiratory effects of indoor particles in young children are size dependent	Science of the Total Environment	409	9	1621-1631
Oeder S, S Dietrich, I Weichenmeier, W Schober, G Pusch, RA Jorres, R	2012	Toxicity and elemental composition of particulate matter from outdoor and indoor air of elementary schools in Munich, Germany	Indoor Air	22	2	148-158

Santen M, M Wesselmann, U Fittschen, R Cremer, P Braun, A Ludecke, HJ	2009	Measurements of fine and ultrafine particles in indoor environment of living rooms	Gefahrstoffe Reinhaltung Der Luft	69	3	63-70
	Greece	PM10				
Bentayeb M, D Norback, M Bednarek, A Bernard, GH Cai, S Cerrai, KK	2015	Indoor air quality, ventilation and respiratory health in elderly residents Living in nursing homes in Europe	European Respiratory Journal	45	5	1228-1238
de Hartog JJ, JG Ayres, A Karakatsani, A Analitis, H Brink, K Hameri, RM	2010	Lung function and indicators of exposure to indoor and outdoor particulate matter among asthma and COPD patients	Occupational and Environmental Medicine	67	1	43506
Diapouli E, A Chaloulakou, N Spyrellis	2008	Indoor and outdoor PM concentrations at a residential environment, in the Athens area	Global Nest Journal	10	2	201-208
Diapouli E, A Chaloulakou, N Spyrellis	2010	INDOOR/OUTDOOR PM LEVELS AND EC SURROGATE, AT TYPICAL MICROENVIRONMENTS IN THE ATHENS AREA	Global Nest Journal	12	1	43818
Diapouli E, K Eleftheriadis, AA Karanasiou, S Vratolis, O Hermansen, I Colbeck, M	2011	Indoor and Outdoor Particle Number and Mass Concentrations in Athens. Sources, Sinks and Variability of Aerosol Parameters	Aerosol and Air Quality Research	11	6	632-642
Hoek G, G Kos, RM Harrison, J de Hartog, K Meliefste, H ten Brink, K	2008	Indoor-outdoor relationships of particle number and mass in four European cities	Atmospheric Environment	42	1	156-169
Stamatelopoulou A, Asimakopoulos DN, Maggos T.	2019	Effects of PM, TVOCs and comfort parameters on indoor air quality of residences with young children.	Building and Environment	150		233-44

	Ireland	PM10				
Guo L, JO Lewis, JP McLaughlin	2008	Emissions from Irish domestic fireplaces and their impact on indoor air quality when used as supplementary heating source	Global Nest Journal	10	2	209-216
	Italy	PM10				
Bentayeb M, D Norback, M Bednarek, A Bernard, GH Cai, S Cerrai, KK	2015	Indoor air quality, ventilation and respiratory health in elderly residents Living in nursing homes in Europe	European Respiratory Journal	45	5	1228-1238
Cattaneo A, C Peruzzo, G Garramone, P Urso, R Ruggeri, P Carrer, DM	2011	Airborne particulate matter and gaseous air pollutants in residential structures in Lodi province, Italy	Indoor Air	21	6	489-500
Frasca D, Marcoccia M, Tofful L, Simonetti G, Perrino C, Canepari S.	2018	Influence of advanced wood-fired appliances for residential heating on indoor air quality.	Chemosphere	211		62-71
Manigrasso M, Protano C, Astolfi ML, Massimi L, Avinod P, Vitali M, et al.	2019	Evidences of copper nanoparticle exposure in indoor environments: Long-term assessment, high-resolution field emission scanning electron microscopy evaluation, in silico respiratory dosimetry study and possible health implications.	Science of the Total Environment	653		Epub
Scapellato ML, C Canova, A de Simone, M Carrieri, P Maestrelli, L Simonato, GB	2009	Personal PM10 exposure in asthmatic adults in Padova, Italy: seasonal variability and factors affecting individual concentrations of particulate matter	International Journal of Hygiene and Environmental Health	212	6	626-636
	Netherlands	PM10				
de Hartog JJ, JG Ayres, A Karakatsani, A Analitis, H Brink, K Hameri, RM	2010	Lung function and indicators of exposure to indoor and outdoor particulate matter among asthma and COPD patients	Occupational and Environmental Medicine	67	1	43506

Fischer PH, G Hoek, H van Reeuwijk, DJ Briggs, E Lebret, JH van Wijnen, S	2000	Traffic-related differences in outdoor and indoor concentrations of particles and volatile organic compounds in Amsterdam	Atmospheric Environment	34	22	3713-3722
Hoek G, G Kos, RM Harrison, J de Hartog, K Meliefste, H ten Brink, K	2008	Indoor-outdoor relationships of particle number and mass in four European cities	Atmospheric Environment	42	1	156-169
	Norway	PM10				
Lazaridis M, V Aleksandropoulou, JE Hanssen, C Dye, K	2008	Inorganic and carbonaceous components in indoor/outdoor particulate matter in two residential houses in Oslo, Norway	Journal of the Air & Waste Management Association	58	3	346-356
Oie L, P Magnus, BV Johansen	1997	Suspended particulate matter in Norwegian dwellings in relation to fleece and shelf factors, domestic smoking, air exchange rate, and presence of hot wire convection heaters	Environment International	23	4	465-473
Ormstad H, PI Gaarder, BV Johansen	1997	Quantification and characterisation of suspended particulate matter in indoor air	Science of the Total Environment	193	3	185-196
Rakkestad KE, CJ Dye, KE Yttri, JA Holme, JK Hongslo, PE Schwarze, R Becher	2007	Phthalate levels in Norwegian indoor air related to particle size fraction	Journal of Environmental Monitoring	9	12	1419-1425
	Portugal	PM10				
Almeida-Silva M, Pilou M, Housiadas C, Almeida SM.	2018	Internal dose of particles in the elderly—modeling based on aerosol measurements.	Environmental Science and Pollution Research	25	24	23645-56

Almeida-Silva M, SM Almeida, JF Gomes, PC Albuquerque, HT	2014	Determination of airborne nanoparticles in elderly care centers	J Toxicol Environ Health A	77		867-78
Almeida-Silva M, T Faria, D Saraga, T Maggos, HT Wolterbeek, SM Almeida	2016	Source apportionment of indoor PM10 in Elderly Care Centre	Environmental Science and Pollution Research	23	8	7814-7827
Almeida-Silva, M., Wolterbeek, H.T. and Almeida, S.M	2014	Elderly exposure to indoor air pollutants	Atmospheric Environment	85		54-63
Belo J, Carreiro-Martins P, Papoila AL, Palmeiro T, Caires I, Alves M, et al.	2019	The impact of indoor air quality on respiratory health of older people living in nursing homes: spirometric and exhaled breath condensate assessments.	Journal of Environmental Science and Health Part a-Toxic/Hazardous Substances & Environmental Engineering	54	12	1153-8
Cerqueira M, D Marques, A Caseiro, C Pio	2010	Experimental evidence for a significant contribution of cellulose to indoor aerosol mass concentration	Atmospheric Environment	44	6	867-871
Custódio, D., Pinho, I., Cerqueira, M., Nunes, T., & Pio, C.	2014	Indoor and outdoor suspended particulate matter and associated carbonaceous species at residential homes in northwestern Portugal	Science of the total environment	473		72-76
Madureira J, I Paciencia, J Cavaleiro-Rufo, E de Oliveira Fernandes	2016	Indoor pollutant exposure among children with and without asthma in Porto, Portugal, during the cold season	Environ Sci Pollut Res Int	23	20	20539-
Mendes A, AL Papoila, P Carreiro-Martins, S Bonassi, I Caires, T	2016	The impact of indoor air quality and contaminants on respiratory health of older people living in long-term care residences in Porto	Age and Ageing	45	1	136-142

Slezakova K, D Castro, C Delerue-Matos, S Morais, MD Pereira	2014	Levels and risks of particulate-bound PAHs in indoor air influenced by tobacco smoke: a field measurement	Environmental Science and Pollution Research	21	6	4492-4501
	Sweden	PM10				
Bentayeb M, D Norback, M Bednarek, A Bernard, GH Cai, S Cerrai, KK	2015	Indoor air quality, ventilation and respiratory health in elderly residents Living in nursing homes in Europe	European Respiratory Journal	45	5	1228-1238
	Switzerland	PM10				
Meier R, M Eeftens, HC Phuleria, A Ineichen, E Corradi, M Davey, M Fierz,	2015	Differences in indoor versus outdoor concentrations of ultrafine particles, PM2.5, PMabsorbance and NO2 in Swiss homes	J Expo Sci Environ Epidemiol	25	5	499-505
Monn C, A Fuchs, D Hogger, M Junker, D Kogelschatz, N Roth, HU Wanner	1997	Particulate matter less than 10 mu m (PM10) and fine particles less than 2.5 mu m (PM2.5): relationships between indoor, outdoor and personal concentrations	Science of the Total Environment	208		15-21
	UK	PM10				
Aizlewood C, C Dimitroulopoulou	2006	The HOPE project: The UK experience	Indoor and Built Environment	15	5	393-409
BeruBe KA, KJ Sexton, TP Jones, T Moreno, S Anderson, RJ Richards	2004	The spatial and temporal variations in PM10 mass from six UK homes	Science of the Total Environment	324		41-53
de Hartog JJ, JG Ayres, A Karakatsani, A Analitis, H Brink, K Hameri, RM	2010	Lung function and indicators of exposure to indoor and outdoor particulate matter among asthma and COPD patients	Occupational and Environmental Medicine	67	1	43506

Hoek G, G Kos, RM Harrison, J de Hartog, K Meliefste, H ten Brink, K	2008	Indoor-outdoor relationships of particle number and mass in four European cities	Atmospheric Environment	42	1	156-169
Jones NC, CA Thornton, D Mark, RM Harrison	2000	Indoor/outdoor relationships of particulate matter in domestic homes with roadside, urban and rural locations	Atmospheric Environment	34	16	2603-2612
Kingham S, D Briggs, P Elliott, P Fischer, E Lebret	2000	Spatial variations in the concentrations of traffic-related pollutants in indoor and outdoor air in Huddersfield, England	Atmospheric Environment	34	6	905-916
Mohammadyan M, M Ashmore	2005	Personal exposure and indoor PM2.5 concentrations in an urban population	Indoor and Built Environment	14		313-320
Nasir ZA, I Colbeck	2013	Particulate pollution in different housing types in a UK suburban location	Science of the Total Environment	445		165-176
Wigzell E, M Kendall, MJ Nieuwenhuijsen	2000	The spatial and temporal variation of particulate matter within the home	Journal of Exposure Analysis and Environmental Epidemiology	10	3	307-314

PM100

North America PM100

USA PM100

Shalat SL, PJ Lioy, K Schmeelck, G Mainelis	2007	Improving estimation of indoor exposure to inhalable particles for children in the first year of life	Journal of the Air & Waste Management Association	57	8	934-939

PM1-10

East Asia PM1-10

Taiwan PM1-10

Lin LY, IJ Liu, HC Chuang, HY Lin, KJ Chuang	2013	Size and composition effects of household particles on inflammation and endothelial dysfunction of human coronary artery endothelial cells	Atmospheric Environment	77		490-495

North America PM1-10

USA PM1-10

Thatcher TL, DW Layton	1995	DEPOSITION, RESUSPENSION, AND PENETRATION OF PARTICLES WITHIN A RESIDENCE	Atmospheric Environment	29	13	1487-1497

Western Europe PM1-10

Sweden PM1-10

Isaxon C, A Gudmundsson, EZ Nordin, L Lonnblad, A Dahl, G Wieslander, M	2015	Contribution of indoor-generated particles to residential exposure	Atmospheric Environment	106		458-466

PM1-5

North America PM1-5

USA PM1-5

Du L, S Batterman, E Parker, C Godwin, JY Chin, A O'Toole, T Robins, W	2011	Particle concentrations and effectiveness of free-standing air filters in bedrooms of children with asthma in Detroit, Michigan	Building and Environment	46	11	2303-2313

PM2

East Asia PM2

Japan PM2

Funasaka K, T Miyazaki, K Tsuruho, K Tamura, T Mizuno, K Kuroda	2000	Relationship between indoor and outdoor carbonaceous particulates in roadside households	Environmental Pollution	110	1	127-134

Western Europe PM2

Greece PM2

Diapouli E, K Eleftheriadis, AA Karanasiou, S Vratolis, O Hermansen, I Colbeck, M	2011	Indoor and Outdoor Particle Number and Mass Concentrations in Athens. Sources, Sinks and Variability of Aerosol Parameters	Aerosol and Air Quality Research	11	6	632-642

PM2.5

Africa (except North) PM2.5

Ethiopia PM2.5

Embiale A, Zewge F, Chandravanshi BS, Sahle-Demessie E.	2019	Short-term exposure assessment to particulate matter and total volatile organic compounds in indoor air during cooking Ethiopian sauces (Wot) using electricity, kerosene and charcoal fuels.	Indoor and Built Environment	28	8	1140-54

Nigeria PM2.5

Ibhafidon LI, DO Obaseki, GE Erhabor, AA Akor, I Irabor, I Obioh	2014	Respiratory symptoms, lung function and particulate matter pollution in residential indoor environment in Ile-Ife, Nigeria	Niger Med J	55	1	48-53

	South Africa	PM2.5				
Gumede PR, MJ Savage	2017	Respiratory health effects associated with indoor particulate matter (PM2.5) in children residing near a landfill site in Durban, South Africa	Air Quality Atmosphere and Health	10	7	853-860

Central America and Caribbean PM2.5

	Mexico	PM2.5				
Cortez-Lugo M, H Moreno-Macias, F Holguin-Molina, JC Chow, JG Watson, V	2008	Relationship between indoor, outdoor, and personal fine particle concentrations for individuals with COPD and predictors of indoor-outdoor ratio in Mexico city	Journal of Exposure Science and Environmental Epidemiology	18	1	109-115
Cortez-Lugo M, S Rodriguez-Dozal, I Rosas-Perez, U Alamo-Hernandez,	2015	Modeling and estimating manganese concentrations in rural households in the mining district of Molango, Mexico	Environmental Monitoring and Assessment	187	12	
Holguin F, MM Tellez-Rojo, M Hernandez, M Cortez, JC Chow, JG Watsow, D	2003	Air pollution and heart rate variability among the elderly in Mexico City	Epidemiology	14	5	521-527
Vallejo M, C Lerma, O Infante, AG Hermosillo, H Riojas-Rodriguez, M	2004	Personal exposure to particulate matter less than 2.5 mu m in Mexico City: a pilot study	Journal of Exposure Analysis and Environmental Epidemiology	14	4	323-329

East Asia PM2.5

	China	PM2.5				
Ai ZT, CM Mak, DJ Cui	2015	On-site measurements of ventilation performance and indoor air quality in naturally ventilated high-rise residential buildings in Hong Kong	Indoor and Built Environment	24	2	214-224

Brehmer C, Norris C, Barkjohn KK, Bergin MH, Zhang J, Cui X, et al.	2019	The impact of household air cleaners on the oxidative potential of PM2.5 and the role of metals and sources associated with indoor and outdoor exposure.	Environmental Research			108919
Cai J, Yu W, Li BZ, Yao RM, Zhang TJW, Guo M, et al.	2019	Particle removal efficiency of a household portable air cleaner in real-world residences: A single-blind cross-over field study.	Energy and Buildings	203		
Cao G, Bi J, Ma Z, Shao Z, Wang J.	2019	Seasonal Characteristics of the Chemical Composition of Fine Particles in Residences of Nanjing, China.	International Journal of Environmental Research and Public Health	16	6	E1066
Cao JJ, H Huang, SC Lee, JC Chow, CW Zou, KF Ho, JG Watson	2012	Indoor/Outdoor Relationships for Organic and Elemental Carbon in PM2.5 at Residential Homes in Guangzhou, China	Aerosol and Air Quality Research	12	5	902-910
Cao JJ, SC Lee, JC Chow, Y Cheng, KF Ho, K Fung, SX Liu, JG Watson	2005	Indoor/outdoor relationships for PM2.5 and associated carbonaceous pollutants at residential homes in Hong Kong - case study	Indoor Air	15	3	197-204
Chao CY, EC Cheng	2002	Source apportionment of indoor PM(2.5) and PM(10) in homes	Indoor and Built Environment	11	1	27-37
Chao CY, KK Wong	2002	Residential indoor PM10 and PM2.5 in Hong kong and the elemental composition	Atmospheric Environment	36	2	265-277
Chao CY, KK Wong, EC Cheng	2002	Size distribution of indoor particulate matter in 60 homes in Hong Kong	Indoor and Built Environment	11	1	18-26

Chao CYH, TC Tung	2001	An empirical model for outdoor contaminant transmission into residential buildings and experimental verification	Atmospheric Environment	35	9	1585-1596
Chen Y, D Lv, XH Li, TL Zhu	2018	PM2.5-bound phthalates in indoor and outdoor air in Beijing: Seasonal distributions and human exposure via inhalation	Environmental Pollution	241		369-377
Chen Y, Zhang HB, Yoshino H, Xie JC, Yanagi U, Hasegawa K, et al.	2019	Winter indoor environment of elderly households: A case of rural regions in northeast and southeast China.	Building and Environment	165		Epub
Chen, Y., Li, X., Zhu, T., Han, Y. and Lv, D.,	2017	PM2. 5-bound PAHs in three indoor and one outdoor air in Beijing: Concentration, source and health risk assessment	Science of The Total Environment	586		255-264
Cheung PK, Jim CY.	2019	Indoor air quality in substandard housing in Hong Kong.	Sustainable Cities and Society	48		101583
Chu YY, P Xu, ZW Yang, WL Li	2018	Retrofitting existing buildings to control indoor PM2.5 concentration on smog days: Initial experience of residential buildings in China	Building Services Engineering Research & Technology	39	3	263-283
Cui X, F Li, J Xiang, L Fang, MK Chung, DB Day, J Mo, CJ Weschler, J Gong, L He, D	2018	Cardiopulmonary effects of overnight indoor air filtration in healthy non-smoking adults: A double-blind randomized crossover study	Environ Int	114		27-36
Dai XL, JJ Liu, XD Li, L Zhao	2018	Long-term monitoring of indoor CO2 and PM2.5 in Chinese homes: Concentrations and their relationships with outdoor environments	Building and Environment	144		238-247

Day DB, J Xiang, J Mo, MA Clyde, CJ Weschler, F Li, J Gong, M Chung, Y Zhang, J	2018	Combined use of an electrostatic precipitator and a high-efficiency particulate air filter in building ventilation systems: Effects on cardiorespiratory health indicators in healthy adults	Indoor Air	177	9	1344-1353
Deng GF, ZH Li, ZC Wang, JB Gao, ZW Xu, JD Li, ZY Wang	2017	Indoor/outdoor relationship of PM2.5 concentration in typical buildings with and without air cleaning in Beijing	Indoor and Built Environment	26	1	60-68
Ding A, YJ Yang, ZH Zhao, A Huls, A Vierkotter, ZY Yuan, J Cai, J Zhang, WS Gao, JX	2017	Indoor PM2.5 exposure affects skin aging manifestation in a Chinese population	Scientific Reports	7		
Fan GT, JC Xie, HS Yoshino, U Yanagi, K Hasegawa, N Kagi, T Goto, QY Zhang, CY	2018	Indoor environmental conditions in urban and rural homes with older people during heating season: A case in cold region, China	Energy and Buildings	167		334-346
Fan GT, JC Xie, JP Liu, H Yoshino	2017	Investigation of indoor environmental quality in urban dwellings with schoolchildren in Beijing, China	Indoor and Built Environment	26	5	694-716
Han Y, M Qi, Y Chen, H Shen, J Liu, Y Huang, H Chen, W Liu, X Wang, J Liu,	2015	Influences of ambient air PM(2).(5) concentration and meteorological condition on the indoor PM(2).(5) concentrations in a residential apartment in Beijing using a new approach	Environ Pollut	205		307-14
Han YJ, XH Li, TL Zhu, D Lv, Y Chen, LA Hou, YP Zhang, MZ Ren	2016	Characteristics and Relationships between Indoor and Outdoor PM2.5 in Beijing: A Residential Apartment Case Study	Aerosol and Air Quality Research	16	10	2386-2395
Ho KF, JJ Cao, RM Harrison, SC Lee, KK Bau	2004	Indoor/outdoor relationships of organic carbon (OC) and elemental carbon (EC) in PM2.5 in roadside environment of Hong Kong	Atmospheric Environment	38	37	6327-6335

Hu J, N Li, Y Lv, J Liu, J Xie, H Zhang	2017	Investigation on Indoor Air Pollution and Childhood Allergies in Households in Six Chinese Cities by Subjective Survey and Field Measurements	Int J Environ Res Public Health	14	9	
Hu JH, NP Li, H Yoshino, U Yanagi, K Hasegawa, N Kagi, YD He, XQ Wei	2017	Field study on indoor health risk factors in households with schoolchildren in south-central China	Building and Environment	117		260-273
Huang C, X Wang, W Liu, J Cai, L Shen, Z Zou, R Lu, J Chang, X Wei, C Sun, Z	2016	Household indoor air quality and its associations with childhood asthma in Shanghai, China: On-site inspected methods and preliminary results	Environ Res	151		154-167
Huang KL, JS Song, GH Feng, QP Chang, B Jiang, J Wang, W Sun, HX Li, JM	2018	Indoor air quality analysis of residential buildings in northeast China based on field measurements and longtime monitoring	Building and Environment	144		171-183
Huang L, Z Pu, M Li, J Sundell	2015	Characterizing the Indoor-Outdoor Relationship of Fine Particulate Matter in Non-Heating Season for Urban Residences in Beijing	PLoS One	10	9	e0138559
Huang MJ, W Wang, HM Leung, CY Chan, WK Liu, MH Wong, KC Cheung	2012	Mercury levels in road dust and household TSP/PM2.5 related to concentrations in hair in Guangzhou, China	Ecotoxicology and Environmental Safety	81		27-35
Lai SC, KF Ho, YY Zhang, SC Lee, Y Huang, SC Zou	2010	Characteristics of Residential Indoor Carbonaceous Aerosols: A Case Study in Guangzhou, Pearl River Delta Region	Aerosol and Air Quality Research	10	5	472-478
Li CL, JM Fu, GY Sheng, XH Bi, YM Hao, XM Wang, BX Mai	2005	Vertical distribution of PAHs in the indoor and outdoor PM2.5 in Guangzhou, China	Building and Environment	40	3	329-341
Li TX, SZ Cao, DL Fan, YQ	2016	Household concentrations and personal exposure of	Science of the Total Environment	548		43628

Zhang, BB Wang, XG Zhao, BP Leaderer, GF Shen, YW		PM2.5 among urban residents using different cooking fuels				
Liu H, ZW Zhang, NJ Wen, C Wang	2018	Determination and risk assessment of airborne endotoxin concentrations in a university campus	Journal of Aerosol Science	115		146-157
Liu J, Y Man, Y Liu	2014	Temporal variability of PM10 and PM2.5 inside and outside a residential home during 2014 Chinese Spring Festival in Zhengzhou, China	Natural Hazards	73	3	2149-2154
Liu JJ, XL Dai, XD Li, SS Jia, JJ Pei, YX Sun, DY Lai, X Shen, HJ Sun, HG Yin, KL	2018	Indoor air quality and occupants' ventilation habits in China: Seasonal measurement and long-term monitoring	Building and Environment	142		119-129
Liu S, J Chen, Q Zhao, XM Song, DQ Shao, K Meliefste, YP Du, J Wang, M Wang, T	2018	Cardiovascular benefits of short-term indoor air filtration intervention in elderly living in Beijing: An extended analysis of BIAPSY study	Environmental Research	167		632-638
Liu Z, K Cheng, H Li, G Cao, D Wu, Y Shi	2018	Exploring the potential relationship between indoor air quality and the concentration of airborne culturable fungi: a combined experimental and neural network modeling study	Environ Sci Pollut Res Int	25	4	3510-3517
Liu ZJ, AG Li, ZP Hu, HF Sun	2014	Study on the potential relationships between indoor culturable fungi, particle load and children respiratory health in Xi'an, China	Building and Environment	80		105-114
Niu X, B Guinot, J Cao, H Xu, J Sun	2015	Particle size distribution and air pollution patterns in three urban environments in Xi'an, China	Environ Geochem Health	37	5	801-12
Niu X, Ho KF, Hu T, Sun J, Duan J, Huang Y, et al.	2019	Characterization of chemical components and cytotoxicity effects of indoor and outdoor fine	Environmental Science and Pollution Research	26	31	31913-23

		particulate matter (PM2.5) in Xi'an, China.				
Pei JJ, Dong CB, Liu JJ.	2019	Operating behavior and corresponding performance of portable air cleaners in residential buildings, China.	Building and Environment	147		473-81
Qi M, X Zhu, W Du, YL Chen, YC Chen, TB Huang, XL Pan, QR Zhong, X Sun,	2017	Exposure and health impact evaluation based on simultaneous measurement of indoor and ambient PM2.5 in Haidian, Beijing	Environmental Pollution	220		704-712
Shao ZJ, J Bi, ZW Ma, JN Wang	2017	Seasonal trends of indoor fine particulate matter and its determinants in urban residences in Nanjing, China	Building and Environment	125		319-325
Sun Y, Hou J, Cheng R, Sheng Y, Zhang X, Sundell J.	2019	Indoor air quality, ventilation and their associations with sick building syndrome in Chinese homes.	Energy and Buildings	197	15	112-9
Tong X, Chen XC, Chuang HC, Cao JJ, Ho SSH, Lui KH, et al.	2019	Characteristics and cytotoxicity of indoor fine particulate matter (PM2.5) and PM2.5-bound polycyclic aromatic hydrocarbons (PAHs) in Hong Kong.	Air Quality, Atmosphere & Health	12	10	Epub, Pages
Tong XN, B Wang, WT Dai, JJ Cao, SSH Ho, TCY Kwok, KH Lui, CM Lo, KF Ho	2018	Indoor air pollutant exposure and determinant factors controlling household air quality for elderly people in Hong Kong	Air Quality Atmosphere and Health	11	6	695-704
Wan MP, CL Wu, GN Szeto, TC Chan, CYH Chao	2011	Ultrafine particles, and PM2.5 generated from cooking in homes	Atmospheric Environment	45	34	6141-6148
Wang D, Wang P, Wang YW, Zhang WW, Zhu CF, Sun HZ, et al.	2019	Temporal variations of PM2.5-bound organophosphate flame retardants in different microenvironments in Beijing, China, and implications	Science of the Total Environment	666		226-34

		for human exposure.				
Wang F, D Meng, X Li, J Tan	2016	Indoor-outdoor relationships of PM2.5 in four residential dwellings in winter in the Yangtze River Delta, China	Environ Pollut	215		280-289
Wang F, YY Zhou, D Meng, MM Han, CQ Jia	2018	Heavy metal characteristics and health risk assessment of PM2.5 in three residential homes during winter in Nanjing, China	Building and Environment	143		339-348
Wang JN, Y Zhang	1990	CO and particle pollution of indoor air in Beijing and its elemental analysis	Biomed Environ Sci	3	2	132-8
Wang ZJ, QW Xue, YC Ji, ZY Yu	2018	Indoor environment quality in a low-energy residential building in winter in Harbin	Building and Environment	135		194-201
Wang ZQ, JJ Liu	2018	Spring-time PM2.5 elemental analysis and polycyclic aromatic hydrocarbons measurement in High-rise residential buildings in Chongqing and Xian, China	Energy and Buildings	173		623-633
Wu YJ, Li GY, Yang Y, An TC.	2019	Pollution evaluation and health risk assessment of airborne toxic metals in both indoors and outdoors of the Pearl River Delta, China.	Environmental Research	179		Epub
Xiang RB, JB Song, Y Zhu, W Guang	2016	Indoor Air Quality in Kitchens in Rural China	Environmental Engineering Science	33	9	699-704
Xiao Y, LN Wang, MZ Yu, TT Shui, L Liu, J Liu	2018	Characteristics of indoor/outdoor PM2.5 and related carbonaceous species in a typical severely cold city in China during heating season	Building and Environment	129		54-64

Xie YY, B Zhao	2018	Chemical composition of outdoor and indoor PM2.5 collected during haze events: Transformations and modified source contributions resulting from outdoor-to-indoor transport	Indoor Air	28	6	828-839
Xu F, W Tang, W Zhang, L Liu, K Lin	2016	Levels, distributions and correlations of polybrominated diphenyl ethers in air and dust of household and workplace in Shanghai, China: implication for daily human exposure	Environ Sci Pollut Res Int	23	4	3229-38
Yang F, Lau CF, Tong VWT, Zhang KK, Westerdahl D, Ng S, et al.	2019	Assessment of personal integrated exposure to fine particulate matter of urban residents in Hong Kong.	Journal of the Air & Waste Management Association	69	1	47-57
Yang YB, L Liu, CY Xu, N Li, Z Liu, Q Wang, DQ Xu	2018	Source Apportionment and Influencing Factor Analysis of Residential Indoor PM2.5 in Beijing	International Journal of Environmental Research and Public Health	15	4	
Yang Z, Shen J, Gao Z.	2018	Ventilation and Air Quality in Student Dormitories in China: A Case Study during Summer in Nanjing.	International journal of environmental research and public health	15	7	Epub
Yin H, Liu C, Zhang L, Li A, Ma Z.	2019	Measurement and evaluation of indoor air quality in naturally ventilated residential buildings.	Indoor and Built Environment	28	10	1307-23
Zhan Y, K Johnson, C Norris, MM Shafer, MH Bergin, Y Zhang, J Zhang, JJ Schauer	2018	The influence of air cleaners on indoor particulate matter components and oxidative potential in residential households in Beijing	Sci Total Environ	626		507-518
Zhang HB, JC Xie, H Yoshino, U Yanagi, K Hasegawa, N Kagi, ZW Lian	2016	Thermal and environmental conditions in Shanghai households: Risk factors for childhood health	Building and Environment	104		35-46
Zhang YQ, SZ Cao, XY Xu, J Qiu, MX Chen, D Wang, DH Guan, CY Wang, X Wang,	2016	Metals compositions of indoor PM2.5, health risk assessment, and birth outcomes in Lanzhou, China	Environmental Monitoring and Assessment	188	6	

Zhao L, JJ Liu, JL Ren	2018	Impact of various ventilation modes on IAQ and energy consumption in Chinese dwellings: First long-term monitoring study in Tianjin, China	Building and Environment	143		99-106
Zhou R, S Li, Y Zhou, A Haug	2000	Comparison of environmental tobacco smoke concentrations and mutagenicity for several indoor environments	Mutat Res	465		191-200
Zhou, Z., Liu, Y., Yuan, J., Zuo, J., Chen, G., Xu, L. and Rameezdeen, R.	2016	Indoor PM2. 5 concentrations in residential buildings during a severely polluted winter: A case study in Tianjin, China	Renewable and Sustainable Energy Reviews	64		372-381
Zhu SW, W Cai, H Yoshino, U Yanagi, K Hasegawa, N Kagi, MQ Chen	2015	Primary pollutants in schoolchildren's homes in Wuhan, China	Building and Environment	93		41-53
	Japan	PM2.5				
Ma CJ, GU Kang, CH Kang	2015	Properties of Indoor Particles Collected in Japanese Homes	Asian Journal of Atmospheric Environment	9	1	31-38
Michikawa T, S Nakai, H Nitta, K Tamura	2014	Validity of using annual mean particulate matter concentrations as measured at fixed site in assessing personal exposure: An exposure assessment study in Japan	Science of the Total Environment	466		673-680
Ohura T, T Noda, T Amagai, M Fusaya	2005	Prediction of personal exposure to PM2.5 and carcinogenic polycyclic aromatic hydrocarbons by their concentrations in residential microenvironments	Environmental Science & Technology	39	15	5592-5599
Sugiyama T, T Amagai, H Matsushita, M Soma	1999	Size distribution of indoor airborne particulates collected by a low-flow rate cascade impactor	Indoor and Built Environment	8	6	361-369

Yoda Y, K Tamura, M Shima	2017	Airborne endotoxin concentrations in indoor and outdoor particulate matter and their predictors in an urban city	Indoor Air	27	5	955-964
	Mongolia	PM2.5				
Barn P, E Gombojav, C Ochir, B Laagan, B Beejin, G Naidan, B Boldbaatar, J	2018	The effect of portable HEPA filter air cleaners on indoor PM2.5 concentrations and second hand tobacco smoke exposure among pregnant women in Ulaanbaatar, Mongolia: The UGAAR randomized controlled trial	Science of the Total Environment	615		1379-1389
	South Korea	PM2.5				
Choi DH, DH Kang	2017	Infiltration of Ambient PM2.5 through Building Envelope in Apartment Housing Units in Korea	Aerosol and Air Quality Research	17	2	598-607
Hwang Y, K Lee	2018	Contribution of microenvironments to personal exposures to PM10 and PM2.5 in summer and winter	Atmospheric Environment	175		192-198
Kang K, Kim H, Kim DD, Lee YG, Kim T.	2019	Characteristics of cooking-generated PM10 and PM2.5 in residential buildings with different cooking and ventilation types.	Science of The Total Environment	668		56-66
Lee CH, BK Lee, IB Oh, JH Lee, CS Sim, Y Kim	2012	Indoor Air Quality in Elementary School Children's Homes in Ulsan: Comparison between Groups with and without Allergic Rhinitis	Journal of Korean Society for Atmospheric Environment	28	4	365-373
Lee JY, C Kim, J Kim, SH Ryu, GN Bae	2018	Exposure Assessments for Children in Homes and in Daycare Centers to NO2, PMs and Black Carbon	Asian Journal of Atmospheric Environment	12	3	204-214

Lim S, J Kim, T Kim, K Lee, W Yang, S Jun, S Yu	2012	Personal exposures to PM2.5 and their relationships with microenvironmental concentrations	Atmospheric Environment	47		407-412
Oh HJ, Jeong NN, Sohn JR, Kim J.	2019	Personal exposure to indoor aerosols as actual concern: Perceived indoor and outdoor air quality, and health performances.	Building and Environment	165		106403
Park JS, NY Jee, JW Jeong	2014	Effects of types of ventilation system on indoor particle concentrations in residential buildings	Indoor Air	24	6	629-638
	Taiwan	PM2.5				
Chang LT, GB Hong, SP Weng, HC Chuang, TY Chang, CW Liu, WY Chuang,	2018	Indoor ozone levels, houseplants and peak expiratory flow rates among healthy adults in Taipei, Taiwan	Environ Int			
Chi, M.C., Guo, S.E., Hwang, S.L., Chou, C.T., Lin, C.M. and Lin, Y.C	2017	Exposure to Indoor Particulate MatterWorsens the Symptoms and Acute Exacerbations in Chronic Obstructive Pulmonary Disease Patients of Southwestern Taiwan: A Pilot Study	International journal of environmental research and public health	14	1	4
Chuang HC, KF Ho, LY Lin, TY Chang, GB Hong, CM Ma, IJ Liu, KJ Chuang	2017	Long-term indoor air conditioner filtration and cardiovascular health: A randomized crossover intervention study	Environment International	106		91-96
Hsu NY, CC Lee, JY Wang, YC Li, HW Chang, CY Chen, CG Bornehag, PC Wu, J	2012	Predicted risk of childhood allergy, asthma, and reported symptoms using measured phthalate exposure in dust and urine	Indoor Air	22	3	186-199
Huang YL, HW Chen, BC Han, CW Liu, HC Chuang, LY Lin, KJ Chuang	2014	Personal Exposure to Household Particulate Matter, Household Activities and Heart Rate Variability among Housewives	Plos One	9	3	

Li CS	1994	RELATIONSHIPS OF INDOOR OUTDOOR INHALABLE AND RESPIRABLE PARTICLES IN DOMESTIC ENVIRONMENTS	Science of the Total Environment	151	3	205-211
Li CS, CH Lin	2003	Carbon profile of residential indoor PM1 and PM2.5 in the subtropical region	Atmospheric Environment	37	7	881-888
Lin LY, CY Lin, YC Lin, KJ Chuang	2009	The effects of indoor particles on blood pressure and heart rate among young adults in Taipei, Taiwan	Indoor Air	19	6	482-488
Lin LY, HC Chuang, IJ Liu, HW Chen, KJ Chuang	2013	Reducing indoor air pollution by air conditioning is associated with improvements in cardiovascular health among the general population	Science of the Total Environment	463		176-181
Lin LY, HW Chen, TL Su, GB Hong, LC Huang, KJ Chuang	2011	The effects of indoor particle exposure on blood pressure and heart rate among young adults: An air filtration-based intervention study	Atmospheric Environment	45	31	5540-5544
Yen YC, Yang CY, Mena KD, Cheng YT, Chen PS.	2019	Cooking/Window Opening and Associated Increases of Indoor PM2.5 and NO2 Concentrations of Children's Houses in Kaohsiung, Taiwan.	Applied Sciences-Basel	9	20	Epub
Yen YC, Yang CY, Mena KD, Cheng YT, Yuan CS, Chen PS.	2019	Jumping on the bed and associated increases of PM10, PM2.5, PM1, airborne endotoxin, bacteria, and fungi concentrations.	Environ Pollut	245		799-809

Eastern Europe PM2.5

Czechia PM2.5

Branis M, J Kolomaznikova	2010	Monitoring of long-term personal exposure to fine particulate matter (PM2.5)	Air Quality Atmosphere and Health	3	4	235-243

Branis M, P Rezacova, M Lazaridis	2014	The effect of source type and source strength on inhaled mass of particulate matter during episodic indoor activities	Indoor and Built Environment	23	8	1106-1116
Braniš, M., Hovorka, J., Řezáčcová, P., Domasová, M., & Lazaridis, M.	2005	Effect of Indoor and Outdoor Sources on Particulate Matter Concentration in a Naturally Ventilated Flat (URBAN-AEROSOL Project-Prague)	Indoor Built Environment	14	3	307-12
Hanninen OO, E Lebret, V Ilacqua, K Katsouyanni, F Kunzli, RJ Sram, M Jantunen	2004	Infiltration of ambient PM2.5 and levels of indoor generated non-ETS PM2.5 in residences of four European cities	Atmospheric Environment	38	37	6411-6423
Kousa A, L Oglesby, K Koistinen, N Kunzli, M Jantunen	2002	Exposure chain of urban air PM2.5 - associations between ambient fixed site, residential outdoor, indoor, workplace and personal exposures in four European cities in the EXPOLIS-study	Atmospheric Environment	36	18	3031-3039

Hungary PM2.5

Szirtesi K, A Angyal, Z Szoboszlai, E Furu, Z Torok, T Igaz, Z Kertesz	2018	Airborne Particulate Matter: An Investigation of Buildings with Passive House Technology in Hungary	Aerosol and Air Quality Research	18	5	1282-1293

Lithuania PM2.5

Du L, T Prasauskas, V Leivo, M Turunen, M Pekkonen, M Kiviste, A Aaltonen, D	2015	Assessment of indoor environmental quality in existing multi-family buildings in North-East Europe	Environ Int	79		74-84
Prasauskas T, D Martuzevicius, E Krugly, D Ciuzas, I Stasiulaitiene, R	2014	Spatial and temporal variations of particulate matter concentrations in multifamily apartment buildings	Building and Environment	76		43755

North Macedonia PM2.5

Vilcekova S, IZ Apostoloski, L Meciarova, EK Burdova, J Kiselak	2017	Investigation of Indoor Air Quality in Houses of Macedonia	Int J Environ Res Public Health	14	1	

Poland PM2.5

Ścibor M, Balcerzak B, Galbarczyk A, Targosz N, Jasienska G.	2019	Are we safe inside? Indoor air quality in relation to outdoor concentration of PM10 and PM2.5 and to characteristics of homes.	Sustainable Cities and Society	48		101537

Slovakia PM2.5

Brauer M, F Hruba, E Mihalikova, E Fabianova, P Miskovic, A Plzikova, M	2000	Personal exposure to particles in Banska Bystrica, Slovakia	Journal of Exposure Analysis and Environmental Epidemiology	10	5	478-487
Meciarova L, S Vilcekova, EK Burdova, J Kiselak	2017	Factors Effecting the Total Volatile Organic Compound (TVOC) Concentrations in Slovak Households	International Journal of Environmental Research and Public Health	14	12	

North America PM2.5

Canada PM2.5

Akom JB, Sadick AM, Issa MH, Rashwan S, Duhoux M.	2018	The indoor environmental quality performance of green low-income single-family housing.	Journal of Green Building	13	2	98-120
Allen RW, C Carlsten, B Karlen, S Leckie, S van Eeden, S Vedal, I Wong, M	2011	An Air Filter Intervention Study of Endothelial Function among Healthy Adults in a Woodsmoke-impacted Community	American Journal of Respiratory and Critical Care Medicine	183	9	1222-1230
Allen RW, S Leckie, G Millar, M Brauer	2009	The impact of wood stove technology upgrades on indoor residential air quality	Atmospheric Environment	43	37	5908-5915

Barn P, T Larson, M Noullett, S Kennedy, R Copes, M Brauer	2008	Infiltration of forest fire and residential wood smoke: an evaluation of air cleaner effectiveness	Journal of Exposure Science and Environmental Epidemiology	18	5	503-511
Clark NA, RW Allen, P Hystad, L Wallace, SD Dell, R Foty, E Dabek-	2010	Exploring Variation and Predictors of Residential Fine Particulate Matter Infiltration	International Journal of Environmental Research and Public Health	7	8	3211-3224
Crump KS	2000	Manganese exposures in Toronto during use of the gasoline additive, methylcyclopentadienyl manganese tricarbonyl	Journal of Exposure Analysis and Environmental Epidemiology	10	3	227-239
Evans GJ, A Peers, K Sabaliauskas	2008	Particle dose estimation from frying in residential settings	Indoor Air	18	6	499-510
Ghoshdastidar AJ, Z Hu, Y Nazarenko, PA Ariya	2018	Exposure to nanoscale and microscale particulate air pollution prior to mining development near a northern indigenous community in Quebec, Canada	Environ Sci Pollut Res Int	25	9	8976-8988
Heroux ME, N Clark, K Van Ryswyk, R Mallick, NL Gilbert, I Harrison, K	2010	Predictors of Indoor Air Concentrations in Smoking and Non-Smoking Residences	International Journal of Environmental Research and Public Health	7	8	3080-3099
Jeong CH, Salehi S, Wu J, North ML, Kim JS, Chow CW, et al.	2019	Indoor measurements of air pollutants in residential houses in urban and suburban areas: Indoor versus ambient concentrations.	Science of The Total Environment	693		133446
Kajbafzadeh M, M Brauer, B Karlen, C Carlsten, S van Eeden, RW Allen	2015	The impacts of traffic-related and woodsmoke particulate matter on measures of cardiovascular health: a HEPA filter intervention study	Occupational and Environmental Medicine	72	6	394-400
Kearney J, L Wallace, M MacNeill, ME Heroux, W Kindzierski, A Wheeler	2014	Residential infiltration of fine and ultrafine particles in Edmonton	Atmospheric Environment	94		793-805

Loo CK, RG Foty, AJ Wheeler, JD Miller, G Evans, DM Stieb, SD Dell	2010	Do questions reflecting indoor air pollutant exposure from a questionnaire predict direct measure of exposure in owner-occupied houses?	Int J Environ Res Public Health	7	8	3270-97
MacNeill M, J Kearney, L Wallace, M Gibson, ME Heroux, J Kuchta, JR	2014	Quantifying the contribution of ambient and indoor-generated fine particles to indoor air in residential environments	Indoor Air	24	4	362-375
Miller JD, Dugandzic R, Frescura AM, Salares V	2007	Indoor-and outdoor-derived contaminants in urban and rural homes in Ottawa, Ontario, Canada	Journal of the Air & Waste Management Association	57	3	297-302
Miller, J.D., Dugandzic, R., Frescura, A.M. and Salares, V.	2007	Indoor- and Outdoor-Derived Contaminants in Urban and Rural Homes in Ottawa, Ontario, Canada	Journal of the Air & Waste Management Association	57	7	297-302
Rasmussen PE, C Levesque, M Chenier, HD Gardner	2018	Contribution of metals in resuspended dust to indoor and personal inhalation exposures: Relationships between PM10 and settled dust	Building and Environment	143		513-522
Smargiassi A, M Baldwin, C Pilger, R Dugandzic, M Brauer	2005	Small-scale spatial variability of particle concentrations and traffic levels in Montreal: a pilot study	Science of the Total Environment	338	3	243-251
Weichenthal S, G Mallach, R Kulka, A Black, A Wheeler, H You, M St-Jean,	2013	A randomized double-blind crossover study of indoor air filtration and acute changes in cardiorespiratory health in a First Nations community	Indoor Air	23	3	175-184
Wheeler AJ, LA Wallace, J Kearney, K Van Ryswyk, HY You, R Kulka, JR Brook, XH	2011	Personal, Indoor, and Outdoor Concentrations of Fine and Ultrafine Particles Using Continuous Monitors in Multiple Residences	Aerosol Science and Technology	45	9	1078-1089

	USA	PM2.5				
Abraham ME	1999	Microanalysis of indoor aerosols and the impact of a Compact High-Efficiency Particulate air (HEPA) filter system	Indoor Air-International Journal of Indoor Air Quality and Climate	9	1	33-40
Abt E, HH Suh, G Allen, P Koutrakis	2000	Characterization of indoor particle sources: A study conducted in the metropolitan Boston area	Environmental Health Perspectives	108	1	35-44
Adgate JL, G Ramachandran, GC Pratt, LA Waller, K Sexton	2002	Spatial and temporal variability in outdoor, indoor, and personal PM2.5 exposure	Atmospheric Environment	36	20	3255-3265
Adgate JL, Ramachandran G, Pratt GC, Waller LA, Sexton K	2003	Longitudinal variability in outdoor, indoor, and personal PM2.5 exposure in healthy non-smoking adults	Atmospheric Environment	37	7	993-1002
Allen R, T Larson, L Sheppard, L Wallace, LJ Liu	2003	Use of real-time light scattering data to estimate the contribution of infiltrated and indoor-generated particles to indoor air	Environ Sci Technol	37	16	3484-92
Arhami M, A Polidori, RJ Delfino, T Tjoa, C Sioutas	2009	Associations between Personal, Indoor, and Residential Outdoor Pollutant Concentrations: Implications for Exposure Assessment to Size-Fractionated Particulate Matter	Journal of the Air & Waste Management Association	59	4	392-404
Arku RE, G Adamkiewicz, J Vallarino, JD Spengler, DE Levy	2015	Seasonal variability in environmental tobacco smoke exposure in public housing developments	Indoor Air	25	1	13-20
Balmes JR, M Cisternas, PJ Quinlan, L Trupin, FW Lurmann, PP Katz, PD Blanc	2014	Annual average ambient particulate matter exposure estimates, measured home particulate matter, and hair nicotine are associated with respiratory outcomes in adults with asthma	Environmental Research	129		43475

Baxter LK, JE Clougherty, CJ Paciorek, RJ Wright, JI Levy	2007	Predicting residential indoor concentrations of nitrogen dioxide, fine particulate matter, and elemental carbon using questionnaire and geographic information system based data	Atmospheric Environment	41	31	6561-6571
Beamer PI, AJ Sugeng, MD Kelly, N Lothrop, W Klimecki, ST Wilkinson, M	2014	Use of dust fall filters as passive samplers for metal concentrations in air for communities near contaminated mine tailings	Environmental Science-Processes & Impacts	16	6	1275-1281
Belli AJ, S Bose, N Aggarwal, C DaSilva, S Thapa, L Grammer, LM	2016	Indoor particulate matter exposure is associated with increased black carbon content in airway macrophages of former smokers with COPD	Environmental Research	150		398-402
Berry D, G Mainelis, D Fennell	2007	Effect of an ionic air cleaner on indoor/outdoor particle ratios in a residential environment	Aerosol Science and Technology	41	3	315-328
Breen MS, TC Long, BD Schultz, RW Williams, J Richmond-Bryant, M	2015	Air Pollution Exposure Model for Individuals (EMI) in Health Studies: Evaluation for Ambient PM2.5 in Central North Carolina	Environmental Science & Technology	49	24	14184-
Breysse PN, TJ Buckley, D Williams, CM Beck, SJ Jo, B Merriman, S	2005	Indoor exposures to air pollutants and allergens in the homes of asthmatic children in inner-city Baltimore	Environmental Research	98	2	167-176
Brown KW, JA Sarnat, HH Suh, BA Coull, JD Spengler, P Koutrakis	2008	Ambient site, home outdoor and home indoor particulate concentrations as proxies of personal exposures	Journal of Environmental Monitoring	10	9	1041-1051
Brown, D.R., Alderman, N., Weinberger, B., Lewis, C., Bradley, J. and Curtis, L.	2014	Outdoor wood furnaces create significant indoor particulate pollution in neighboring homes	Inhalation toxicology	26	10	628-635

Brugge D, J Vallarino, L Ascolillo, ND Osgood, S Steinbach, J Spengler	2003	Comparison of multiple environmental factors for asthmatic children in public housing	Indoor Air	13	1	18-27
Butz AM, EC Matsui, P Breysse, J Curtin-Brosnan, P Eggleston, G Diette, D	2011	A Randomized Trial of Air Cleaners and a Health Coach to Improve Indoor Air Quality for Inner-City Children With Asthma and Secondhand Smoke Exposure	Archives of Pediatrics & Adolescent Medicine	165	8	741-748
Butz AM, P Breysse, C Rand, J Curtin-Brosnan, P Eggleston, GB Diette, D	2011	Household Smoking Behavior: Effects on Indoor Air Quality and Health of Urban Children with Asthma	Maternal and Child Health Journal	15	4	460-468
Chan WR, JM Logue, X Wu, NE Klepeis, WJ Fisk, F Noris, BC Singer	2018	Quantifying fine particle emission events from time-resolved measurements: Method description and application to 18 California low-income apartments	Indoor Air	28	1	89-101
Chen Q, LM Hildemann	2009	Size-Resolved Concentrations of Particulate Matter and Bioaerosols Inside versus Outside of Homes	Aerosol Science and Technology	43	7	699-713
Chen Q, LM Hildemann	2009	The Effects of Human Activities on Exposure to Particulate Matter and Bioaerosols in Residential Homes	Environmental Science & Technology	43	13	4641-4646
Cheng KC, HK Park, AO Tetteh, D Zheng, NT Ouellette, KC Nadeau, LM	2016	Mixing and sink effects of air purifiers on indoor PM2.5 concentrations: A pilot study of eight residential homes in Fresno, California	Aerosol Science and Technology	50	8	835-845
Clayton CA, RL Perritt, ED Pellizzari, KW Thomas, RW Whitmore, LA Wallace, H	1993	PARTICLE TOTAL EXPOSURE ASSESSMENT METHODOLOGY (PTEAM) STUDY - DISTRIBUTIONS OF AEROSOL AND ELEMENTAL CONCENTRATIONS IN PERSONAL, INDOOR, AND OUTDOOR AIR SAMPLES IN A SOUTHERN CALIFORNIA COMMUNITY	Journal of Exposure Analysis and Environmental Epidemiology	3	2	227-250

Clougherty JE, EA Houseman, JI Levy	2011	Source apportionment of indoor residential fine particulate matter using land use regression and constrained factor analysis	Indoor Air	21	1	53-66
Colton MD, P MacNaughton, J Vallarino, J Kane, M Bennett-Fripp, JD	2014	Indoor Air Quality in Green Vs Conventional Multifamily Low-Income Housing	Environmental Science & Technology	48	14	7833-7841
Coombs KC, GL Chew, C Schaffer, PH Ryan, C Brokamp, SA Grinshpun, G	2016	Indoor air quality in green-renovated vs. non-green low-income homes of children living in a temperate region of US (Ohio)	Science of the Total Environment	554		178-185
Cox J, K Isiugo, P Ryan, SA Grinshpun, M Yermakov, C Desmond, R Jandarov, S	2018	Effectiveness of a portable air cleaner in removing aerosol particles in homes close to highways	Indoor Air	28	6	818-827
Delfino RJ, N Staimer, T Tjoa	2011	Personal endotoxin exposure in a panel study of school children with asthma	Environmental Health	10		
Delfino RJ, PJ Quintana, J Floro, VM Gastanaga, BS Samimi, MT Kleinman, LJ	2004	Association of FEV1 in asthmatic children with personal and microenvironmental exposure to airborne particulate matter	Environ Health Perspect	112	8	932-41
Diette GB, NN Hansel, TJ Buckley, J Curtin-Brosnan, PA Eggleston, EC Matsui,	2007	Home indoor pollutant exposures among inner-city children with and without asthma	Environmental Health Perspectives	115	11	1665-1669
Doll SC, EL Davison, BR Painting	2016	Weatherization impacts and baseline indoor environmental quality in low income single-family homes	Building and Environment	107		181-190
Du L, S Batterman, E Parker, C Godwin, JY Chin, A O'Toole, T Robins, W	2011	Particle concentrations and effectiveness of free-standing air filters in bedrooms of children with asthma in Detroit, Michigan	Building and Environment	46	11	2303-2313

Eggleston PA, A Butz, C Rand, J Curtin-Brosnan, S Kanchanaraksa, L Swartz, P	2005	Home environmental intervention in inner-city asthma: a randomized controlled clinical trial	Annals of Allergy Asthma & Immunology	95	6	518-524
Escobedo LE, WM Champion, N Li, LD Montoya	2014	Indoor air quality in Latino homes in Boulder, Colorado	Atmospheric Environment			92 69-75
Evans GF, RV Highsmith, LS Sheldon, JC Suggs, RW Williams, RB Zweidinger, JP	2000	The 1999 Fresno particulate matter exposure studies: Comparison of community, outdoor, and residential PM mass measurements	Journal of the Air & Waste Management Association	50	11	1887-1896
Fleisch AF, Rokoff LB, Garshick E, Grady ST, Chipman JW, Baker ER, et	2019	Residential wood stove use and indoor exposure to PM2.5 and its components in Northern New England.	Journal of Exposure Science and Environmental Epidemiology			Epub
Frey SE, H Destaillats, S Cohn, S Ahrentzen, MP Fraser	2014	Characterization of indoor air quality and resident health in an Arizona senior housing apartment building	Journal of the Air & Waste Management Association	64	11	1251-1259
Frey SE, H Destaillats, S Cohn, S Ahrentzen, MP Fraser	2015	The effects of an energy efficiency retrofit on indoor air quality	Indoor Air	25	2	210-219
Geller MD, MH Chang, C Sioutas, BD Ostro, MJ Lipsett	2002	Indoor/outdoor relationship and chemical composition of fine and coarse particles in the southern California deserts	Atmospheric Environment	36	6	1099-1110
Gould CF, SN Chillrud, D Phillips, MS Perzanowski, D Hernandez	2018	Soot and the city: Evaluating the impacts of Clean Heat policies on indoor/outdoor air quality in New York City apartments	Plos One	13	6	

Habre R, B Coull, E Moshier, J Godbold, A Grunin, A Nath, W Castro,	2014	Sources of indoor air pollution in New York City residences of asthmatic children	Journal of Exposure Science and Environmental Epidemiology	24	3	269-278
Hansel NN, MC McCormack, AJ Belli, EC Matsui, RD Peng, C Aloe, L	2013	In-Home Air Pollution Is Linked to Respiratory Morbidity in Former Smokers with Chronic Obstructive Pulmonary Disease	American Journal of Respiratory and Critical Care Medicine	187	10	1085-1090
Hart JE, Grady ST, Laden F, Coull BA, Koutrakis P, Schwartz JD, et al.	2018	Effects of Indoor and Ambient Black Carbon and [Formula: see text] on Pulmonary Function among Individuals with COPD.	Environ Health Perspectives	126	12	127008
Hart JF, TJ Ward, TM Spear, RJ Rossi, NN Holland, BG Loushin	2011	Evaluating the Effectiveness of a Commercial Portable Air Purifier in Homes with Wood Burning Stoves: A Preliminary Study	Journal of Environmental and Public Health			
Henderson DE, JB Milford, SL Miller	2005	Prescribed burns and wildfires in Colorado: Impacts of mitigation measures on indoor air particulate matter	Journal of the Air & Waste Management Association	55	10	1516-1526
Holm SM, J Balmes, D Gillette, K Hartin, E Seto, D Lindeman, D Polanco, E Fong	2018	Cooking behaviors are related to household particulate matter exposure in children with asthma in the urban East Bay Area of Northern California	Plos One	13	6	
Howard-Reed C, AW Rea, MJ Zufall, JM Burke, RW Williams, JC Suggs, LS	2000	Use of a continuous nephelometer to measure personal exposure to particles during the US Environmental Protection Agency Baltimore and Fresno panel studies	Journal of the Air & Waste Management Association	50	7	1125-1132
Hsu SI, K Ito, M Kendall, M Lippmann	2012	Factors affecting personal exposure to thoracic and fine particles and their components	Journal of Exposure Science and Environmental Epidemiology	22	5	439-447

Huang SD, J Lawrence, CM Kang, J Li, M Martins, P Vokonas, DR Gold, J	2018	Road proximity influences indoor exposures to ambient fine particle mass and components	Environmental Pollution	243		978-987
Hunt A, JA Crawford, PF Rosenbaum, JL Abraham	2011	Levels of household particulate matter and environmental tobacco smoke exposure in the first year of life for a cohort at risk for asthma in urban Syracuse, NY	Environment International	37	7	1196-1205
Isiugo K, Jandarov R, Cox J, Ryan P, Newman N, Grinshpun SA, et al.	2019	Indoor particulate matter and lung function in children.	Science of the Total Environment	663		408-17
Jansen KL, TV Larson, JQ Koenig, TF Mar, C Fields, J Stewart, M Lippmann	2005	Associations between health effects and particulate matter and black carbon in subjects with respiratory disease	Environmental Health Perspectives	113	12	1741-1746
Johnson R, J Schmid, S Dinakaran, R Seifert	2005	Use of simulink for dynamic air quality modeling in interior Alaska	Journal of Cold Regions Engineering	19	1	43542
Jung KH, D Torrone, S Lovinsky-Desir, M Perzanowski, J Bautista, JR	2017	Short-term exposure to PM2.5 and vanadium and changes in asthma gene DNA methylation and lung function decrements among urban children	Respiratory Research	18		
Jung KH, K Bernabe, K Moors, B Yan, SN Chillrud, R Whyatt, D Camann, PL	2011	Effects of Floor Level and Building Type on Residential Levels of Outdoor and Indoor Polycyclic Aromatic Hydrocarbons, Black Carbon, and Particulate Matter in New York City	Atmosphere	2	2	96-109
Jung KH, MM Patel, K Moors, PL Kinney, SN Chillrud, R Whyatt, L	2010	Effects of heating season on residential indoor and outdoor polycyclic aromatic hydrocarbons, black carbon, and particulate matter in an urban birth cohort	Atmospheric Environment	44	36	4545-4552

Kamens R, CT Lee, R Wiener, D Leith	1991	A STUDY TO CHARACTERIZE INDOOR PARTICLES IN 3 NONSMOKING HOMES	Atmospheric Environment Part a-General Topics	25		939-948
Kang CM, D Gold, P Koutrakis	2014	Downwind O-3 and PM2.5 speciation during the wildfires in 2002 and 2010	Atmospheric Environment	95		511-519
Keeler GJ, JT Dvonch, FY Yip, EA Parker, BA Israel, FJ Marsik, M Morishita, JA	2002	Assessment of personal and community-level exposures to particulate matter among children with asthma in Detroit, Michigan, as part of Community Action Against Asthma (CAAA)	Environmental Health Perspectives	110		173-181
Khurshid SS, JA Siegel, KA Kinney	2016	Particulate reactive oxygen species on total suspended particles - measurements in residences in Austin, Texas	Indoor Air	26	6	953-963
Khurshid, S.S., Siegel, J.A. and Kinney, KA	2014	Indoor particulate reactive oxygen species concentrations	Environmental research	132		46-53
Kilburg-Basnyat B, TM Peters, SS Perry, PS Thorne	2016	Electrostatic dust collectors compared to inhalable samplers for measuring endotoxin concentrations in farm homes	Indoor Air	26	5	724-733
King, B.A., Travers, M.J., Cummings, K.M., Mahoney, M.C. and Hyland, A.J.	2010	Secondhand smoke transfer in multiunit housing	Nicotine & Tobacco Research	12	11	1133-1141
Kinney PL, SN Chillrud, S Ramstrom, J Ross, JD Spengler	2002	Exposures to multiple air toxics in New York City	Environmental Health Perspectives	110		539-546

Koehler K, Good N, Wilson A, Molter A, Moore BF, Carpenter T, et al.	2019	The Fort Collins commuter study: Variability in personal exposure to air pollutants by microenvironment.	Indoor Air: Interational Journal of Indoor Environment and Health	29	2	231-41
Kopperud RJ, AR Ferro, LM Hildemann	2004	Outdoor versus indoor contributions to indoor particulate matter (PM) determined by mass balance methods	Journal of the Air & Waste Management Association	54	9	1188-1196
Lachenmyer C, GM Hidy	2000	Urban measurements of outdoor-indoor PM25 concentrations and personal exposure in the deep south. Part I. Pilot study of mass concentrations for nonsmoking subjects	Aerosol Science and Technology	32	1	34-51
Leaderer BP, L Naeher, T Jankun, K Balenger, TR Holford, C Toth, J Sullivan,	1999	Indoor, outdoor, and regional summer and winter concentrations of PM10, PM2.5, SO42-, H+, NH4+, NO3-, NH3, and nitrous acid in homes with and without kerosene space heaters	Environmental Health Perspectives	107	3	223-231
Leaderer BP, P Koutrakis, SLK Briggs, J Rizzuto	1994	THE MASS CONCENTRATION AND ELEMENTAL COMPOSITION OF INDOOR AEROSOLS IN SUFFOLK AND ONONDAGA COUNTIES, NEW-YORK	Indoor Air-International Journal of Indoor Air Quality and Climate	4	1	23-34
LeBouf R, L Yesse, A Rossner	2008	Seasonal and diurnal variability in airborne mold from an indoor residential environment in northern New York	Journal of the Air & Waste Management Association	58	5	684-692
Levy JI, T Dumyahn, JD Spengler	2002	Particulate matter and polycyclic aromatic hydrocarbon concentrations in indoor and outdoor microenvironments in Boston, Massachusetts	Journal of Exposure Analysis and Environmental Epidemiology	12	2	104-114

Lewis CW	1991	Sources of air pollutants indoors: VOC and fine particulate species	J Expo Anal Environ Epidemiol	1	1	31-44
Li WW, H Paschold, H Morales, J Chianelli	2003	Correlations between short-term indoor and outdoor PM concentrations at residences with evaporative coolers	Atmospheric Environment	37	19	2691-2703
Linn WS, H Gong, KW Clark, KR Anderson	1999	Day-to-day particulate exposures and health changes in Los Angeles area residents with severe lung disease	Journal of the Air & Waste Management Association	49		108-115
Liu LJ, M Box, D Kalman, J Kaufman, J Koenig, T Larson, T Lumley, L	2003	Exposure assessment of particulate matter for susceptible populations in Seattle	Environ Health Perspect	111	7	909-18
Long CM, HH Suh, L Kobzik, PJ Catalano, YY Ning, P Koutrakis	2001	A pilot investigation of the relative toxicity of indoor and outdoor fine particles: In vitro effects of endotoxin and other particulate properties	Environmental Health Perspectives	109	10	1019-1026
Long CM, HH Suh, P Koutrakis	2000	Characterization of indoor particle sources using continuous mass and size monitors	Journal of the Air & Waste Management Association	50	7	1236-1250
Long CM, HH Suh, PJ Catalano, P Koutrakis	2001	Using time- and size-resolved particulate data to quantify indoor penetration and deposition behavior	Environmental Science & Technology	35	10	2089-2099
MacNaughton P, G Adamkiewicz, RE Arku, J Vallarino, DE Levy	2016	The impact of a smoke-free policy on environmental tobacco smoke exposure in public housing developments	Science of the Total Environment	557		676-680

Maestas MM, Brook RD, Ziemba RA, Li FY, Crane RC, Klaver ZM, et al.	2019	Reduction of personal PM2.5 exposure via indoor air filtration systems in Detroit: an intervention study.	Journal of Exposure Science and Environmental Epidemiology	29	4	484-90
Martuzevicius D, SA Grinshpun, T Lee, SH Hu, P Biswas, T Reponen, G	2008	Traffic-related PM2.5 aerosol in residential houses located near major highways: Indoor versus outdoor concentrations	Atmospheric Environment	42	27	6575-6585
McCormack MC, AJ Belli, D Waugh, EC Matsui, RD Peng, DL Williams, L Paulin,	2016	Respiratory Effects of Indoor Heat and the Interaction with Air Pollution in Chronic Obstructive Pulmonary Disease	Annals of the American Thoracic Society	13	12	2125-2131
McCormack MC, PN Breysse, NN Hansel, EC Matsui, ES Tonorezos, J	2008	Common household activities are associated with elevated particulate matter concentrations in bedrooms of inner-city Baltimore pre-school children	Environmental Research	106	2	148-155
Militello-Hourigan RE, SL Miller	2018	The impacts of cooking and an assessment of indoor air quality in Colorado passive and tightly constructed homes	Building and Environment	144		573-582
Miller SL, P Scaramella, J Campe, CW Goss, S Diaz-Castillo, E Hendrikson, C	2009	An assessment of indoor air quality in recent Mexican immigrant housing in Commerce City, Colorado	Atmospheric Environment	43	35	5661-5667
Min KT, Lundrigan P, Sward K, Collingwood SC, Patwari N.	2018	Smart home air filtering system: A randomized controlled trial for performance evaluation.	Smart Health			62-75
Mukerjee S, WD Ellenson, RG Lewis, RK Stevens, MC Somerville, DS Shadwick,	1997	An environmental scoping study in the Lower Rio Grande Valley of Texas .3. Residential microenvironmental monitoring for air, house dust, and soil	Environment International	23	5	657-673

Na K, DR Cocker	2005	Organic and elemental carbon concentrations in fine particulate matter in residences, schoolrooms, and outdoor air in Mira Loma, California	Atmospheric Environment	39	18	3325-3333
Noonan CW, TJ Ward, W Navidi, L Sheppard, M Bergauff, C Palmer	2011	Assessing the impact of a wood stove replacement program on air quality and children's health	Res Rep Health Eff Inst		162	3-37;
Olson DA, J Turlington, RV Duvall, SR Vicdow, CD Stevens, R Williams	2008	Indoor and outdoor concentrations of organic and inorganic molecular markers: Source apportionment of PM2.5 using low-volume samples	Atmospheric Environment	42	8	1742-1751
Patton AP, L Calderon, YY Xiong, ZC Wang, J Senick, MS Allacci, D Plotnik, R	2016	Airborne Particulate Matter in Two Multi-Family Green Buildings: Concentrations and Effect of Ventilation and Occupant Behavior	International Journal of Environmental Research and Public Health	13	1	
Pavilonis BT, TR Anthony, PT O'Shaughnessy, MJ Humann, JA Merchant, G	2013	Indoor and outdoor particulate matter and endotoxin concentrations in an intensely agricultural county	Journal of Exposure Science and Environmental Epidemiology	23	3	299-305
Pellizzari ED, CA Clayton, CE Rodes, RE Mason, LL Piper, B Fort, G Pfeifer, D Lynam	2001	Particulate matter and manganese exposures in Indianapolis, Indiana	Journal of Exposure Analysis and Environmental Epidemiology	11	6	423-440
Rodes CE, PA Lawless, JW Thornburg, RW Williams, CW Croghan	2010	DEARS particulate matter relationships for personal, indoor, outdoor, and central site settings for a general population	Atmospheric Environment	44	11	1386-1399
Rojas-Bracho L, HH Suh, P Koutrakis	2000	Relationships among personal, indoor, and outdoor fine and coarse particle concentrations for individuals with COPD	Journal of Exposure Analysis and Environmental Epidemiology	10	3	294-306

Russo ET, TE Hulse, G Adamkiewicz, DE Levy, L Bethune, J Kane, M Reid,	2015	Comparison of Indoor Air Quality in Smoke-Permitted and Smoke-Free Multiunit Housing: Findings From the Boston Housing Authority	Nicotine & Tobacco Research	17	3	316-322
Sarnat SE, BA Coull, PA Ruiz, P Koutrakis, HH Suh	2006	The influences of ambient particle composition and size on particle infiltration in Los Angeles, CA, residences	Journal of the Air & Waste Management Association	56	2	186-196
Sawant AA, K Na, XN Zhu, DR Cocker	2004	Chemical characterization of outdoor PM2.5 and gas-phase compounds in Mira Loma, California	Atmospheric Environment	38	0	5517-5528
Sawant AA, K Na, XN Zhu, K Cocker, S Butt, C Song, DR Cocker	2004	Characterization of PM2.5 and selected gas-phase compounds at multiple indoor and outdoor sites in Mira Loma, California	Atmospheric Environment	38	37	6269-6278
Simons E, J Curtin-Brosnan, T Buckley, P Breysse, PA Eggleston	2007	Indoor environmental differences between inner city and suburban homes of children with asthma	Journal of Urban Health-Bulletin of the New York Academy of Medicine	84	4	577-590
Singer BC, RZ Pass, WW Delp, DM Lorenzetti, RL Maddalena	2017	Pollutant concentrations and emission rates from natural gas cooking burners without and with range hood exhaust in nine California homes	Building and Environment	122		215-229
Thomas NM, Calderón L, Senick J, Sorensen-Allacci M, Plotnik D, Guo M, et al.	2019	Investigation of indoor air quality determinants in a field study using three different data streams.	Building and Environment	154		281-95
Tryner J, Quinn C, Windom BC, Volckens J.	2019	Design and evaluation of a portable PM2.5 monitor featuring a low-cost sensor in line with an active filter sampler.	Environmental Science-Processes & Impacts	21	8	1403-15
Tunno BJ, S Kyra Naumoff, L Cambal, S Tripathy, F Holguin, P Lioy, JE	2015	Indoor air sampling for fine particulate matter and black carbon in industrial communities in Pittsburgh	Sci Total Environ	536		108-115

Turpin BJ, CP Weisel, M Morandi, S Colome, T Stock, S Eisenreich, B	2007	Relationships of Indoor, Outdoor, and Personal Air (RIOPA): part II. Analyses of concentrations of particulate matter species	Res Rep Health Eff Inst			1-77;
Van Deusen A, A Hyland, MJ Travers, C Wang, C Higbee, BA King, T Alford,	2009	Secondhand smoke and particulate matter exposure in the home	Nicotine & Tobacco Research	11	6	635-641
Vette A, J Burke, G Norris, M Landis, S Batterman, M Breen, V Isakov, T Lewis, MI	2013	The Near-Road Exposures and Effects of Urban Air Pollutants Study (NEXUS): Study design and methods	Science of the Total Environment	448		38-47
Wallace L, R Williams, A Rea, C Croghan	2006	Continuous weeklong measurements of personal exposures and indoor concentrations of fine particles for 37 health-impaired North Carolina residents for up to four seasons	Atmospheric Environment	40	3	399-414
Wallace LA, H Mitchell, GT O'Connor, L Neas, M Lippmann, M Kattan, J	2003	Particle concentrations in inner-city homes of children with asthma: the effect of smoking, cooking, and outdoor pollution	Environ Health Perspect	111	9	1265-72
Wallace LA, SJ Emmerich, C Howard-Reed	2004	Source strengths of ultrafine and fine particles due to cooking with a gas stove	Environmental Science & Technology	38	8	2304-2311
Wallace, L; Williams, R; Suggs, J; Sheldon, L; Zweidinger, R; Rea, A;	2003	Exposure of High Risk Subpopulations to Particles: Final Report (APM 21)	EPA report (peer reviewed)	0	0	
Ward T, C Noonan	2008	Results of a residential indoor PM(2.5) sampling program before and after a woodstove changeout	Indoor Air	18	5	408-415
Ward T, J Boulafentis, J Simpson, C Hester, T Moliga, K Warden, C Noonan	2011	Lessons learned from a woodstove changeout on the Nez Perce Reservation	Science of the Total Environment	409	4	664-670

Ward TJ, EO Semmens, E Weiler, S Harrar, CW Noonan	2017	Efficacy of interventions targeting household air pollution from residential wood stoves	Journal of Exposure Science and Environmental Epidemiology	27	1	64-71
Waring MS, JA Siegel	2011	The effect of an ion generator on indoor air quality in a residential room	Indoor Air	21	4	267-276
Williams R, AG Rappold, M Case, M Schmitt, S Stone, P Jones, J Thornburg, RB	2012	Multi-pollutant exposures in an asthmatic cohort	Atmospheric Environment	61		244-252
Williams R, J Creason, R Zweidinger, R Watts, L Sheldon, C Shy	2000	Indoor, outdoor, and personal exposure monitoring of particulate air pollution: the Baltimore elderly epidemiology-exposure pilot study	Atmospheric Environment	34	24	4193-4204
Williams R, J Suggs, A Rea, K Leovic, A Vette, C Croghan, L Sheldon, C	2003	The Research Triangle Park particulate matter panel study: PM mass concentration relationships	Atmospheric Environment	37	38	5349-5363
Williams R, J Suggs, R Zweidinger, G Evans, J Creason, R Kwok, C Rodes,	2000	The 1998 Baltimore particulate matter Epidemiology-Exposure Study: Part 1. Comparison of ambient, residential outdoor, indoor and apartment particulate matter monitoring	Journal of Exposure Analysis and Environmental Epidemiology	10	6	518-532
Williams, R.O.N., Rea, A., Vette, A., Croghan, C., Whitaker, D., Stevens, C.,	2009	The design and field implementation of the Detroit Exposure and Aerosol Research Study	Journal of Exposure Science and Environmental Epidemiology	19	7	643-659
Wu CF, RJ Delfino, JN Floro, PJE Quintana, BS Samimi, MT Kleinman, RW Allen, LJS	2005	Exposure assessment and modeling of particulate matter for asthmatic children using personal nephelometers	Atmospheric Environment	39	19	3457-3469
Xiong Y, U Krogmann, G Mainelis, LA Rodenburg, CJ Andrews	2015	Indoor air quality in green buildings: A case-study in a residential high-rise building in the northeastern United States	J Environ Sci Health A Tox Hazard Subst Environ Eng	50	3	225-42

Zamora ML, JC Pulczinski, N Johnson, R Garcia-Hernandez, A Rule, G	2018	Maternal exposure to PM2.5 in south Texas, a pilot study	Science of the Total Environment			1497-1507
Zanobetti A, PH Stone, FE Speizer, JD Schwartz, BA Coull, HH Suh, BD Nearing,	2009	T-Wave Alternans, Air Pollution and Traffic in High-Risk Subjects	American Journal of Cardiology	104	5	665-670
Zhang T, Chillrud SN, Yang Q, Pitiranggon M, Ross J, Perera F, et al.	2019	Characterizing peak exposure of secondhand smoke using a real-time PM2.5 monitor.	Indoor Air: Interational Journal of Indoor Environment and Health			Epub

Oceania PM2.5

Australia PM2.5

Hargreaves M, S Parappukkaran, L Morawska, J Hitchins, CR	2003	A pilot investigation into associations between indoor airborne fungal and non-biological particle concentrations in residential houses in Brisbane, Australia	Science of the Total Environment	312		89-101
Jones J, S Stick, P Dingle, P Franklin	2007	Spatial variability of particulates in homes: Implications for infant exposure	Science of the Total Environment	376		317-323
Molloy SB, M Cheng, IE Galbally, MD Keywood, SJ Lawson, JC Powell, R	2012	Indoor air quality in typical temperate zone Australian dwellings	Atmospheric Environment	54		400-407
Morawska L, CR He, J Hitchins, K Mengersen, D Gilbert	2003	Characteristics of particle number and mass concentrations in residential houses in Brisbane, Australia	Atmospheric Environment	37	30	4195-4203

South America PM2.5

Brazil PM2.5

Segalin B, P Kumar, K Micadei, A Fornaro, FLT Goncalves	2017	Size-segregated particulate matter inside residences of elderly in the Metropolitan Area of Sao Paulo, Brazil	Atmospheric Environment	148		139-151

Chile PM2.5

Barraza F, H Jorquera, G Valdivia, LD Montoya	2014	Indoor PM2.5 in Santiago, Chile, spring 2012: Source apportionment and outdoor contributions	Atmospheric Environment	94		692-700
Barria RM, M Calvo, P Pino	2016	Indoor air pollution by fine particulate matter in the homes of newborns	Revista Chilena De Pediatria-Chile	87	5	343-350
Bravo-Linares C, L Ovando-Fuentealba, S Orellana-Donoso, S Gatica, F	2016	Source identification, apportionment and toxicity of indoor and outdoor PM2.5 airborne particulates in a region characterised by wood burning	Environmental Science-Processes & Impacts	18	5	575-589
Burgos S, P Ruiz, R Koifman	2013	Changes to indoor air quality as a result of relocating families from slums to public housing	Atmospheric Environment	70		179-185
Reyes R, Schueftan A, Ruiz C, González AD.	2019	Controlling air pollution in a context of high energy poverty levels in southern Chile: Clean air but colder houses?	Energy Policy	124		301-11
Rojas-Bracho L, HH Suh, P Oyola, P Koutrakis	2002	Measurements of children's exposures to particles and nitrogen dioxide in Santiago, Chile	Science of the Total Environment	287	3	249-264

Ruiz PA, C Toro, J Caceres, G Lopez, P Oyola, P Koutrakis	2010	Effect of Gas and Kerosene Space Heaters on Indoor Air Quality: A Study in Homes of Santiago, Chile	Journal of the Air & Waste Management Association	60	1	98-108
	Ecuador	PM2.5				
Raysoni AU, RX Armijos, MM Weigel, T Montoya, P Eschanique, M Racines,	2016	Assessment of indoor and outdoor PM species at schools and residences in a high-altitude Ecuadorian urban center	Environmental Pollution	214		668-679
	Peru	PM2.5				
Underhill LJ, S Bose, DL Williams, KM Romero, G Malpartida, PN Breysse, EM	2015	Association of Roadway Proximity with Indoor Air Pollution in a Peri-Urban Community in Lima, Peru	International Journal of Environmental Research and Public Health	12	10	13466-
**South Asia**		PM2.5				
	Bangladesh	PM2.5				
Akther T, Ahmed M, Shohel M, Ferdousi FK, Salam A.	2019	Particulate matters and gaseous pollutants in indoor environment and Association of ultra-fine particulate matters (PM1) with lung function.		26	6	5475-84.
Doshi S, BJ Silk, D Dutt, M Ahmed, AL Cohen, TH Taylor, WA Brooks, D	2015	Household-level risk factors for influenza among young children in Dhaka, Bangladesh: a case-control study	Tropical Medicine & International Health	20	6	719-729
Gurley, E.S., Salje, H., Homaira, N., Ram, P.K., Haque, R., Petri Jr, W.A.,	2013	Seasonal concentrations and determinants of indoor particulate matter in a low-income community in Dhaka, Bangladesh	Environmental research	121	0	11-16
	India	PM2.5				
Curto A, Donaire-Gonzalez	2018	Performance of low-cost monitors to assess	Environmental Research	163		53-63

D, Barrera-Gómez J, Marshall JD,		household air pollution.				
Elf JL, Kinikar A, Khadse S, Mave V, Suryavanshi N, Gupte N, et al.	2018	Sources of household air pollution and their association with fine particulate matter in low-income urban homes in India article.	Journal of Exposure Science and Environmental Epidemiology	28	4	400-10
Khillare PS, R Pandey, S Balachandran	2004	Characterisation of indoor PM10 in residential areas of Delhi	Indoor and Built Environment	13	2	139-147
Kulshreshtha P, M Khare, P Seetharaman	2008	Indoor air quality assessment in and around urban slums of Delhi city, India	Indoor Air	18	6	488-98
Kulshrestha A, DD Massey, J Masih, A Taneja	2014	Source Characterization of Trace Elements in Indoor Environments at Urban, Rural and Roadside Sites in a Semi Arid Region of India	Aerosol and Air Quality Research	14	6	1738-1751
Kulshrestha A, DS Bisht, J Masih, D Massey, S Tiwari, A Taneja	2009	Chemical characterization of water-soluble aerosols in different residential environments of semi aridregion of India	Journal of Atmospheric Chemistry	62	2	121-138
Kumar P	2001	Characterisation of indoor respirable dust in a locality of Delhi, India	Indoor and Built Environment	10	2	95-102
Lawrence A, N Fatima	2014	Urban air pollution & its assessment in Lucknow City - The second largest city of North India	Science of the Total Environment	488		449-457
Massey D, A Kulshrestha, J Masih, A Taneja	2012	Seasonal trends of PM10, PM5.0, PM2.5 & PM1.0 in indoor and outdoor environments of residential homes located in North-Central India	Building and Environment	47		223-231

Massey D, J Masih, A Kulshrestha, M Habil, A Teneja	2009	Indoor/outdoor relationship of fine particles less than 2.5 mu m (PM2.5) in residential homes locations in central Indian region	Building and Environment	44	10	2037-2045
Priyamvada H, C Priyanka, RK Singh, M Akila, R Ravikrishna, SS Gunthe	2018	Assessment of PM and bioaerosols at diverse indoor environments in a southern tropical Indian region	Building and Environment	137		215-225
Roy R, R Jan, S Yadav, MH Vasave, PG Satsangi	2016	Study of metals in radical-mediated toxicity of particulate matter in indoor environments of Pune, India	Air Quality Atmosphere and Health	9	6	669-680
Satsangi PG, S Yadav, AS Pipal, N Kumbhar	2014	Characteristics of trace metals in fine (PM2.5) and inhalable (PM10) particles and its health risk assessment along with in-silico approach in indoor environment of India	Atmospheric Environment	92		384-393
Singh P, R Saini, A Taneja	2014	Physicochemical characteristics of PM2.5: Low, middle, and high-income group homes in Agra, India- a case study	Atmospheric Pollution Research	5	3	352-360

Nepal PM2.5

Shakya KM, RE Peltier, H Shrestha, RM Byanju	2017	Measurements of TSP, PM10, PM2.5, BC, and PM chemical composition from an urban residential location in Nepal	Atmospheric Pollution Research	8	6	1123-1131

Pakistan PM2.5

Abbas M, S Razzaq, R Shahzadi, A Rahman, F Manzoor, Z Ali	2015	DETERMINATION OF INDOOR AND OUTDOOR AIR QUALITY UNDER DIFFERENT VENTILATION CONDITIONS IN A RESIDENTIAL AREA OF LAHORE, PAKISTAN	Journal of Animal and Plant Sciences	25	3	672-676

Ali Z, F Naz, ZA Nasir, S Sidra, I Colbeck	2015	PARTICULATE POLLUTION IN URBAN RESIDENTIAL BUILT ENVIRONMENTS DURING WINTER AND SUMMER SEASONS IN LAHORE, PAKISTAN	Journal of Animal and Plant Sciences	25	3	660-665
Colbeck I, Sidra S, Ali Z, Ahmed S, Nasir ZA.	2019	Spatial and temporal variations in indoor air quality in Lahore, Pakistan.	International Journal of Environmental Science and Technology	16	6	2565-72
Nasir, Z. A., Colbeck, I., Ali, Z., & Ahmad, S	2013	Indoor particulate matter in developing countries: a case study in Pakistan and potential intervention strategies	Environmental Research Letters	8	2	024002

South East Asia PM2.5

Indonesia PM2.5

Azhar K, I Dharmayanti, I Mufida	2016	The Indoor Average Level of PM2,5 and ARI Among Children Under Five in Kelurahan Kayuringin Jaya, Kota Bekasi 2014	Media Penelitian Dan Pengembangan Kesehatan	26	1	45-52
Pramitha E, Haryanto B.	2019	Effect of Exposure to 2.5 mum Indoor Particulate Matter on Adult Lung Function in Jakarta.	Osong Public Health and Research Perspectives	10	2	51-5

Malaysia PM2.5

Nazariah, S.S.N., Juliana, J. and Abdah, M.A.	2013	Interleukin-6 via sputum induction as biomarker of inflammation for indoor particulate matter among primary school children in Klang Valley, Malaysia	Global journal of health science	5	4	93

Singapore PM2.5

Balasubramanian R, P Nainar, A Rajasekar	2012	Airborne bacteria, fungi, and endotoxin levels in residential microenvironments: a case study	Aerobiologia	28	3	375-390

Balasubramanian R, SS Lee	2007	Characteristics of indoor aerosols in residential homes in urban locations: A case study in Singapore	Journal of the Air & Waste Management Association	57	8	981-990

Thailand PM2.5

Tsai FC, KR Smith, N Vichit-Vadakan, BD Ostro, LG Chestnut, N Kungskulniti	2000	Indoor/outdoor PM10 and PM2.5 in Bangkok, Thailand	Journal of Exposure Analysis and Environmental Epidemiology	10	1	15-26

West Asia & North Africa PM2.5

Egypt PM2.5

Abdel-Salam MM	2013	Indoor particulate matter in urban residences of Alexandria, Egypt	J Air Waste Manag Assoc	63	8	956-62
Abdel-Salam MMM	2015	Investigation of PM2.5 and carbon dioxide levels in urban homes	Journal of the Air & Waste Management Association	65	8	930-936
Wagdi D, K Tarabieh, MN Abou Zeid	2018	Indoor air quality index for preoccupancy assessment	Air Quality Atmosphere and Health	11	4	445-458

Iran PM2.5

Hassanvand MS, K Naddafi, H Kashani, S Faridi, N Kunzli, R Nabizadeh, F	2017	Short-term effects of particle size fractions on circulating biomarkers of inflammation in a panel of elderly subjects and healthy young adults	Environmental Pollution	223		695-704
Hassanvand MS, K Naddafi, S Faridi, M Arhami, R Nabizadeh, MH Sowlat, Z	2014	Indoor/outdoor relationships of PM10, PM2.5, and PM1 mass concentrations and their water-soluble ions in a retirement home and a school dormitory	Atmospheric Environment	82		375-382
Mirhoseini SH, M Nikaeen,	2016	Assessment of Airborne Particles in Indoor	Aerosol and Air Quality Research	16	8	1903-1910

K Satoh, K Makimura		Environments: Applicability of Particle Counting for Prediction of Bioaerosol Concentrations				
	Israel	PM2.5				
Dobson R, Rosen LJ, Semple S.	2019	Monitoring secondhand tobacco smoke remotely in real-time: A simple low-cost approach.	Tobacco Induced Diseases	17		18
Jodeh S, AR Hasan, J Amarah, F Judeh, R Salghi, H Lgaz, W Jodeh	2018	Indoor and outdoor air quality analysis for the city of Nablus in Palestine: seasonal trends of PM10, PM5.0, PM2.5, and PM1.0 of residential homes	Air Quality Atmosphere and Health	11	2	229-237
Krasnov H, I Katra, V Novack, A Vodonos, MD Friger	2015	Increased indoor PM concentrations controlled by atmospheric dust events and urban factors	Building and Environment	87		169-176
Rosen, L., Zucker, D., Hovell, M., Brown, N., Ram, A. and Myers, V.	2015	Feasibility of Measuring Tobacco Smoke Air Pollution in Homes: Report from a Pilot Study	International journal of environmental research and public health	12	12	15129-
	Kuwait	PM2.5				
Yassin MF, BEY AlThaqeb, EAE Al-Mutiri	2012	Assessment of indoor PM2.5 in different residential environments	Atmospheric Environment	56		65-68
	Turkey	PM2.5				
Mentese S, AY Rad, M Arisoy, G Gullu	2012	Multiple comparisons of organic, microbial, and fine particulate pollutants in typical indoor environments: Diurnal and seasonal variations	Journal of the Air & Waste Management Association	62	12	1380-1393
Pekey B, ZB Bozkurt, H Pekey, G Dogan, A Zararsiz, N Efe, G Tuncel	2010	Indoor/outdoor concentrations and elemental composition of PM10/PM2.5 in urban/industrial areas of Kocaeli City, Turkey	Indoor Air	20	2	112-125

United Arab Emirates PM2.5

Weitzman M, AH Yusufali, F Bali, MJR Vilcassim, S Gandhi, R Peltier, A Nadas,	2017	Effects of hookah smoking on indoor air quality in homes	Tobacco Control	26	5	586-591
Yeatts KB, M El-Sadig, D Leith, W Kalsbeek, F Al-Maskari, D Couper, WE	2012	Indoor Air Pollutants and Health in the United Arab Emirates	Environmental Health Perspectives	120	5	687-694

Western Europe PM2.5

Belgium PM2.5

Buczynska AJ, A Krata, R Van Grieken, A Brown, G Polezer, K De Wael, S	2014	Composition of PM2.5 and PM1 on high and low pollution event days and its relation to indoor air quality in a home for the elderly	Science of the Total Environment	490		134-143
Jacobs L, A Buczynska, C Walgraeve, A Delcloo, S Potgieter-Vermaak, R Van	2012	Acute changes in pulse pressure in relation to constituents of particulate air pollution in elderly persons	Environmental Research	117		60-67
Stranger M, SS Potgieter-Vermaak, R Van Grieken	2009	Particulate matter and gaseous pollutants in residences in Antwerp, Belgium	Science of the Total Environment	407	3	1182-1192

Denmark PM2.5

Jantzen K, P Moller, DG Karottki, Y Olsen, G Beko, G Clausen, LG Hersoug, S Loft	2016	Exposure to ultrafine particles, intracellular production of reactive oxygen species in leukocytes and altered levels of endothelial progenitor cells	Toxicology	359		43787
Karottki DG, G Beko, G Clausen, AM Madsen, ZJ Andersen, A Massling, M	2014	Cardiovascular and lung function in relation to outdoor and indoor exposure to fine and ultrafine particulate matter in middle-aged subjects	Environment International	73		372-381

Karottki DG, M Spilak, M Frederiksen, ZJ Andersen, AM Madsen, M Ketzel, A	2015	Indoor and Outdoor Exposure to Ultrafine, Fine and Microbiologically Derived Particulate Matter Related to Cardiovascular and Respiratory Effects in a Panel of Elderly Urban Citizens	International Journal of Environmental Research and Public Health	12	2	1667-1686
Olsen Y, DG Karottki, DM Jensen, G Beko, BU Kjeldsen, G Clausen, LG	2014	Vascular and lung function related to ultrafine and fine particles exposure assessed by personal and indoor monitoring: a cross-sectional study	Environmental Health	13		
Raaschou-Nielsen O, M Sorensen, O Hertel, BLK Chawes, N Vissing, K	2011	Predictors of indoor fine particulate matter in infants' bedrooms in Denmark	Environmental Research	111	1	87-93
Sorensen M, S Loft, HV Andersen, OR Nielsen, LT Skovgaard, LE Knudsen,	2005	Personal exposure to PM2.5, black smoke and NO2 in Copenhagen: relationship to bedroom and outdoor concentrations covering seasonal variation	Journal of Exposure Analysis and Environmental Epidemiology	15	5	413-422
Spilak MP, GD Karottki, B Kolarik, M Frederiksen, S Loft, L Gunnarsen	2014	Evaluation of building characteristics in 27 dwellings in Denmark and the effect of using particle filtration units on PM2.5 concentrations	Building and Environment	73		55-63

Finland PM2.5

de Hartog JJ, JG Ayres, A Karakatsani, A Analitis, H Brink, K Hameri, RM	2010	Lung function and indicators of exposure to indoor and outdoor particulate matter among asthma and COPD patients	Occupational and Environmental Medicine	67	1	43506
Du L, T Prasauskas, V Leivo, M Turunen, M Pekkonen, M Kiviste, A Aaltonen, D	2015	Assessment of indoor environmental quality in existing multi-family buildings in North-East Europe	Environ Int	79		74-84
Hanninen OO, E Lebret, V Ilacqua, K Katsouyanni, F Kunzli, RJ Sram, M Jantunen	2004	Infiltration of ambient PM2.5 and levels of indoor generated non-ETS PM2.5 in residences of four European cities	Atmospheric Environment	38	37	6411-6423

Hoek G, G Kos, RM Harrison, J de Hartog, K Meliefste, H ten Brink, K	2008	Indoor-outdoor relationships of particle number and mass in four European cities	Atmospheric Environment	42	1	156-169
Janssen NAH, T Lanki, G Hoek, M Vallius, JJ de Hartog, R Van Grieken, J	2005	Associations between ambient, personal, and indoor exposure to fine particulate matter constituents in Dutch and Finnish panels of cardiovascular patients	Occupational and Environmental Medicine	62	12	868-877
Janssen, N. A., de Hartog, J. J., Hoek, G., Brunekreef, B., Lanki, T., Timonen, K. L., &	2000	Personal Exposure to Fine Particulate Matter in Elderly Subjects: Relation between Personal, Indoor, and Outdoor Concentrations	Journal of the Air & Waste Management Association	50	7	1133-1143
Kousa A, L Oglesby, K Koistinen, N Kunzli, M Jantunen	2002	Exposure chain of urban air PM2.5 - associations between ambient fixed site, residential outdoor, indoor, workplace and personal exposures in four European cities in the EXPOLIS-study	Atmospheric Environment	36	18	3031-3039
Montagne, D., Hoek, G., Nieuwenhuijsen, M., Lanki, T., Siponen, T., Portella, M.,	2014	Temporal associations of ambient PM2. 5 elemental concentrations with indoor and personal concentrations	Atmospheric Environment	86	0	203-211
Siponen T, Yli-Tuomi T, Tiittanen P, Taimisto P, Pekkanen J, Salonen RO, et	2019	Wood stove use and other determinants of personal and indoor exposures to particulate air pollution and ozone among elderly persons in a Northern Suburb.	Indoor Air: Interational Journal of Indoor Environment and Health	29	3	413-22
Sippula O, H Rintala, M Happo, P Jalava, K Kuuspalo, A Viren, A	2013	Characterization of Chemical and Microbial Species from Size-Segregated Indoor and Outdoor Particulate Samples	Aerosol and Air Quality Research	13	4	1212-U343
Yli-Tuomi T, T Lanki, G Hoek, B Brunekreef, J Pekkanen	2008	Determination of the sources of indoor PM(2.5) in Amsterdam and Helsinki	Environmental Science & Technology	42	12	4440-4446

	France		PM2.5				
Audi C, N Baiz, CN Maesano, O Ramousse, D Reboulleau, A Magnan, D	2017	Serum cytokine levels related to exposure to volatile organic compounds and PM2.5 in dwellings and workplaces in French farmers - a mechanism to explain nonsmoking COPD	International Journal of Chronic Obstructive Pulmonary Disease	12		1363-1374
Derbez M, G Wyart, E Le Ponner, O Ramalho, J Riberon, C Mandin	2018	Indoor air quality in energy-efficient dwellings: Levels and sources of pollutants	Indoor Air	28	2	318-338
Derbez, M, B Bruno, C Valerie, L Murielle, P Cecile, R Jacques, K Severine	2014	Indoor air quality and comfort in seven newly built, energy-efficient houses in France	Building and Environment	72		173-187
Hulin M, D Caillaud, I Annesi-Maesano	2010	Indoor air pollution and childhood asthma: variations between urban and rural areas	Indoor Air	20	6	502-514
Langer S, O Ramalho, M Derbez, J Riberon, S Kirchner, C Mandin	2016	Indoor environmental quality in French dwellings and building characteristics	Atmospheric Environment	128		82-91
Maesano CN, Caillaud D, Youssouf H, Banerjee S, Prud'Homme J, Audi C, et	2019	Indoor exposure to particulate matter and volatile organic compounds in dwellings and workplaces and respiratory health in French farmers.	Multidisciplinary Respiratory Medicine	14	1	eCollection
Mosqueron L, I Momas, Y Le Moullec	2002	Personal exposure of Paris office workers to nitrogen dioxide and fine particles	Occupational and Environmental Medicine	59	8	550-555
Nerriere E, D Zmirou-Navier, O Blanchard, I Momas, J Ladner, Y Le	2005	Can we use fixed ambient air monitors to estimate population long-term exposure to air pollutants? The case of spatial variability in the Genotox ER study	Environmental Research	97	1	32-42

Ramalho O, G Wyart, C Mandin, P Blondeau, PA Cabanes, N Leclerc, JU	2015	Association of carbon dioxide with indoor air pollutants and exceedance of health guideline values	Building and Environment	93		115-124

Germany PM2.5

Franck U, O Herbarth, S Roder, U Schlink, M Borte, U Diez, U Kramer, I Lehmann	2011	Respiratory effects of indoor particles in young children are size dependent	Science of the Total Environment	409	9	1621-1631
Salthammer T, T Schripp, S Wientzek, M Wensing	2014	Impact of operating wood-burning fireplace ovens on indoor air quality	Chemosphere	103		205-211
Santen M, M Wesselmann, U Fittschen, R Cremer, P Braun, A Ludecke, HJ	2009	Measurements of fine and ultrafine particles in indoor environment of living rooms	Gefahrstoffe Reinhaltung Der Luft	69	3	63-70

Greece PM2.5

Assimakopoulos VD, T Bekiari, S Pateraki, T Maggos, P Stamatis, P	2018	Assessing personal exposure to PM using data from an integrated indoor-outdoor experiment in Athens-Greece	Science of the Total Environment	636		1303-1320
de Hartog JJ, JG Ayres, A Karakatsani, A Analitis, H Brink, K Hameri, RM	2010	Lung function and indicators of exposure to indoor and outdoor particulate matter among asthma and COPD patients	Occupational and Environmental Medicine	67	1	43506
Diapouli E, A Chaloulakou, N Spyrellis	2008	Indoor and outdoor PM concentrations at a residential environment, in the Athens area	Global Nest Journal	10	2	201-208
Diapouli E, A Chaloulakou, N Spyrellis	2010	INDOOR/OUTDOOR PM LEVELS AND EC SURROGATE, AT TYPICAL MICROENVIRONMENTS IN THE ATHENS AREA	Global Nest Journal	12	1	43818

Hanninen OO, E Lebret, V Ilacqua, K Katsouyanni, F Kunzli, RJ Sram, M Jantunen	2004	Infiltration of ambient PM2.5 and levels of indoor generated non-ETS PM2.5 in residences of four European cities	Atmospheric Environment	38	37	6411-6423
Hoek G, G Kos, RM Harrison, J de Hartog, K Meliefste, H ten Brink, K	2008	Indoor-outdoor relationships of particle number and mass in four European cities	Atmospheric Environment	42	1	156-169
Kousa A, L Oglesby, K Koistinen, N Kunzli, M Jantunen	2002	Exposure chain of urban air PM2.5 - associations between ambient fixed site, residential outdoor, indoor, workplace and personal exposures in four European cities in the EXPOLIS-study	Atmospheric Environment	36	18	3031-3039
Mueller W, Steinle S, Parkka J, Parmes E, Liedes H, Kuijpers E, et al.	2020	Urban greenspace and the indoor environment: Pathways to health via indoor particulate matter, noise, and road noise annoyance.	Environmental Research	180		108850
Saraga DE, T Maggos, CG Helmis, J Michopoulos, JG Bartzis, C Vasilakos	2010	PM1 and PM2.5 ionic composition and VOCs measurements in two typical apartments in Athens, Greece: investigation of smoking contribution to indoor air concentrations	Environmental Monitoring and Assessment	167		321-331
Seleventi MK, DE Saraga, CG Helmis, K Bairachtari, C Vasilakos, T Maggos	2012	PM2.5 INDOOR/OUTDOOR RELATIONSHIP AND CHEMICAL COMPOSITION IN IONS AND OC/EC IN AN APARTMENT IN THE CENTER OF ATHENS	Fresenius Environmental Bulletin	21	11	3177-3183
Stamatelopoulou A, Asimakopoulos DN, Maggos T.	2019	Effects of PM, TVOCs and comfort parameters on indoor air quality of residences with young children.	Building and Environment	150		233-44
	Ireland	PM2.5				
Broderick A, M Byrne, S Armstrong, J Sheahan, AM Coggins	2017	A pre and post evaluation of indoor air quality, ventilation, and thermal comfort in retrofitted co-operative social housing	Building and Environment	122		126-133

Galea KS, JF Hurley, H Cowie, AL Shafrir, AS Jimenez, S Semple, JG	2013	Using PM2.5 concentrations to estimate the health burden from solid fuel combustion, with application to Irish and Scottish homes	Environmental Health	12		
Semple, S., Garden, C., Coggins, M., Galea, K.S., Whelan, P., Cowie, H.,	2012	Contribution of solid fuel, gas combustion, or tobacco smoke to indoor air pollutant concentrations in Irish and Scottish homes	Indoor air	22	3	212-223
	Italy	PM2.5				
Cattaneo A, C Peruzzo, G Garramone, P Urso, R Ruggeri, P Carrer, DM	2011	Airborne particulate matter and gaseous air pollutants in residential structures in Lodi province, Italy	Indoor Air	21	6	489-500
Drago G, C Perrino, S Canepari, S Ruggieri, L L'Abbate, V Longo, P	2018	Relationship between domestic smoking and metals and rare earth elements concentration in indoor PM2.5	Environmental Research	165		71-80
Frasca D, Marcoccia M, Tofful L, Simonetti G, Perrino C, Canepari S.	2018	Influence of advanced wood-fired appliances for residential heating on indoor air quality.	Chemosphere	211		62-71
Perrino C, L Tofful, S Canepari	2016	Chemical characterization of indoor and outdoor fine particulate matter in an occupied apartment in Rome, Italy	Indoor Air	26	4	558-570
Romagnoli P, C Balducci, M Perilli, M Gherardi, A Gordiani, C Gariazzo, MP	2014	Indoor PAHs at schools, homes and offices in Rome, Italy	Atmospheric Environment	92		51-59
Romagnoli, P.; Balducci, C.; Perilli, M.; Vichi, F.; Imperiali, A.; Cecinato, A	2016	Indoor air quality at life and work environments in Rome, Italy	Environmental Science and Pollution Research	23		3503-3516
Simoni M, A Scognamiglio,	2004	Indoor exposures and acute respiratory effects in two	Journal of Exposure Analysis and	14		S144-S152

L Carrozzi, S Baldacci, A Angino, F Pistelli, F Di Pede,		general population samples from a rural and an urban area in Italy	Environmental Epidemiology			
Simoni M, L Carrozzi, S Baldacci, A Scognamiglio, F Di Pede, T Sapigni, G Viegi	2002	The Po River Delta (North Italy) indoor epidemiological study: Effects of pollutant exposure on acute respiratory symptoms and respiratory function in adults	Archives of Environmental Health	57	2	130-136
Zauli-Sajani S, S Rovelli, A Trentini, D Bacco, S Marchesi, F Scotto, C	2018	Higher health effects of ambient particles during the warm season: The role of infiltration factors	Science of the Total Environment	627		67-77
	Netherlands	PM2.5				
de Hartog JJ, JG Ayres, A Karakatsani, A Analitis, H Brink, K Hameri, RM	2010	Lung function and indicators of exposure to indoor and outdoor particulate matter among asthma and COPD patients	Occupational and Environmental Medicine	67	1	43506
Fischer PH, G Hoek, H van Reeuwijk, DJ Briggs, E Lebret, JH van Wijnen, S	2000	Traffic-related differences in outdoor and indoor concentrations of particles and volatile organic compounds in Amsterdam	Atmospheric Environment	34	22	3713-3722
Hoek G, G Kos, RM Harrison, J de Hartog, K Meliefste, H ten Brink, K	2008	Indoor-outdoor relationships of particle number and mass in four European cities	Atmospheric Environment	42	1	156-169
Janssen NAH, T Lanki, G Hoek, M Vallius, JJ de Hartog, R Van Grieken, J	2005	Associations between ambient, personal, and indoor exposure to fine particulate matter constituents in Dutch and Finnish panels of cardiovascular patients	Occupational and Environmental Medicine	62	12	868-877
Janssen, N. A., de Hartog, J. J., Hoek, G., Brunekreef, B., Lanki, T., Timonen, K. L., &	2000	Personal Exposure to Fine Particulate Matter in Elderly Subjects: Relation between Personal, Indoor, and Outdoor Concentrations	Journal of the Air & Waste Management Association	50	7	1133-1143

Montagne, D., Hoek, G., Nieuwenhuijsen, M., Lanki, T., Siponen, T., Portella, M.,	2014	Temporal associations of ambient PM2. 5 elemental concentrations with indoor and personal concentrations	Atmospheric Environment	86	0	203-211
Mueller W, Steinle S, Parkka J, Parmes E, Liedes H, Kuijpers E, et al.	2020	Urban greenspace and the indoor environment: Pathways to health via indoor particulate matter, noise, and road noise annoyance.	Environmental Research	180		108850
Yli-Tuomi T, T Lanki, G Hoek, B Brunekreef, J Pekkanen	2008	Determination of the sources of indoor PM(2.5) in Amsterdam and Helsinki	Environmental Science & Technology	42	12	4440-4446
	Norway	PM2.5				
Lazaridis M, V Aleksandropoulou, JE Hanssen, C Dye, K	2008	Inorganic and carbonaceous components in indoor/outdoor particulate matter in two residential houses in Oslo, Norway	Journal of the Air & Waste Management Association	58	3	346-356
Oie L, P Magnus, BV Johansen	1997	Suspended particulate matter in Norwegian dwellings in relation to fleece and shelf factors, domestic smoking, air exchange rate, and presence of hot wire convection heaters	Environment International	23	4	465-473
Rakkestad KE, CJ Dye, KE Yttri, JA Holme, JK Hongslo, PE Schwarze, R Becher	2007	Phthalate levels in Norwegian indoor air related to particle size fraction	Journal of Environmental Monitoring	9	12	1419-1425
Wyss AB, AC Jones, AK Bolling, GE Kissling, R Chartier, HJ Dahlman, CE	2016	Particulate Matter 2.5 Exposure and Self-Reported Use of Wood Stoves and Other Indoor Combustion Sources in Urban Nonsmoking Homes in Norway	Plos One	11	11	
	Portugal	PM2.5				
Almeida-Silva M, Pilou M, Housiadas C, Almeida SM.	2018	Internal dose of particles in the elderly—modeling based on aerosol measurements.	Environmental Science and Pollution Research	25	24	23645-56

Almeida-Silva, M., Wolterbeek, H.T. and Almeida, S.M	2014	Elderly exposure to indoor air pollutants	Atmospheric Environment	85		54-63
Belo J, Carreiro-Martins P, Papoila AL, Palmeiro T, Caires I, Alves M, et al.	2019	The impact of indoor air quality on respiratory health of older people living in nursing homes: spirometric and exhaled breath condensate assessments.	Journal of Environmental Science and Health Part a-Toxic/Hazardous Substances & Environmental Engineering	54	12	1153-8
Castro D, K Slezakova, C Delerue-Matos, MG Alvim-Ferraz, S Morais, MC Pereira	2010	CONTRIBUTION OF TRAFFIC AND TOBACCO SMOKE IN THE DISTRIBUTION OF POLYCYCLIC AROMATIC HYDROCARBONS ON OUTDOOR AND INDOOR PM2.5	Global Nest Journal	12	1	43535
Cerqueira M, D Marques, A Caseiro, C Pio	2010	Experimental evidence for a significant contribution of cellulose to indoor aerosol mass concentration	Atmospheric Environment	44	6	867-871
Madureira J, I Paciencia, J Cavaleiro-Rufo, E de Oliveira Fernandes	2016	Indoor pollutant exposure among children with and without asthma in Porto, Portugal, during the cold season	Environ Sci Pollut Res Int	23	20	20539-
Mendes A, AL Papoila, P Carreiro-Martins, S Bonassi, I Caires, T	2016	The impact of indoor air quality and contaminants on respiratory health of older people living in long-term care residences in Porto	Age and Ageing	45	1	136-142
Slezakova K, D Castro, C Delerue-Matos, S Morais, MD Pereira	2014	Levels and risks of particulate-bound PAHs in indoor air influenced by tobacco smoke: a field measurement	Environmental Science and Pollution Research	21	6	4492-4501
	Spain	PM2.5				
Dadvand P, A de Nazelle, M Triguero-Mas, A Schembari, M Cirach, E	2012	Surrounding Greenness and Exposure to Air Pollution During Pregnancy: An Analysis of Personal Monitoring Data	Environmental Health Perspectives	120	9	1286-1290

Fernandez E, M Ballbe, X Sureda, M Fu, E Salto, JM Martinez-Sanchez	2015	Particulate Matter from Electronic Cigarettes and Conventional Cigarettes: a Systematic Review and Observational Study	Curr Environ Health Rep	2	4	423-9
Montagne, D., Hoek, G., Nieuwenhuijsen, M., Lanki, T., Siponen, T., Portella, M.,	2014	Temporal associations of ambient PM2. 5 elemental concentrations with indoor and personal concentrations	Atmospheric Environment	86	0	203-211
Schembari A, M Triguero-Mas, A de Nazelle, P Dadvand, M Vrijheid, M	2013	Personal, indoor and outdoor air pollution levels among pregnant women	Atmospheric Environment	64		287-295
	Sweden	PM2.5				
Johannesson S, P Gustafson, P Molnar, L Barregard, G Sallsten	2007	Exposure to fine particles (PM2.5 and PM1) and black smoke in the general population: personal, indoor, and outdoor levels	Journal of Exposure Science and Environmental Epidemiology	17	7	613-624
Molnar P, P Gustafson, S Johannesson, J Boman, L Barregard, G Sallsten	2005	Domestic wood burning and PM2.5 trace elements: Personal exposures, indoor and outdoor levels	Atmospheric Environment	39	14	2643-2653
Wichmann J, T Lind, MAM Nilsson, T Bellander	2010	PM2.5, soot and NO2 indoor-outdoor relationships at homes, pre-schools and schools in Stockholm, Sweden	Atmospheric Environment	44	36	4536-4544
Wierzbicka A, M Bohgard, JH Pagels, A Dahl, J Londahl, T Hussein, E	2015	Quantification of differences between occupancy and total monitoring periods for better assessment of exposure to particles in indoor environments	Atmospheric Environment	106		419-428
	Switzerland	PM2.5				
Hanninen OO, E Lebret, V Ilacqua, K Katsouyanni, F Kunzli, RJ Sram, M Jantunen	2004	Infiltration of ambient PM2.5 and levels of indoor generated non-ETS PM2.5 in residences of four European cities	Atmospheric Environment	38	37	6411-6423

Kousa A, L Oglesby, K Koistinen, N Kunzli, M Jantunen	2002	Exposure chain of urban air PM2.5 - associations between ambient fixed site, residential outdoor, indoor, workplace and personal exposures in four European cities in the EXPOLIS-study	Atmospheric Environment	36	18	3031-3039
Meier R, M Eeftens, HC Phuleria, A Ineichen, E Corradi, M Davey, M Fierz,	2015	Differences in indoor versus outdoor concentrations of ultrafine particles, PM2.5, PMabsorbance and NO2 in Swiss homes	J Expo Sci Environ Epidemiol	25	5	499-505
Monn C, A Fuchs, D Hogger, M Junker, D Kogelschatz, N Roth, HU Wanner	1997	Particulate matter less than 10 mu m (PM10) and fine particles less than 2.5 mu m (PM2.5): relationships between indoor, outdoor and personal concentrations	Science of the Total Environment	208		15-21
Monn C, G Schaeppi	1993	CONCENTRATIONS OF TOTAL SUSPENDED PARTICULATES, FINE PARTICLES AND THEIR ANIONIC COMPOUNDS IN AMBIENT AIR AND INDOOR AIR	Environmental Technology	14	9	869-875
	UK	PM2.5				
de Hartog JJ, JG Ayres, A Karakatsani, A Analitis, H Brink, K Hameri, RM	2010	Lung function and indicators of exposure to indoor and outdoor particulate matter among asthma and COPD patients	Occupational and Environmental Medicine	67	1	43506
Dobson R, Semple S.	2018	How do you know those particles are from cigarettes?: An algorithm to help differentiate second-hand tobacco smoke from background sources of household fine particulate matter.	Environmental Research	166		344-7
Galea KS, JF Hurley, H Cowie, AL Shafrir, AS Jimenez, S Semple, JG	2013	Using PM2.5 concentrations to estimate the health burden from solid fuel combustion, with application to Irish and Scottish homes	Environmental Health	12		

Hoek G, G Kos, RM Harrison, J de Hartog, K Meliefste, H ten Brink, K	2008	Indoor-outdoor relationships of particle number and mass in four European cities	Atmospheric Environment	42	1	156-169
Jones NC, CA Thornton, D Mark, RM Harrison	2000	Indoor/outdoor relationships of particulate matter in domestic homes with roadside, urban and rural locations	Atmospheric Environment	34	16	2603-2612
Kingham S, D Briggs, P Elliott, P Fischer, E Lebret	2000	Spatial variations in the concentrations of traffic-related pollutants in indoor and outdoor air in Huddersfield, England	Atmospheric Environment	34	6	905-916
Lai HK, M Kendall, H Ferrier, I Lindup, S Alm, O Hanninen, M Jantunen, P	2004	Personal exposures and microenvironment concentrations of PM2.5, VOC, NO2 and CO in Oxford, UK	Atmospheric Environment	38	37	6399-6410
Mills LM, SE Semple, IS Wilson, L MacCalman, A Amos, D Ritchie, R	2012	Factors Influencing Exposure to Secondhand Smoke in Preschool Children Living With Smoking Mothers	Nicotine & Tobacco Research	14	12	1435-1444
Mohammadyan M, M Ashmore	2005	Personal exposure and indoor PM2.5 concentrations in an urban population	Indoor and Built Environment	14		313-320
Moreno-Rangel A, T Sharpe, F Musau, G McGill	2018	Field evaluation of a low-cost indoor air quality monitor to quantify exposure to pollutants in residential environments	Journal of Sensors and Sensor Systems	7	1	373-388
Mueller W, Steinle S, Parkka J, Parmes E, Liedes H, Kuijpers E, et al.	2020	Urban greenspace and the indoor environment: Pathways to health via indoor particulate matter, noise, and road noise annoyance.	Environmental Research	180		108850
Nasir ZA, I Colbeck	2013	Particulate pollution in different housing types in a UK suburban location	Science of the Total Environment	445		165-176

Osman LM, JG Douglas, C Garden, K Reglitz, J Lyon, S Gordon, JG Ayres	2007	Indoor air quality in homes of patients with chronic obstructive pulmonary disease	American Journal of Respiratory and Critical Care Medicine	176	5	465-472
Semple, S., Garden, C., Coggins, M., Galea, K.S., Whelan, P., Cowie, H.,	2012	Contribution of solid fuel, gas combustion, or tobacco smoke to indoor air pollutant concentrations in Irish and Scottish homes	Indoor air	22	3	212-223
Stewart L, IL Gee, AFR Watson, G Fletcher, R Niven	2001	Indoor air quality and childhood asthma: Method development and preliminary results	Indoor and Built Environment	10		271-275
Tan CCL, KN Finney, Q Chen, NV Russell, VN Sharifi, J Swithenbank	2013	Experimental Investigation of Indoor Air Pollutants in Residential Buildings	Indoor and Built Environment	22	3	471-489
Wigzell E, M Kendall, MJ Nieuwenhuijsen	2000	The spatial and temporal variation of particulate matter within the home	Journal of Exposure Analysis and Environmental Epidemiology	10	3	307-314
Wilson I, S Semple, LM Mills, D Ritchie, A Shaw, R O'Donnell, P Bonella, S	2013	REFRESHreducing families' exposure to secondhand smoke in the home: a feasibility study	Tobacco Control	22	5	
Woods KE, A Apsley, S Semple, SW Turner	2014	Domestic airborne fine particulate matter exposure and asthma control among children receiving inhaled steroid treatment	Indoor and Built Environment	23	3	497-503

PM2.5-10

East Asia PM2.5-10

China PM2.5-10

Cai J, Yu W, Li BZ, Yao RM, Zhang TJW, Guo M, et al.	2019	Particle removal efficiency of a household portable air cleaner in real-world residences: A single-blind cross-over field study.	Energy and Buildings	203		

	Japan	PM2.5-10				
Funasaka K, T Miyazaki, K Tsuruho, K Tamura, T Mizuno, K Kuroda	2000	Relationship between indoor and outdoor carbonaceous particulates in roadside households	Environmental Pollution	110	1	127-134
Michikawa T, S Nakai, H Nitta, K Tamura	2014	Validity of using annual mean particulate matter concentrations as measured at fixed site in assessing personal exposure: An exposure assessment study in Japan	Science of the Total Environment	466		673-680
Sugiyama T, T Amagai, H Matsushita, M Soma	1999	Size distribution of indoor airborne particulates collected by a low-flow rate cascade impactor	Indoor and Built Environment	8	6	361-369
Yoda Y, K Tamura, M Shima	2017	Airborne endotoxin concentrations in indoor and outdoor particulate matter and their predictors in an urban city	Indoor Air	27	5	955-964

Eastern Europe PM2.5-10

	Hungary	PM2.5-10				
Szirtesi K, A Angyal, Z Szoboszlai, E Furu, Z Torok, T Igaz, Z Kertesz	2018	Airborne Particulate Matter: An Investigation of Buildings with Passive House Technology in Hungary	Aerosol and Air Quality Research	18	5	1282-1293

North America PM2.5-10

	Canada	PM2.5-10				
Bari MA, M MacNeill, WB Kindzierski, L Wallace, ME Heroux, AJ Wheeler	2014	Predictors of coarse particulate matter and associated endotoxin concentrations in residential environments	Atmospheric Environment	92		221-230

Rasmussen PE, C Levesque, M Chenier, HD Gardner	2018	Contribution of metals in resuspended dust to indoor and personal inhalation exposures: Relationships between PM10 and settled dust	Building and Environment	143		513-522
Wheeler AJ, NA Dobbin, N Lyrette, L Wallace, M Foto, R Mallick, J Kearney, K Van	2011	Residential indoor and outdoor coarse particles and associated endotoxin exposures	Atmospheric Environment	45	39	7064-7071
	USA	PM2.5-10				
Abt E, HH Suh, G Allen, P Koutrakis	2000	Characterization of indoor particle sources: A study conducted in the metropolitan Boston area	Environmental Health Perspectives	108	1	35-44
Arhami M, A Polidori, RJ Delfino, T Tjoa, C Sioutas	2009	Associations between Personal, Indoor, and Residential Outdoor Pollutant Concentrations: Implications for Exposure Assessment to Size-Fractionated Particulate Matter	Journal of the Air & Waste Management Association	59	4	392-404
Butz AM, EC Matsui, P Breysse, J Curtin-Brosnan, P Eggleston, G Diette, D	2011	A Randomized Trial of Air Cleaners and a Health Coach to Improve Indoor Air Quality for Inner-City Children With Asthma and Secondhand Smoke Exposure	Archives of Pediatrics & Adolescent Medicine	165	8	741-748
Chen Q, LM Hildemann	2009	Size-Resolved Concentrations of Particulate Matter and Bioaerosols Inside versus Outside of Homes	Aerosol Science and Technology	43	7	699-713
Chen Q, LM Hildemann	2009	The Effects of Human Activities on Exposure to Particulate Matter and Bioaerosols in Residential Homes	Environmental Science & Technology	43	13	4641-4646
Delfino RJ, N Staimer, T Tjoa, A Polidori, M Arhami, DL Gillen, MT Kleinman, ND	2008	Circulating biomarkers of inflammation, antioxidant activity, and platelet activation are associated with primary combustion aerosols in subjects with coronary artery disease	Environmental Health Perspectives	116	7	898-906

Hsu SI, K Ito, M Kendall, M Lippmann	2012	Factors affecting personal exposure to thoracic and fine particles and their components	Journal of Exposure Science and Environmental Epidemiology	22	5	439-447
Kamens R, CT Lee, R Wiener, D Leith	1991	A STUDY TO CHARACTERIZE INDOOR PARTICLES IN 3 NONSMOKING HOMES	Atmospheric Environment Part a-General Topics	25		939-948
Kilburg-Basnyat B, TM Peters, SS Perry, PS Thorne	2016	Electrostatic dust collectors compared to inhalable samplers for measuring endotoxin concentrations in farm homes	Indoor Air	26	5	724-733
Leaderer BP, L Naeher, T Jankun, K Balenger, TR Holford, C Toth, J Sullivan,	1999	Indoor, outdoor, and regional summer and winter concentrations of PM10, PM2.5, SO42-, H+, NH4+, NO3-, NH3, and nitrous acid in homes with and without kerosene space heaters	Environmental Health Perspectives	107	3	223-231
Less, B., Mullen, N., Singer, B., & Walker, I	2015	Indoor air quality in 24 California residences designed as high-performance homes	Science and Technology for the Built Environment	21	1	14-24
Long CM, HH Suh, P Koutrakis	2000	Characterization of indoor particle sources using continuous mass and size monitors	Journal of the Air & Waste Management Association	50	7	1236-1250
McCormack MC, PN Breysse, EC Matsui, NN Hansel, D Williams, J	2009	In-Home Particle Concentrations and Childhood Asthma Morbidity	Environmental Health Perspectives	117	2	294-298
Rojas-Bracho L, HH Suh, P Koutrakis	2000	Relationships among personal, indoor, and outdoor fine and coarse particle concentrations for individuals with COPD	Journal of Exposure Analysis and Environmental Epidemiology	10	3	294-306
Williams R, AG Rappold, M Case, M Schmitt, S Stone, P Jones, J Thornburg, RB	2012	Multi-pollutant exposures in an asthmatic cohort	Atmospheric Environment	61		244-252

Williams R, J Creason, R Zweidinger, R Watts, L Sheldon, C Shy	2000	Indoor, outdoor, and personal exposure monitoring of particulate air pollution: the Baltimore elderly epidemiology-exposure pilot study	Atmospheric Environment	34	24	4193-4204
Williams R, J Suggs, A Rea, K Leovic, A Vette, C Croghan, L Sheldon, C	2003	The Research Triangle Park particulate matter panel study: PM mass concentration relationships	Atmospheric Environment	37	38	5349-5363
Williams, R.O.N., Rea, A., Vette, A., Croghan, C., Whitaker, D., Stevens, C.,	2009	The design and field implementation of the Detroit Exposure and Aerosol Research Study	Journal of Exposure Science and Environmental Epidemiology	19	7	643-659

South America PM2.5-10

Brazil PM2.5-10

Segalin B, P Kumar, K Micadei, A Fornaro, FLT Goncalves	2017	Size-segregated particulate matter inside residences of elderly in the Metropolitan Area of Sao Paulo, Brazil	Atmospheric Environment	148		139-151

Chile PM2.5-10

Rojas-Bracho L, HH Suh, P Oyola, P Koutrakis	2002	Measurements of children's exposures to particles and nitrogen dioxide in Santiago, Chile	Science of the Total Environment	287	3	249-264

Ecuador PM2.5-10

Raysoni AU, RX Armijos, MM Weigel, T Montoya, P Eschanique, M Racines,	2016	Assessment of indoor and outdoor PM species at schools and residences in a high-altitude Ecuadorian urban center	Environmental Pollution	214		668-679

South Asia PM2.5-10

India PM2.5-10

Khillare PS, R Pandey, S Balachandran	2004	Characterisation of indoor PM10 in residential areas of Delhi	Indoor and Built Environment	13	2	139-147
Kumar P	2001	Characterisation of indoor respirable dust in a locality of Delhi, India	Indoor and Built Environment	10	2	95-102

Pakistan PM2.5-10

Nasir, Z. A., Colbeck, I., Ali, Z., & Ahmad, S	2013	Indoor particulate matter in developing countries: a case study in Pakistan and potential intervention strategies	Environmental Research Letters	8	2	024002

West Asia & North Africa PM2.5-10

United Arab Emirates PM2.5-10

Yeatts KB, M El-Sadig, D Leith, W Kalsbeek, F Al-Maskari, D Couper, WE	2012	Indoor Air Pollutants and Health in the United Arab Emirates	Environmental Health Perspectives	120	5	687-694

Western Europe PM2.5-10

Finland PM2.5-10

Hoek G, G Kos, RM Harrison, J de Hartog, K Meliefste, H ten Brink, K	2008	Indoor-outdoor relationships of particle number and mass in four European cities	Atmospheric Environment	42	1	156-169

Greece PM2.5-10

Hoek G, G Kos, RM Harrison, J de Hartog, K Meliefste, H ten Brink, K	2008	Indoor-outdoor relationships of particle number and mass in four European cities	Atmospheric Environment	42	1	156-169

	Netherlands	PM2.5-10				
Hoek G, G Kos, RM Harrison, J de Hartog, K Meliefste, H ten Brink, K	2008	Indoor-outdoor relationships of particle number and mass in four European cities	Atmospheric Environment	42	1	156-169
	Norway	PM2.5-10				
Lazaridis M, V Aleksandropoulou, JE Hanssen, C Dye, K	2008	Inorganic and carbonaceous components in indoor/outdoor particulate matter in two residential houses in Oslo, Norway	Journal of the Air & Waste Management Association	58	3	346-356
	Portugal	PM2.5-10				
Cerqueira M, D Marques, A Caseiro, C Pio	2010	Experimental evidence for a significant contribution of cellulose to indoor aerosol mass concentration	Atmospheric Environment	44	6	867-871
	UK	PM2.5-10				
Hoek G, G Kos, RM Harrison, J de Hartog, K Meliefste, H ten Brink, K	2008	Indoor-outdoor relationships of particle number and mass in four European cities	Atmospheric Environment	42	1	156-169
Nasir ZA, I Colbeck	2013	Particulate pollution in different housing types in a UK suburban location	Science of the Total Environment	445		165-176

PM2-10

East Asia PM2-10

	Japan	PM2-10				
Funasaka K, T Miyazaki, K Tsuruho, K Tamura, T Mizuno, K Kuroda	2000	Relationship between indoor and outdoor carbonaceous particulates in roadside households	Environmental Pollution	110	1	127-134

PM3.0

North America PM3.0

USA PM3.0

Fuller CH, D Brugge, PL Williams, MA Mittleman, K Lane, JL Durant, JD Spengler	2013	Indoor and outdoor measurements of particle number concentration in near-highway homes	Journal of Exposure Science and Environmental Epidemiology	23	5	506-512

PM3.5

East Asia PM3.5

China PM3.5

Hu YJ, LJ Bao, CL Huang, SM Li, P Liu, EY Zeng	2018	Exposure to air particulate matter with a case study in Guangzhou: Is indoor environment a safe haven in China?	Atmospheric Environment	191		351-359

South Korea PM3.5

Baek SO, YS Kim, R Perry	1997	Indoor air quality in homes, offices and restaurants in Korean urban areas - Indoor/outdoor relationships	Atmospheric Environment	31	4	529-544

South East Asia PM3.5

Singapore PM3.5

Lai ACK, YW Ho	2008	Spatial concentration variation of cooking-emitted particles in a residential kitchen	Building and Environment	43	5	871-876

Western Europe PM3.5

Germany PM3.5

Phillips K, DA Howard, MC Bentley, G Alvan	1998	Measured exposures by personal monitoring for respirable suspended particles and environmental tobacco smoke of housewives and office workers resident in Bremen, Germany	Int Arch Occup Environ Health	71	3	201-12

Switzerland PM3.5

Phillips K, DA Howard, MC Bentley, G Alvan	1999	Assessment of environmental tobacco smoke and respirable suspended particle exposures for nonsmokers in Basel by personal monitoring	Atmospheric Environment	33	12	1889-1904

PM4

Africa (except North) PM4

Ethiopia PM4

Embiale A, Zewge F, Chandravanshi BS, Sahle-Demessie E.	2019	Short-term exposure assessment to particulate matter and total volatile organic compounds in indoor air during cooking Ethiopian sauces (Wot) using electricity, kerosene and charcoal fuels.	Indoor and Built Environment	28	8	1140-54

South Africa PM4

Adesina JA, Piketh SJ, Qhekwana M, Burger R, Language B, Mkhatshwa G.	2020	Contrasting indoor and ambient particulate matter concentrations and thermal comfort in coal and non-coal burning households at South Africa Highveld.	Science of the total environment	699	10	134403

East Asia PM4

China PM4

Huang C, X Wang, W Liu, J Cai, L Shen, Z Zou, R Lu, J Chang, X Wei, C Sun, Z	2016	Household indoor air quality and its associations with childhood asthma in Shanghai, China: On-site inspected methods and preliminary results	Environ Res	151		154-167

North America PM4

Canada PM4

Weichenthal S, A Dufresne, C Infante-Rivard, L Joseph	2007	Indoor ultrafine particle exposures and home heating systems: A cross-sectional survey of Canadian homes during the winter months	Journal of Exposure Science and Environmental Epidemiology	17	3	288-297

USA PM4

Patton AP, L Calderon, YY Xiong, ZC Wang, J Senick, MS Allacci, D Plotnik, R	2016	Airborne Particulate Matter in Two Multi-Family Green Buildings: Concentrations and Effect of Ventilation and Occupant Behavior	International Journal of Environmental Research and Public Health	13	1	

South Asia PM4

Bangladesh PM4

Akther T, Ahmed M, Shohel M, Ferdousi FK, Salam A.	2019	Particulate matters and gaseous pollutants in indoor environment and Association of ultra-fine particulate matters (PM1) with lung function.		26	6	5475-84.

Western Europe PM4

Germany PM4

Fromme H, T Lahrz, A Hainsch, A Oddoy, M Piloty, H Ruden	2005	Elemental carbon and respirable particulate matter in the indoor air of apartments and nursery schools and ambient air in Berlin (Germany)	Indoor Air	15	5	335-341

Weschler CJ, T Salthammer, H Fromme	2008	Partitioning of phthalates among the gas phase, airborne particles and settled dust in indoor environments	Atmospheric Environment	42	7	1449-1460

PM5

North America PM5

USA PM5

Kopperud RJ, AR Ferro, LM Hildemann	2004	Outdoor versus indoor contributions to indoor particulate matter (PM) determined by mass balance methods	Journal of the Air & Waste Management Association	54	9	1188-1196

South Asia PM5

India PM5

Kulshrestha A, DS Bisht, J Masih, D Massey, S Tiwari, A Taneja	2009	Chemical characterization of water-soluble aerosols in different residential environments of semi aridregion of India	Journal of Atmospheric Chemistry	62	2	121-138
Massey D, A Kulshrestha, J Masih, A Taneja	2012	Seasonal trends of PM10, PM5.0, PM2.5 & PM1.0 in indoor and outdoor environments of residential homes located in North-Central India	Building and Environment	47		223-231
Saksena S, RK Prasad, VR Shankar	2007	Daily exposure to air pollutants in indoor, outdoor and in-vehicle microenvironments: A pilot study in Delhi	Indoor and Built Environment	16	1	39-46

West Asia & North Africa PM5

Israel PM5

Jodeh S, AR Hasan, J Amarah, F Judeh, R Salghi, H Lgaz, W Jodeh	2018	Indoor and outdoor air quality analysis for the city of Nablus in Palestine: seasonal trends of PM10, PM5.0, PM2.5, and PM1.0 of residential homes	Air Quality Atmosphere and Health	11	2	229-237

Western Europe PM5

Portugal PM5

Almeida-Silva M, Pilou M, Housiadas C, Almeida SM.	2018	Internal dose of particles in the elderly—modeling based on aerosol measurements.	Environmental Science and Pollution Research	25	24	23645-56

UK PM5

O'Connell S, HKC Au-Yeung, CJ Gregory, IP Matthews	2008	Outdoor and indoor respirable air particulate concentrations in differing urban traffic microenvironments	Journal of Toxicology and Environmental Health-Part a-Current Issues	71	16	1069-1072

Surface Area

Western Europe Surface Area

Portugal Surface Area

Almeida-Silva M, SM Almeida, JF Gomes, PC Albuquerque, HT	2014	Determination of airborne nanoparticles in elderly care centers	J Toxicol Environ Health A	77		867-78

TSP

Africa (except North) TSP

Ethiopia TSP

Embiale A, Zewge F, Chandravanshi BS, Sahle-Demessie E.	2019	Short-term exposure assessment to particulate matter and total volatile organic compounds in indoor air during cooking Ethiopian sauces (Wot) using electricity, kerosene and charcoal fuels.	Indoor and Built Environment	28	8	1140-54

East Asia TSP

China TSP

Cai J, Yu W, Li BZ, Yao RM, Zhang TJW, Guo M, et al.	2019	Particle removal efficiency of a household portable air cleaner in real-world residences: A single-blind cross-over field study.	Energy and Buildings	203		
Chao CYH, TCW Tung, J Burnett	1998	Influence of different indoor activities on the indoor particulate levels in residential buildings	Indoor and Built Environment	7	2	110-121
Tung TCW, CYH Chao, J Burnett, SW Pang, RYM Lee	1999	A territory wide survey on indoor particulate level in Hong Kong	Building and Environment	34	2	213-220
Wu YJ, Li GY, Yang Y, An TC.	2019	Pollution evaluation and health risk assessment of airborne toxic metals in both indoors and outdoors of the Pearl River Delta, China.	Environmental Research	179		Epub
Xu F, W Tang, W Zhang, L Liu, K Lin	2016	Levels, distributions and correlations of polybrominated diphenyl ethers in air and dust of household and workplace in Shanghai, China: implication for daily human exposure	Environ Sci Pollut Res Int	23	4	3229-38

Japan TSP

Ando M, K Tamura, M Matsumoto	1990	[The suspended particulate matter (SPM) and polycyclic aromatic hydrocarbons (PAHs) in indoor and outdoor air along a main road]	Nihon Eiseigaku Zasshi	45	5	1007-13
Sugiyama T, T Amagai, H Matsushita, M Soma	1999	Size distribution of indoor airborne particulates collected by a low-flow rate cascade impactor	Indoor and Built Environment	8	6	361-369

Taiwan TSP

Li CS	1994	RELATIONSHIPS OF INDOOR OUTDOOR INHALABLE AND RESPIRABLE PARTICLES IN DOMESTIC ENVIRONMENTS	Science of the Total Environment	151	3	205-211

North America TSP

Canada TSP

Guggisberg M, PA Hessel, D Michaelchuk, M Atiemo	2003	Particulate matter and gaseous contaminants in indoor environments in an isolated northern community	Int J Circumpolar Health	62	2	120-9

USA TSP

Batterman S, C Godwin, CR Jia	2005	Long duration tests of room air filters in cigarette smokers' homes	Environmental Science & Technology	39	18	7260-7268
Batterman S, L Du, G Mentz, B Mukherjee, E Parker, C Godwin, JY Chin,	2012	Particulate matter concentrations in residences: an intervention study evaluating stand-alone filters and air conditioners	Indoor Air	22	3	235-252
Chen Q, LM Hildemann	2009	Size-Resolved Concentrations of Particulate Matter	Aerosol Science and Technology	43	7	699-713

		and Bioaerosols Inside versus Outside of Homes				
LeBouf R, L Yesse, A Rossner	2008	Seasonal and diurnal variability in airborne mold from an indoor residential environment in northern New York	Journal of the Air & Waste Management Association	58	5	684-692
Williams DL, PN Breysse, MC McCormack, GB Diette, S McKenzie, AS Geyh	2011	Airborne cow allergen, ammonia and particulate matter at homes vary with distance to industrial scale dairy operations: an exposure assessment	Environmental Health	10		

Oceania TSP

Australia TSP

Jones J, S Stick, P Dingle, P Franklin	2007	Spatial variability of particulates in homes: Implications for infant exposure	Science of the Total Environment	376		317-323

South Asia TSP

Bangladesh TSP

Akther T, Ahmed M, Shohel M, Ferdousi FK, Salam A.	2019	Particulate matters and gaseous pollutants in indoor environment and Association of ultra-fine particulate matters (PM1) with lung function.		26	6	5475-84.

India TSP

Gupta S, A Srivastava, VK Jain	2008	Particle size distribution of aerosols and associated heavy metals in kitchen environments	Environ Monit Assess	142		141-8

West Asia & North Africa TSP

Egypt TSP

Abdel-Salam MMM	2012	Indoor Particulate Matter in Different Residential Areas of Alexandria City, Egypt	Indoor and Built Environment	21	6	857-862

Oman TSP

Abdul-Wahab SA	2006	Indoor and outdoor relationships of atmospheric particulates in Oman	Indoor and Built Environment	15	3	247-255

Western Europe TSP

France TSP

Maesano CN, Caillaud D, Youssouf H, Banerjee S, Prud'Homme J, Audi C, et	2019	Indoor exposure to particulate matter and volatile organic compounds in dwellings and workplaces and respiratory health in French farmers.	Multidisciplinary Respiratory Medicine	14	1	eCollection

Germany TSP

Berger-Preiss E, K Levsen, G Leng, H Idel, D Sugiri, U Ranft	2002	Indoor pyrethroid exposure in homes with woollen textile floor coverings	Int J Hyg Environ Health	205	6	459-72
Moriske HJ, M Drews, G Ebert, G Menk, C Scheller, M Schondube, L Konieczny	1996	Indoor air pollution by different heating systems: Coal burning, open fireplace and central heating	Toxicology Letters	88		349-354
Muller J	1991	INDOOR AND OUTDOOR AIR MEASUREMENTS AT A CITY STREET WITH HEAVY TRAFFIC	Staub Reinhaltung Der Luft	51	4	147-154

UK TSP

Halsall CJ, BA Maher, VV Karloukovski, P Shah, SJ	2008	A novel approach to investigating indoor/outdoor pollution links: Combined magnetic and PAH	Atmospheric Environment	42	39	8902-8909

Watkins		measurements				
O'Connell S, HKC Au-Yeung, CJ Gregory, IP Matthews	2008	Outdoor and indoor respirable air particulate concentrations in differing urban traffic microenvironments	Journal of Toxicology and Environmental Health-Part a-Current Issues	71	16	1069-1072

UFP (<0.3 µm)

East Asia UFP (<0.3)

China UFP (<0.3)

Hu YJ, LJ Bao, CL Huang, SM Li, P Liu, EY Zeng	2018	Exposure to air particulate matter with a case study in Guangzhou: Is indoor environment a safe haven in China?	Atmospheric Environment	191		351-359
Mazaheri M, Lin WW, Clifford S, Yue DL, Zhai YH, Xu MW, et al.	2019	Characteristics of school children's personal exposure to ultrafine particles in Heshan, Pearl River Delta, China - A pilot study.	Environment International	132		Epub
Spickett J, K Rumchev, H Jing	2014	The Domestic Environment and Respiratory Health of School Children in Zongshan, China	Asia-Pacific Journal of Public Health	26	6	596-603
Sun Y, Hou J, Cheng R, Sheng Y, Zhang X, Sundell J.	2019	Indoor air quality, ventilation and their associations with sick building syndrome in Chinese homes.	Energy and Buildings	197	15	112-9

Eastern Europe UFP (<0.3)

Poland UFP (<0.3)

Bentayeb M, D Norback, M Bednarek, A Bernard, GH Cai, S Cerrai, KK	2015	Indoor air quality, ventilation and respiratory health in elderly residents Living in nursing homes in Europe	European Respiratory Journal	45	5	1228-1238

North America UFP (<0.3)

Canada UFP (<0.3)

Jeong CH, Salehi S, Wu J, North ML, Kim JS, Chow CW, et al.	2019	Indoor measurements of air pollutants in residential houses in urban and suburban areas: Indoor versus ambient concentrations.	Science of The Total Environment	693		133446
Kearney J, L Wallace, M MacNeill, ME Heroux, W Kindzierski, A Wheeler	2014	Residential infiltration of fine and ultrafine particles in Edmonton	Atmospheric Environment	94		793-805
Kearney J, L Wallace, M MacNeill, X Xu, K VanRyswyk, H You, R Kulka,	2011	Residential indoor and outdoor ultrafine particles in Windsor, Ontario	Atmospheric Environment	45	40	7583-7593
Weichenthal S, A Dufresne, C Infante-Rivard, L Joseph	2007	Indoor ultrafine particle exposures and home heating systems: A cross-sectional survey of Canadian homes during the winter months	Journal of Exposure Science and Environmental Epidemiology	17	3	288-297

USA UFP (<0.3)

Arhami M, A Polidori, RJ Delfino, T Tjoa, C Sioutas	2009	Associations between Personal, Indoor, and Residential Outdoor Pollutant Concentrations: Implications for Exposure Assessment to Size-Fractionated Particulate Matter	Journal of the Air & Waste Management Association	59	4	392-404
Bhangar S, NA Mullen, SV Hering, NM Kreisberg, WW Nazaroff	2011	Ultrafine particle concentrations and exposures in seven residences in northern California	Indoor Air	21	2	132-144
Cox J, K Isiugo, P Ryan, SA Grinshpun, M Yermakov, C Desmond, R Jandarov, S	2018	Effectiveness of a portable air cleaner in removing aerosol particles in homes close to highways	Indoor Air	28	6	818-827

Shalat SL, AA Stambler, Z Wang, G Mainelis, OH Emoekpere, M Hernandez,	2011	Development and in-home testing of the Pretoddler Inhalable Particulate Environmental Robotic (PIPER Mk IV) sampler	Environ Sci Technol	45	7	2945-50
Thomas NM, Calderón L, Senick J, Sorensen-Allacci M, Plotnik D, Guo M, et al.	2019	Investigation of indoor air quality determinants in a field study using three different data streams.	Building and Environment	154		281-95
Waring MS, JA Siegel	2011	The effect of an ion generator on indoor air quality in a residential room	Indoor Air	21	4	267-276

Oceania UFP (<0.3)

Australia UFP (<0.3)

Mazaheri M, S Clifford, R Jayaratne, MA Megat Mokhtar, F Fuoco, G	2014	School children's personal exposure to ultrafine particles in the urban environment	Environ Sci Technol	48	1	113-20

South America UFP (<0.3)

Chile UFP (<0.3)

Ruiz PA, C Toro, J Caceres, G Lopez, P Oyola, P Koutrakis	2010	Effect of Gas and Kerosene Space Heaters on Indoor Air Quality: A Study in Homes of Santiago, Chile	Journal of the Air & Waste Management Association	60	1	98-108

South East Asia UFP (<0.3)

Singapore UFP (<0.3)

Sharm R, R Balasubramanian	2018	Size-fractionated Particulate Matter in Indoor and Outdoor Environments during the 2015 Haze in Singapore: Potential Human Health Risk Assessment	Aerosol and Air Quality Research	18	4	904-917

Western Europe UFP (<0.3)

Belgium UFP (<0.3)

Bentayeb M, D Norback, M Bednarek, A Bernard, GH Cai, S Cerrai, KK	2015	Indoor air quality, ventilation and respiratory health in elderly residents Living in nursing homes in Europe	European Respiratory Journal	45	5	1228-1238

Denmark UFP (<0.3)

Beko G, BU Kjeldsen, Y Olsen, J Schipperijn, A Wierzbicka, DG Karottki, J	2015	Contribution of various microenvironments to the daily personal exposure to ultrafine particles: Personal monitoring coupled with GPS tracking	Atmospheric Environment	110		122-129
Beko G, CJ Weschler, A Wierzbicka, DG Karottki, J Toftum, S Loft, G Clausen	2013	Ultrafine Particles: Exposure and Source Apportionment in 56 Danish Homes	Environmental Science & Technology	47	18	10240-
Bentayeb M, D Norback, M Bednarek, A Bernard, GH Cai, S Cerrai, KK	2015	Indoor air quality, ventilation and respiratory health in elderly residents Living in nursing homes in Europe	European Respiratory Journal	45	5	1228-1238
Jantzen K, P Moller, DG Karottki, Y Olsen, G Beko, G Clausen, LG Hersoug, S Loft	2016	Exposure to ultrafine particles, intracellular production of reactive oxygen species in leukocytes and altered levels of endothelial progenitor cells	Toxicology	359		43787
Karottki DG, G Beko, G Clausen, AM Madsen, ZJ Andersen, A Massling, M	2014	Cardiovascular and lung function in relation to outdoor and indoor exposure to fine and ultrafine particulate matter in middle-aged subjects	Environment International	73		372-381
Karottki DG, M Spilak, M Frederiksen, ZJ Andersen, AM Madsen, M Ketzel, A	2015	Indoor and Outdoor Exposure to Ultrafine, Fine and Microbiologically Derived Particulate Matter Related to Cardiovascular and Respiratory Effects in a Panel of Elderly Urban Citizens	International Journal of Environmental Research and Public Health	12	2	1667-1686

Olsen Y, DG Karottki, DM Jensen, G Beko, BU Kjeldsen, G Clausen, LG	2014	Vascular and lung function related to ultrafine and fine particles exposure assessed by personal and indoor monitoring: a cross-sectional study	Environmental Health	13		
Spilak MP, M Frederiksen, B Kolarik, L Gunnarsen	2014	Exposure to ultrafine particles in relation to indoor events and dwelling characteristics	Building and Environment	74		65-74
	Finland	UFP (<0.3)				
Sippula O, H Rintala, M Happo, P Jalava, K Kuuspalo, A Viren, A	2013	Characterization of Chemical and Microbial Species from Size-Segregated Indoor and Outdoor Particulate Samples	Aerosol and Air Quality Research	13	4	1212-U343
	France	UFP (<0.3)				
Bentayeb M, D Norback, M Bednarek, A Bernard, GH Cai, S Cerrai, KK	2015	Indoor air quality, ventilation and respiratory health in elderly residents Living in nursing homes in Europe	European Respiratory Journal	45	5	1228-1238
Maesano CN, Caillaud D, Youssouf H, Banerjee S, Prud'Homme J, Audi C, et	2019	Indoor exposure to particulate matter and volatile organic compounds in dwellings and workplaces and respiratory health in French farmers.	Multidisciplinary Respiratory Medicine	14	1	eCollection
Paunescu AC, M Attoui, S Bouallala, J Sunyer, I Momas	2017	Personal measurement of exposure to black carbon and ultrafine particles in schoolchildren from PARIS cohort (Paris, France)	Indoor Air	27	4	766-779
	Germany	UFP (<0.3)				
Santen M, M Wesselmann, U Fittschen, R Cremer, P Braun, A Ludecke, HJ	2009	Measurements of fine and ultrafine particles in indoor environment of living rooms	Gefahrstoffe Reinhaltung Der Luft	69	3	63-70
	Greece	UFP (<0.3)				
Bentayeb M, D Norback, M Bednarek, A Bernard, GH Cai, S Cerrai, KK	2015	Indoor air quality, ventilation and respiratory health in elderly residents Living in nursing homes in Europe	European Respiratory Journal	45	5	1228-1238

Diapouli E, A Chaloulakou, N Spyrellis	2007	Levels of ultrafine particles in different microenvironments - Implications to children exposure	Science of the Total Environment	388		128-136

Italy UFP (<0.3)

Bentayeb M, D Norback, M Bednarek, A Bernard, GH Cai, S Cerrai, KK	2015	Indoor air quality, ventilation and respiratory health in elderly residents Living in nursing homes in Europe	European Respiratory Journal	45	5	1228-1238
Buonanno G, S Marini, L Morawska, FC Fuoco	2012	Individual dose and exposure of Italian children to ultrafine particles	Science of the Total Environment	438		271-277
Zauli-Sajani S, S Rovelli, A Trentini, D Bacco, S Marchesi, F Scotto, C	2018	Higher health effects of ambient particles during the warm season: The role of infiltration factors	Science of the Total Environment	627		67-77

Netherlands UFP (<0.3)

de Kluizenaar Y, E Kuijpers, I Eekhout, M Voogt, RCH Vermeulen, G Hoek, RP	2017	Personal exposure to UFP in different micro-environments and time of day	Building and Environment	122		237-246

Sweden UFP (<0.3)

Bentayeb M, D Norback, M Bednarek, A Bernard, GH Cai, S Cerrai, KK	2015	Indoor air quality, ventilation and respiratory health in elderly residents Living in nursing homes in Europe	European Respiratory Journal	45	5	1228-1238
Isaxon C, A Gudmundsson, EZ Nordin, L Lonnblad, A Dahl, G Wieslander, M	2015	Contribution of indoor-generated particles to residential exposure	Atmospheric Environment	106		458-466
Matson U	2005	Indoor and outdoor concentrations of ultrafine particles in some Scandinavian rural and urban areas	Science of the Total Environment	343		169-176

References for Supplemental Information

Li, Z., Wen, Q., & Zhang, R. (2017). Sources, health effects and control strategies of indoor fine particulate matter (PM2. 5): A review. *Science of the Total Environment*, *586*, 610–622. https://doi.org/10.1016/j.scitotenv.2017.02.029

Logue, J. M., McKone, T. E., Sherman, M. H., & Singer, B. C. (2011). Hazard assessment of chemical air contaminants measured in residences. *Indoor Air*, *21*(2), 92–109.

Morawska, L., Afshari, A., Bae, G. N., Buonanno, G., Chao, C. Y. H., Hänninen, O., Hofmann, W., Isaxon, C., Jayaratne, E. R., & Pasanen, P. (2013). Indoor aerosols: from personal exposure to risk assessment. *Indoor Air*, *23*(6), 462–487.

Morawska, L., Ayoko, G. A., Bae, G. N., Buonanno, G., Chao, C. Y. H., Clifford, S., Fu, S. C., Hänninen, O., He, C., Isaxon, C., Mazaheri, M., Salthammer, T., Waring, M. S., & Wierzbicka, A. (2017). Airborne particles in indoor environment of homes, schools, offices and aged care facilities: The main routes of exposure. *Environment International*, *108*, 75–83. https://doi.org/https://doi.org/10.1016/j.envint.2017.07.025

Vardoulakis, S., Giagloglou, E., Steinle, S., Davis, A., Sleeuwenhoek, A., Galea, K. S., Dixon, K., & Crawford, J. O. (2020). Indoor Exposure to Selected Air Pollutants in the Home Environment: A Systematic Review. *International Journal of Environmental Research and Public Health*, *17*(23). https://doi.org/10.3390/ijerph17238972

Ye, W., Zhang, X., Gao, J., Cao, G., Zhou, X., & Su, X. (2017). Indoor air pollutants, ventilation rate determinants and potential control strategies in Chinese dwellings: A literature review. *Science of The Total Environment*, *586*, 696–729. https://doi.org/https://doi.org/10.1016/j.scitotenv.2017.02.047
